# Local Functioning, Landscape Structuring: Drivers of Soil Microbial Community Structure and Function in Peatlands

**DOI:** 10.3389/fmicb.2018.02060

**Published:** 2018-09-03

**Authors:** Sven Teurlincx, Amber Heijboer, Annelies J. Veraart, George A. Kowalchuk, Steven A. J. Declerck

**Affiliations:** ^1^Department of Aquatic Ecology, Netherlands Institute of Ecology, Wageningen, Netherlands; ^2^Biometris, Wageningen University, Wageningen, Netherlands; ^3^Institute of Environmental Biology, Utrecht University, Utrecht, Netherlands; ^4^Department of Aquatic Ecology and Environmental Biology, Radboud University Nijmegen, Nijmegen, Netherlands; ^5^Department of Microbial Ecology, Netherlands Institute of Ecology, Wageningen, Netherlands

**Keywords:** microbial community, landscape ecology, PLFA, peatlands, ditch margins, CLPP, Biolog Ecoplates, peatland management

## Abstract

Agricultural peatlands are essential for a myriad of ecosystem functions and play an important role in the global carbon (C) cycle through C sequestration. Management of these agricultural peatlands takes place at different spatial scales, ranging from local to landscape management, and drivers of soil microbial community structure and function may be scale-dependent. Effective management for an optimal biogeochemical functioning thus requires knowledge of the drivers on soil microbial community structure and functioning, as well as the spatial scales upon which they are influenced. During two field campaigns, we examined the importance of different drivers (i.e., soil characteristics, nutrient management, vegetation composition) at two spatial scales (local vs. landscape) for, respectively, the soil microbial community structure (determined by PLFA) and soil microbial community functional capacity (as assessed by CLPP) in agricultural peatlands. First, we show by an analysis of PLFA profiles that the total microbial biomass changes with soil moisture and relative C:P nutrient availability. Secondly, we showed that soil communities are controlled by a distinct set of drivers at the local, as opposed to landscape, scale. Community structure was found to be markedly different between areas, in contrast to community function which showed high variability within areas. We further found that microbial structure appears to be controlled more at a landscape scale by nutrient-related variables, whereas microbial functional capacity is driven locally through plant community feedbacks. Optimal management strategies within such peatlands should therefore consider the scale-dependent action of soil microbial community drivers, for example by first optimizing microbial structure at the landscape scale by targeted areal management, and then optimizing soil microbial function by local vegetation management.

## Introduction

Peatlands play an important role in Earth’s biogeochemical cycles by storing about an estimated third of all terrestrial carbon (C) (Turetsky et al., 2002; Turunen et al., 2002). In Europe, the majority of peatlands is in use as agricultural land (Joosten and Clarke, 2002). Despite their potential to sequester C, agricultural peatlands typically act as significant C sources. Worldwide drainage of such peatlands has increased the rates of peat oxidation and hence microbial decomposition, causing high rates of C losses and greenhouse gas emissions (Drösler et al., 2008). However, due to the large C sequestration potential of agricultural peatlands, they could play an important role in efforts to increase soil C storage, such as the recently launched ‘4 per 1000 initiative,’ which seeks to increase C storage in agricultural soils with 4‰ per year (Le Foll, 2015).

Current peatland management influences microbe-mediated biogeochemical functions, for example by maintaining waterlogged conditions to prevent microbial peat oxidation and thereby reduce peat subsidence and CO_2_ emissions (Kløve et al., 2017). Restoration of peat ditches often seeks to optimize nutrient removal and reduce eutrophication, both of which have links to the microbial processes of nitrogen (N) and phosphorus (P) conversion. Microbial activities are clearly critical to the success of peatland management strategies, for instance for C storage, yet management practices rarely consider potential impacts on soil-borne microbial communities. With future climate change pressures in mind, the management of ecosystems for minimal microbial mediated CH_4_ and N_2_O emission is expected to become ever more important (Taft et al., 2017).

Traditionally regarded as random noise, spatial variability in soil microbial communities is now widely acknowledged (Ettema and Wardle, 2002), and it displays consistent and informative patterns at different spatial scales (O’Brien et al., 2016). With mounting evidence for scale-dependent ecological processes acting on microbial communities, the need for examining multiple spatial scales to understand the patterns in soil microbial communities has become apparent (Martiny et al., 2011). This has led to the study and discovery of clear examples of small scale (cm to m) patterns (e.g., Franklin and Mills, 2003), as well as large continental (Waldrop et al., 2017) and global biogeographic patterns in soil microbial communities (Nemergut et al., 2011). These scales of study are not always in line with the scales at which the management of such ecosystems takes place. For agricultural peatlands, the most obvious scale of management is that of the field level, with local farmers carrying out customary management practices such as fertilization, grazing and mowing. Another relevant scale is that of the landscape level at which spatial planning and water level management occurs. Furthermore, the different components of the ecosystem may also be organized at different spatial scales themselves (Yergeau et al., 2010). To adequately steer management toward optimization of microbial communities, there is a need to match the spatial scale of land management and the study of spatial microbial community patterns.

While identification of patterns in soil microbial composition is in itself relevant, there is a clear need to go beyond pattern description and toward identification of the underlying drivers of community structure and functioning (Martiny et al., 2011; Hanson et al., 2012). Soil microbial community structure and functioning are often assumed to be driven by the same factors (O’Brien et al., 2016). This would imply that management aimed at an optimal microbial structure will also result in the desired functioning of the ecosystem. Alternatively, soil microbes are often considered to have high functional redundancy (Strickland et al., 2009); and therefore drivers of soil microbial community structure may have minimal effect on soil microbial functioning. This perspective would imply that management practices designed to control only the drivers of community function would be sufficient to achieve the desired ecosystem functions.

Soil microbial community structure has been shown to be controlled by a wide spectrum of drivers: soil pH and C:N ratio (Lauber et al., 2008; Fierer et al., 2009, 2012; Kuramae et al., 2012; Zhang et al., 2013; Ramirez et al., 2014), vegetation (O’Donnell et al., 2001; de Vries et al., 2012), external nutrient load (O’Donnell et al., 2001; O’Brien et al., 2016), and soil moisture (Brockett et al., 2012). Although drivers of soil microbial community function have not been examined in as much detail, but it has been shown that soil microbial community functions are also controlled by soil moisture (Brockett et al., 2012), C:N ratio (Kuramae et al., 2014), and external nutrient load and pH (Wakelin et al., 2013). These different drivers of community structure and functioning differ with the spatial scale of examination. As land management is carried out at scales orders of magnitude larger than those experienced by microbes directly, it remains to be tested if and how microbial communities respond to changes in these drivers. If soil microbial community structure and functioning are influenced by scale-dependent drivers, information on the scale-dependency of these microbial community drivers could be useful for informing management designed to improve peatland functioning.

**FIGURE 1:**
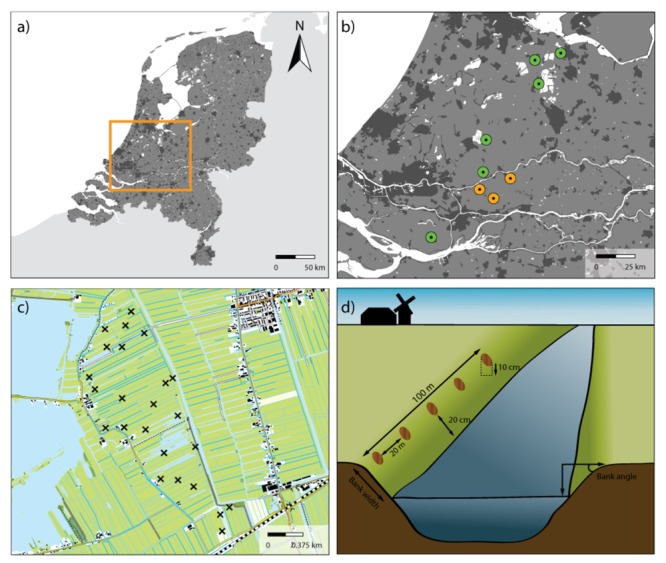
Overview of sample areas and sample sites. **(a)** The Netherlands with the studied region indicated in an orange rectangle. **(b)** Map with the areas sampled in 2013 for PLFA analyses (orange) and in 2014 for CLPP analyses (green). **(c)** Detailed map of one of the study areas indicating the location of the 24 sampling sites for this specific area. **(d)** Schematic representation of how samples were collected along the waterside of ditches.

In this study, we assessed the impacts of several drivers of soil microbial structuring and functional capacity at local and landscape scales in agricultural peatlands. We combine data from two sampling campaigns across agricultural peatlands in The Netherlands (**Figures 1a,b**), one that examined drivers of soil microbial community structure, as determined by phospholipid fatty acid (PLFA) analysis, and one that examined drivers of soil microbial community functional capacity, as estimated by community level physiological profiling (CLPP). These patchwork agricultural landscapes are highly heterogeneous, making them effective model systems for examining the effects of multiple environmental drivers on soil microbial communities (Vasseur et al., 2013). Comparison of samples within and between different polder areas made it possible to analyze drivers at two different spatial scales: local scale (sites within a sampled area) and landscape scale (differences between sampled polder areas).

## Materials and Methods

### Study Sites and Design

The field sites used in this study are situated in a peat area in the West of The Netherlands (**Figure 1a** and **Table 1**) and comprise nine ± 200 ha peatlands (Referred to as ‘sampling areas,’ **Figure 1b**, each comprising between 18 and 24 sampling sites). All areas are characterized by a mixture of intensive and extensively managed peatlands intersected by ditches, resulting in a mosaic of land uses. In the summer of 2013, a total of three agricultural peatland areas were sampled, and another six areas were sampled in the subsequent summer of 2014. In each area, between 18 and 24 transects were laid out on field margins (referred to as ‘sampling sites’ **Figure 1c**), as such edges account for 96% of the total vegetation species richness of a field (Kleijn et al., 2001). Each transect had a total length of 100 m, where the vegetation was surveyed for the sloping part of each transect up to the waterside (**Figure 1d**). Vegetation abundance was assessed using Tansley abundance classes (Tansley, 1946), which were subsequently converted into abundance percentages (**Supplementary Table S1**). To analyze soil physical-chemical properties and microbial community structure and functional capacity, five soil samples (10 cm deep) were taken in each transect, 20 m apart from each other and 20 cm from the waterside (**Figure 1d**). Soil samples were mixed per transect after removal of the vegetation layer, sieved (2 mm mesh) and stored at -80°C as one composite sample.

**Table 1:** Characteristics of the different study areas (ID) used for PLFA or CLPP.

	ID	Lon (°)	Lat (°)	Int. Agr. (%)	AES (%)	Nat. (%)	Org. N fertilizer (kg/ha/y)	Org. P fertilizer (kg/ha/y)	Inorg. N fertilizer (kg/ha/y)	Inorg. P fertilizer (kg/ha/y)	Water Area (%)	Peat (%)	Clay (%)
PLFA	*H*	4.75494	51.88792	50	24	26	167.2	62.7	36.9	11.7	13	95	5
	*I*	4.82294	51.86355	63	35	2	151.1	56.7	79.7	23.3	10	100	0
	*O*	4.89699	51.91930	90	9	1	169.3	66.5	86.2	26.3	10	93	7
CLPP	*Q*	4.53776	51.75151	5	9	86	160.9	63.9	0.0	0.0	7	61	39
	*R*	5.00921	52.25942	58	36	6	169.0	67.7	75.7	21.8	14	80	20
	*S*	5.03050	52.19264	44	0	56	134.7	68.7	19.8	6.5	12	83	17
	*T*	5.13003	52.27827	28	0	72	136.5	68.4	14.7	4.6	19	94	4
	*U*	4.77225	51.93984	65	9	26	167.5	62.8	36.0	11.6	13	94	6
	*Z*	4.78264	52.03339	3	8	90	92.0	48.6	0.0	0.0	16	100	0

*Land management of peatlands is given as the percentage of the management style to the total peatland. Estimated N and P application through fertilizers (organic and inorganic) and the total water area, peaty soil and clay soil percentages are also shown for each area.*

### Phospholipid Fatty Acid (PLFA) Analyses

Soil microbial community structure was determined by analyzing PLFA extracted from soil samples taken in the areas that were sampled in summer 2013 (three areas, 63 samples). PLFAs were extracted from 4 g of soil per composite sample using an adapted protocol, following White et al. (1979) and Frostegård and Bååth (1996). Lipid fractionation took place over prepacked Bond Elut SI solid phase extraction columns, after which lipid extracts were identified by gas chromatography (GC-FID, 7890A, Agilent Technologies, Wilmington, DE, United States). The (relative) abundance of fungi, Gram positive (G^+^) and Gram negative (G^-^) bacteria was characterized by the use of specific indicator PLFA biomarkers: fungi (18:2ω6), G^+^ bacteria (i14:0, i15:0, a15:0, i16:0, i17:0 and a17:0) and G^-^ bacteria (16:1ω7, cy17:0 and 18:1ω7). Total bacterial biomass was determined by taking the sum of all bacterial biomarkers, including the general biomarker 15:0. Abundance of each PLFA biomarker was expressed as nmol PLFA g^-1^ dry weight of soil.

### Community Level Physiological Profiling (CLPP)

Functional diversity of the soil microbial community was determined in soil samples originating from the areas that were sampled in summer 2014 (six areas, 144 samples) by the use of Biolog EcoPlates (Biolog, Hayward, CA, United States). These 96-well plates contain three replicate sets of 31 ecologically relevant C substrates, along with a tetrazolium redox dye (Insam, 1997). Microbial use of these substrates is reflected by color change in each of the wells, as the redox dye is reduced to tetrazolium violet (Pohland and Owen, 2009). Eco-plate wells were inoculated with diluted soil slurries (150 μl), obtained by mixing 0.5 g of soil with 49.5 mL of milli-Q water, shaken (200 rpm) for 30 min on an orbital shaker, and 10^-4^ diluted by serial dilution. Three technical replicates were included for each of the 144 soil samples. Eco-plates were incubated in the dark at 25°C, and color development was recorded as optical density (OD_590_, OD_750_) at the start and after 24, 48, 72, 96, 168, and 192 h, on a Biotek Synergy HT plate reader (Biotek Instruments, Winooski, VT, United States).

Conceptually, the function of Ecoplate-substrate utilization through time consists of four distinct phases (**Supplementary Figure S1A**). The substrate utilization function captures the signals of community respiration, but also that of the substrate consumed for community growth. For our purposes, we were interested only in the respiration of the originally sampled community. To remove the signal of reproduction from the data, we used a modified method of Brouns et al. (2016). The rationale behind this method is that by removing the exponential-growth signal from the exponential phase of the substrate-use function, only the substrate use of the initial community remains. The exponential phase is characterized by plentiful substrate where growth of organisms is not limited by its availability. By fitting a log-linear regression to the extracted exponential phase (**Supplementary Figure S1B**), we determined the initial community substrate use (y-intercept). In contrast to the existing methodology, we determined the phase of true exponential growth from the second derivative of a polygonal curve fit. By finding the inflection point, the point where the second derivative changes from positive to negative, the convex, true exponential, part of the curve is determined. We also accounted for the possible existence of a lag phase by removing non-positive values (i.e., zeroes). We calculated the classical Average Well Color Development (AWCD; Garland and Mills, 1991) and diversity metrics of substrate utilization (e.g., Gomez et al., 2006), substrate richness, the exponent of the Shannon diversity and Pielou’s evenness of the substrate utilization.

### Soil Chemical Analyses

Soil pH was measured after shaking a soil-water (1:2.5 w/v) suspension on an orbital shaker at 200 rpm for 2 h. Total C, N, and P analyses were performed on oven-dried (60°C, 96 h) and ground (1.0 mm, Retsch SM 100, Haan, Germany) soil samples. Total C and N were determined using an Elemental Analyser (Thermo Electron, Milan, Italy). Total P was determined according to the method of Murphy and Riley (1962). Soil samples were ashed at 550°C for 30 min, after which P was re-suspended by digestion with 2.5% (w/v) acid persulphate in an autoclave (30 min at 121°C). Total P was measured colorimetrically, on a continuous flow analyser (SEAL analytical, Abcoude, Netherlands). Soil moisture was determined as the percentage weight loss upon oven drying.

### Cartographic Information

Soil typological information, yearly fertilizer use and land management were extracted from geographical maps (Alterra, PAWN; Natuur op Kaart, Kadaster 2013/14, SNL, IPO 2013/14) using ArcGIS 10.1. With this information, we determined fertilizer use and N and P loadings per hectare. In determining artificial and organic fertilizer (manure), we assumed that farmers used the maximum amount of admissible fertilizer based on national legislation. Fields with specific nutrient management schemes, such as areas with natural grassland management generally use less artificial fertilizer due to a resting period where no fertilizer can be applied or due to legal restrictions on artificial fertilizer use. Also, manure application may be constrained due to the resting period or further limited to solid manure application for certain types of nature management. In designated natural grassland sites, neither artificial nor organic fertilizer application is allowed. We estimated inorganic and organic N and P loadings per hectare (ha) per year for fields in each polder (**Table 2** and **Supplementary Table S2**).

**Table 2:** Average and range [min;max] of local soil conditions of the different areas (H, I, O) used in soil community structure analyses (PLFA).

Variables	*H*	*I*	*O*
pH	4.51 [3.91; 5.4]	4.93 [3.87; 6.64]	4.34 [3.70; 5.10]
C (mg/g dry weight of soil)	208.62 [158.86; 273.75]	196.48 [92.9; 240.64]	177.96 [105.13; 237.67]
N (mg/g dry weight of soil)	14.95 [11.59; 18.74]	14.68 [6.8; 18.04]	12.89 [7.79; 16.57]
P (mg/g dry weight of soil)	1.42 [0.75; 2.06]	1.33 [0.84; 2.01]	2.69 [2.04; 4.22]
Moisture (%)	64.63 [40.23; 76.97]	61.88 [49.96; 74.72]	62.56 [37.78; 77.43]
Microbial biomass (nmol/g dry weight of soil)	20.01 [4.81; 64.93]	14.04 [4.93; 31.42]	11.77 [3.45; 38.35]
Fungal:Bacterial biomass ratio (-)	0.06 [0.03; 0.09]	0.06 [0.03; 0.09]	0.06 [0.03; 0.1]

### Data Analysis

All analyses were performed in R version 3.2.1 using the vegan, KernSmooth, MASernSmooth, MASS, PCNM, packfor and VennDiagram packages. In this study, we use two separate datasets on soil microbial communities (see **Supplementary Tables S1, S2**). One dataset contained data on soil microbial community structure (PLFA data), and another dataset contained data on soil microbial community functional capabilities (CLPP data). The PLFA data consisted of three areas containing 22, 18 and 23 sites, each. The CLPP data encompassed 6 distinct areas with 24 sites each. First, we examined general soil properties, biomass and PLFA and CLPP patterns in these datasets. We determined descriptive soil properties (soil C,N,P content, pH and moisture) and tested how microbial biomass changed along environmental gradients using generalized linear models with a gamma distribution and log link function to deal with deviations from normality. These models were run for the different proportions of biomass as calculated from the PLFA data as dependent variables and included all soil geochemical, as well as all land management-related variables and the polder area identity as explanatory variables. Second, to assess general patterns and clustering in polder areas we used a principal component analysis (PCA). We tested the importance of general drivers and the existence of polder level differences further using distance-based redundancy analysis models (dbRDA; Legendre and Anderson, 1999), where microbial community variation (in composition or functional capacity) between sites was expressed as an Odum’s percentage difference distance. Thus, large distances indicate very different sites and small distances indicate comparable sites in terms of community structure or functioning. We defined two spatial scale levels for this analysis, the local field level within polders and between polder areas at the regional level. At both scale levels, we carried out a variation partitioning analysis (Peres-Neto et al., 2006) using dbRDA. Prior to variation partitioning, a dbRDA analysis on the full data set was carried out. Next, all models were subjected to a forward selection procedure prior to variation partitioning (Blanchet et al., 2008). We subsequently assessed the importance of underlying variables in shaping the microbial community structure and functional capacity at the two scale levels by examining the explained variation (*R*^2^_adj_) of the selected variables in isolation.

### Local Scale: Model Definition

Differences in community composition or functional capacity at the local scale may result from differences in environmental quality between field edges. To analyze patterns at the local level, we applied the approach described by Declerck et al. (2011). Briefly, dummy-coded polder identifiers delineate the different study areas. These polder identifiers were used as covariates in the analysis to control for large-scale patterns in the data. By controlling for the polder identity, we could effectively study within polder patterns in community structure and function for multiple polders simultaneously. We distinguished four explanatory models at the polder level: a soil characteristics model (SOIL), a nutrient management model (NUT), a vegetation composition model (VEG) and a spatial model (SPACE).

The SPACE model was composed of Moran Eigenvector Map (MEM) variables that explain the spatial autocorrelation between sites in the landscape based on geographical distances (PCNM: Dray et al., 2006). By using staggered matrices of MEM eigenvectors (Declerck et al., 2011; Legendre et al., 2013), we described spatial autocorrelation among sites within polders. We only selected the eigenvectors with positive spatial correlations for analyses. The SPACE model represents small scale spatial patterning in community variation, large scale patterning already being excluded through the use of polder identity as a covariate. Our SOIL model consisted of variables describing the quality of the soil (pH, C, N, and P content, C:N, C:N, and N:P ratio, moisture), morphometric characteristics (bank angle, bank width) and soil typology. Our NUT model consisted of loadings of organic and inorganic N and P applied to the field along with dummy coded variables of the occurring nutrient management schemes (**Supplementary Table S4**). Our VEG model consisted of a staggered matrix of the principal axes of a principal coordinate analysis (PCoA) per polder. Because many axes of a PCoA explained little to no variation, we selected for relevant axes based on a broken stick model of explained variation, with all axes before the break point being selected. The resulting axes were arranged into a staggered matrix (Legendre et al., 2013) with the goal of only representing within polder differences. All models were subjected to forward selection based on a double stopping criterion (*R*^2^_adj_ and a > 0.05) and tested for significance using 99,999 permutations constrained within polder identity levels.

### Regional Scale: Model Definition

Differences in community composition or functioning at the landscape scale may result from differences in environmental quality between polders. Environmental gradients existing at the spatial grain of the landscape may be markedly different from those at the field level. Hence, an examination of these gradients irrespective of the variation explained within polders is appropriate. To this end, we used an approach where we first constructed a statistical model explaining community variation by dummy-coded polder identity variables (Polder model). By taking the predicted values of this polder model, we obtained a matrix of community variation present between polder landscapes only. We used this matrix as our response matrix in subsequent analyses of drivers of community variation between polders, allowing us to make models that only explain community variation encompassed by the polder model. Here, we constructed three explanatory models at the level of the landscape: a SOIL, a nutrient loading model (NUT) and a vegetation composition model (VEG). For the sake of interpretation, the explained variation of the models was rescaled to the total community variation captured by the polder model.

Our SOIL and NUT models consisted of the same variables as those used at the local scale. Our VEG model was created by transformation of an Odum’s percentage dissimilarity matrix of the vegetation composition of all field edges within the respective data set by means of a principal coordinate analysis (PCoA). For the model explaining community variation, we made use of the resulting PCoA axes. This approach differs from the one used at the local scale in that we did not use PCoA axes per polder, but rather examined variation across all polders. As for the local model, we selected for the relevant PCoA axes based on a broken stick model of explained variation, with all axes before the break point being selected. The uniquely explaining part of the variation of the polder model, the part not explained by environmental drivers, may be interpreted as spatial patterning at the landscape level that is not directly related to the measured environment. A formal permutation test is not viable with the limited number of different polders. Hence, forward selection was carried out using the increase in *R*^2^_adj_ as the only criterion. Additionally, when models were found to be non-significant in explaining patterns in the full data, irrespective of spatial scale, we disregarded the model in this analysis.

## Results

### Soil Chemical Properties and Microbial Biomass Patterns

**FIGURE 2:**
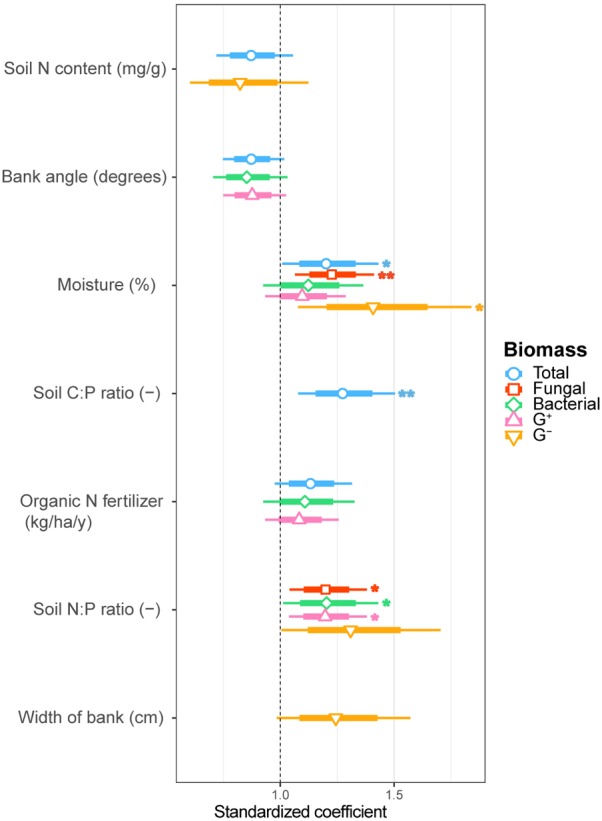
Estimated standardized coefficients for AIC selected generalized linear models of total, fungal, bacterial, G^+^ bacterial and G^-^ bacterial biomass explained by environmental drivers. Asterisks indicate significant coefficients (^∗∗∗^*p* < 0.001; ^∗∗^*p* < 0.01; ^∗^*p* < 0.05.) with a coefficient above 1 being a positive and below 1 a negative correlation with the biomass component.

We examined soil chemical properties of our two data sets by calculating mean and spread of the soil chemical properties for all sample areas (**Tables 2, 3**). The sample areas used in the assessment of soil community structure analyses, showed a wide range in nutrient content (**Table 2**) as well as in soil moisture levels across the different sample areas. Areas also showed a wide range in microbial biomass (**Table 2**), which persisted across different groups (**Supplementary Figure S2**). The microbial biomass increased with decreasing relative soil P-content (measured as molar C:P ratio and N:P) and was positively correlated to soil moisture levels (**Figure 2** and **Supplementary Table S3**). The range of soil properties (nutrient content and soil moisture) was even greater for the samples examined by CLPP, though average soil chemical properties were within the same general range (**Table 3**). Substrate utilization was largely comparable between areas, though varied considerably within areas (**Table 3** and **Supplementary Figures S3, S4**).

**Table 3:** Average and range [min;max] of local soil conditions of the different sampling areas (Q,R,S,T,U,Z) used in soil community functioning analyses (CLPP).

Variables	*Q*	*R*	*S*	*T*	*U*	*Z*
pH	6.67 [4.74; 7.84]	5.44 [4.76; 6.4]	5.34 [4.23; 6.62]	5.30 [4.06; 6.24]	6.03 [5.46; 7.43]	5.55 [4.63; 6.03]
C (mg/g dry weight of soil)	121.17 [59.6; 273.9]	228.0 [152.2; 284.1]	255.4 [167.7; 298.3]	156.51 [47.9; 410.49]	220.6 [27.2; 315.9]	268.9 [159.0; 329.0]
N (mg/g dry weight of soil)	8.23 [4.45; 17.46]	16.6 [12.12; 20.53]	19.55 [13.1; 22.18]	10.05 [3.66; 22.67]	16.54 [1.64; 23.06]	21.31 [14.29; 25.48]
P (mg/g dry weight of soil)	1.00 [0.39; 1.77]	1.28 [0.94; 2.07]	1.59 [1.21; 2.07]	0.91 [0.42; 1.28]	1.5 [0.75; 2]	0.72 [0.49; 1.15]
Moisture (%)	50.21 [14.83; 69.29]	68.62 [56.12; 87.12]	68.44 [55.4; 76.8]	58.13 [34.82; 80.83]	65.37 [31.54; 78.54]	72.33 [49.52; 79.41]
Substrate utilization (h^-1^)	0.0171 [0.006; 0.179]	0.0103 [0.005; 0.023]	0.010 [0.003; 0.025]	0.0085 [0.003; 0.020]	0.0102 [0.006; 0.018]	0.0107 [0.0038; 0.027]
AWCD	0.73 [0.55; 0.89]	0.64 [0.42; 0.9]	0.73 [0.48; 0.89]	0.5 [0.2; 0.78]	0.65 [0.47; 0.88]	0.64 [0.24; 0.99]
Substrate richness	27 [22; 31]	24.38 [16; 31]	26.5 [23; 31]	24.79 [19; 30]	25.62 [21; 30]	25.75 [19; 30]
Substrate diversity (Shannon)	22.78 [17.59; 25.54]	20.62 [13.48; 25.21]	22.56 [19.2; 26.72]	19.77 [14.23; 25.53]	21.18 [17.26; 27.82]	20.91 [14.45; 26.77]
Substrate evenness	0.71 [0.55; 0.8]	0.64 [0.42; 0.79]	0.7 [0.6; 0.84]	0.62 [0.44; 0.8]	0.66 [0.54; 0.87]	0.65 [0.45; 0.84]

### Drivers of Soil Microbial Community Structure at Different Spatial Scales

**FIGURE 3:**
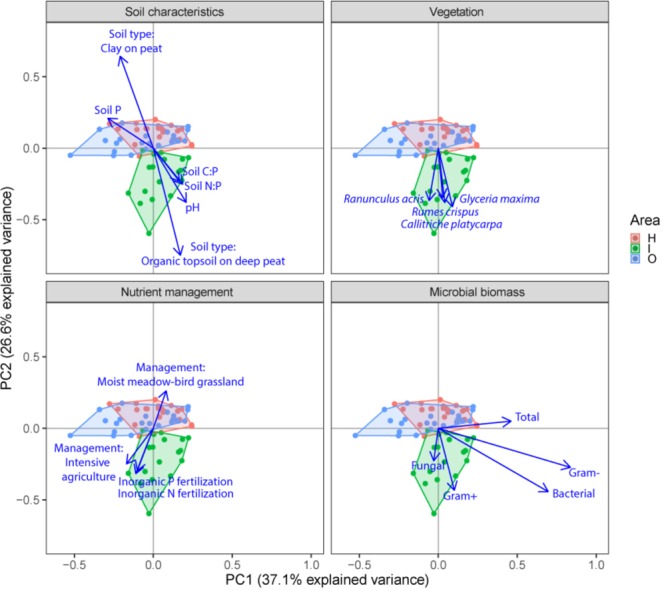
Principal component analysis (PCA) plots of soil microbial structure data (PLFA) for the three different groups of drivers (Soil characteristics, Nutrient management and Vegetation) and shifts in total microbial biomass, fungi, gram-positive bacteria, gram-negative bacteria and total bacteria. Arrows are projected variables showing variables with the highest squared correlation coefficients. Different colors indicate the different sampled areas (H, I, O).

**FIGURE 4:**
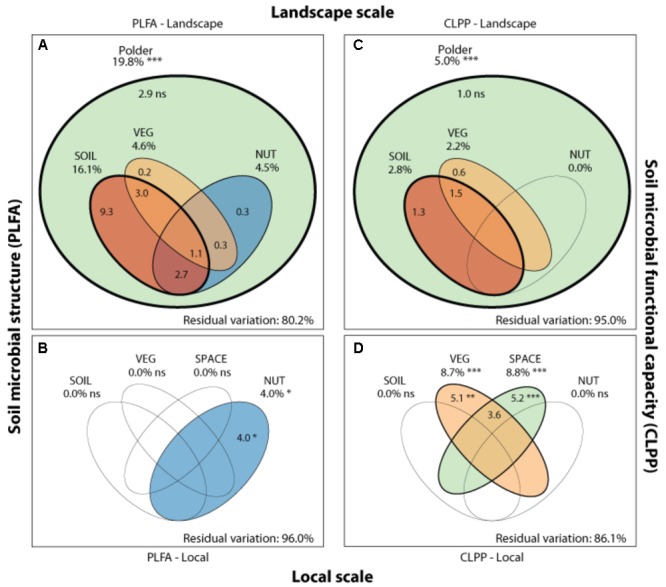
Drivers of microbial community structure and functioning on local and landscape scale. Venn diagrams showing the variation partitioning of different statistical dbRDA models: a soil characteristics model (SOIL), a nutrient management model (NUT) and a vegetation composition model (VEG) and a spatial model (SPACE). These models are used to explain soil microbial structure (PLFA) at the landscape **(A)** and local **(B)**, and functioning (CLPP) at the landscape **(C)** and local scale **(D)** by different drivers. Stars indicate significance and numbers express the adjusted *R*^2^ (%) of the model partitions.

Soil microbial community structure was examined by PLFA fingerprinting. In a first examination of PCA results (**Figure 3**), we found clear differences between the different polder areas examined. A dbRDA of the PLFA data revealed that 19.8% of the community variation could be explained by differences between polders (**Figure 4A** and **Supplementary Table S4C**) and a mere 4.0% could be explained within polders (**Figure 4B** and **Supplementary Table S4A**). Nonetheless, we were able to identify consistent, significant gradients explaining community structure (**Supplementary Table S4**). At the local scale (**Figure 4B**), only the NUT model proved to explain a significant portion of community variation (*R*^2^_adj_ = 4.0%, *P* < 0.01). This leaves large parts of the total variation explained at the level of the full dataset unaccounted for (**Supplementary Table S5**). A part of this community variation was found to be explained at the landscape scale instead (**Figure 4A**) by means of the SOIL, NUT and VEG models. Only 2.9% (ns) of the variation was unique to the polder model, and not captured by one of the other models (**Figure 4A** and **Supplementary Table S4A**). The SOIL model was the most important explaining environmental component (*R*^2^_adj_ = 16.1%), encompassing large parts of the explained variation of the NUT (2.7% + 1.1% = 3.8%) and VEG model (3.0% + 1.1% = 4.1%).

We ranked variables underlying the main drivers identified in the variation partitioning in terms of their importance (**Table 4**). At the landscape scale, PLFA structuring responded most strongly to nutrient-related variables, the soil P content (10.3%), inorganic N fertilization (3.1%), soil N content (1.7%), organic P (1.0%) and N fertilization (0.6%). In addition to nutrient-related parameters, soil type (7.8%), the presence of nature management schemes (2.6%), agri-environmental schemes (0.2%), and the resident vegetation composition (4.6%) were shown to be important variables in explaining landscape level community structure. At the local scale, less of the variation in PLFA data was explained, with organic P fertilization being the most pronounced driver (5.7%) of microbial community structure.

**Table 4:** Importance of variables underlying soil microbial community structure (PLFA) at both scale levels (local and landscape).

		Explained variation^∗^	
Model	Variable	Local	Landscape
Soil characteristics (SOIL)	Soil P content	–	10.3
	Soil type: Organic top soil on deep peat layer	–	7.8
	Soil N content	–	1.7
Nutrient management (NUT)	Organic P fertilization	5.7	1.0
	Management: Nature – Moist meadow-bird grassland	0.8	2.6
	Inorganic N fertilization	–	3.1
	Organic N fertilization	–	0.6
	Management: AES - Meadow-bird nest protection	–	0.2
Spatial patterns (SPACE)	ns	–	–
Vegetation composition (VEG)	Vegetation composition	–	4.6

*^∗^Explained variation of each variable is given as R2 (%) of the variable. ^-^Variable was not selected in the forward selection of the specific model.*

### Drivers of Microbial Community Functional Capacity at Different Spatial Scales

**FIGURE 5:**
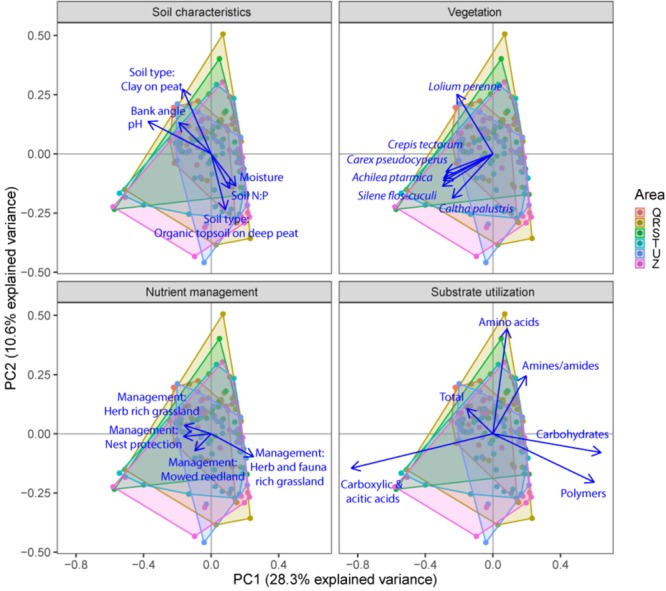
Principal component analysis (PCA) plots of soil microbial functional capacity data (CLPP) for the three different groups of drivers (Soil characteristics, Nutrient management, and Vegetation), with projections of the shifts in the utilization of specific substrate types. Arrows are projected variables showing factors variables with the highest squared correlation coefficients. Different colors indicate the different sampled areas (see Materials and Methods).

Community level physiological profiling was used as a proxy for the functional capacity of the microbial community. In a first examination of PCA results (**Figure 5**), we found a strong overlap between sites of the different polder areas under examination. This was also reflected in RDA analyses of the data, with only 5.0% of the total variation in community functional capacity being explained by the polder model (**Figure 4C**). Despite this small part of the variation being explained, we did find that part of the CLPP variation between landscapes was associated with soil characteristics (*R*^2^_adj_ = 2.8%) and vegetation (*R*^2^_adj_ = 2.2%) (**Figure 4C**). Nutrient management was found to be non-significant in explaining patterns in the full dataset (**Table 1**), and it therefore did not explain any of the variation encompassed by differences between polders. At a local scale (**Figure 4D**), we could explain a larger part of the variation (13.9%), which was attributed to the vegetation composition model (*R*^2^_adj_ = 8.7%, *P* < 0.001) and spatial patterns in community functional capacity (*R*^2^_adj_ = 8.8%, *P* < 0.001).

We identified the primary driving variables related to soil microbial community functional capacity (**Table 5**). At a the landscape scale, soil pH (1.1%) and soil type (0.9%) and soil P ratios (soil N:P: 0.7% and soil C:P: 0.6%) were found to be most explaining for the variation in functional capacity. The local scale was explained by the vegetation community and a spatial MEM model based on geographical distance between field edges. The latter showed that most patterns were described by the highest order MEM variable (7.7%), indicative of a relatively coarse spatial patterning of community functioning.

**Table 5:** Importance of variables underlying soil microbial functional capacity (CLPP) at both scale levels (local and landscape).

		Explained variation^∗^	
Model	Variable	Local	Landscape
Soil characteristics (SOIL)	Soil pH	–	1.1
	Soil type: Sand	–	0.9
	Soil N:P	–	0.7
	Bank angle	–	0.6
	Soil C:P	–	0.6
	Soil type: Clay on peat	–	0.6
	Soil N content	–	0.6
	Soil C:N	–	0.3
Nutrient management (NUT)	ns	–	–
Spatial patterns (SPACE)†	MEM1	7.7	–
	MEM2	3.1	–
	MEM3	1.4	–
Vegetation composition (VEG)	Vegetation composition	8.7	2.2

*^∗^Explained variation of each variable is given as R2 (%) of the variable. ^-^Variable was not selected in the forward selection of the specific model. ^†^Spatial patterns model is composed of Moran Eigenvector Map (MEM) variables based on geographical distance as per Dray et al. (2006). Variables of increasing order indicated decreasing scale of spatial patterning.*

### Comparing Community Structure and Functional Capacity

Comparing the two datasets, the two analyses of community variation yielded highly disparate results with respect to the scale at which different environmental factors could explain variation in the data (**Figure 4**). Community structure data (PLFA) was associated with environmental factors between different polders, i.e., at a large landscape scale (**Figure 4** and **Supplementary Table S4**). In contrast, functional data was poorly explained at this scale; rather environmental variation within polders offered the greatest level of explanatory power (**Figure 4**). Despite the difference in total explained variation, at the landscape scale the general partitioning and relative weight of the drivers was comparable for both PLFA structure and CLPP (**Figure 4** and **Supplementary Table S4**). Both microbial community properties were most explained by the SOIL model with a small contribution of the variance being explained by VEG. Moreover, variation was highly collinear between the different models. On a local scale, patterns were markedly different between community structure and functional capacity.

## Discussion

Understanding the drivers of soil microbial processes at relevant scales can help to improve management of agricultural peatlands to protect and improve desired ecosystem functioning. Through our analyses, we have examined the driving forces of microbial community structure and functioning in field margins along agricultural banks at two different scale levels; within polders (local) and between polders (landscape). We found local and landscape scale drivers to be distinct at different scale levels. The underlying variables were found to be largely different as well. This implies that the spatial scale of soil microbial studies is important when talking about driving forces of soil microbial community structure and functioning, enforcing the idea that the scale of soil management and the scale of study of soil microbial structure and functioning need to be well aligned.

### Local Functioning, Landscape Structuring

While somewhat anecdotal due to the separate collection of the datasets, we showed that soil microbial community structure (PLFA) was more strongly regulated at the landscape scale, while functional capacity (CLPP) was more strongly driven at the local scale. Explained variation, while not being exceptionally high (15–20%), was comparable to other studies using similar multivariate community analysis approaches (Van der Gucht et al., 2007; Sayer et al., 2017). Future studies could consider integrated methods that address both structure and functioning conjointly (e.g., ^13^C PLFA, Yao et al., 2015). The inclusion of additional environmental drivers, such as specific fractions of bio-available nutrient pools, would potentially have increased the amount of explained variation. Across polder regions, e.g., at the landscape scale, the results indicate a driving role for soil characteristics, with vegetation being largely collinear with soil characteristics (for similar findings see: Kuramae et al., 2010). We therefore conclude that, with respect to soil microbial structure, differences in vegetation and nutrient management between polders are well reflected in the soil characteristics. Local microbial structure could only be led back to the applied nutrient management of the field and explained little variation. In contrast, variation in community functional capacity could be explained better by vegetation composition and spatial patterns at the local scale, with both explaining distinctly different parts of the community variation. The overlap in drivers at the landscape scale is likely due to the fact that the studied areas vary in land-use, land-history and management, which leads to landscape-scale vegetation and nutrient availability patterns that leave clear imprints in the soil. Locally, the small-scale heterogeneity of fields becomes more important in driving the specific microbial function. This mismatch in scale between structure and function has been described previously for specific microbes and their functions (Veraart et al., 2017).

### Drivers of Soil Microbial Community Structuring and Functional Capacity

Drivers of community variation may differ strongly with scale (Yergeau et al., 2010; Prober et al., 2015), and our analyses support this premise. At both scale levels, community structure was driven by nutrient management. The latter result is in agreement with previous research (O’Donnell et al., 2001; O’Brien et al., 2016) that has shown the importance of fertilization regimes for soil microbial communities. In turn, the supply and manner in which nutrients are added can have direct consequences for ecosystem functions such as nutrient retention and plant uptake (Heijboer et al., 2016). We, however, did find clear differences in underlying drivers of nutrient management of the within and between polder scales, with organic P loading and inorganic N loading being most important. This highlights the importance of identifying underlying drivers (Martiny et al., 2011). By focusing on a single scale level, important drivers may be overlooked and incorrect conclusions may arise, potentially leading to mismanagement of the agricultural landscape.

Our conclusions regarding landscape scale patterns are complicated by the lack of extensive replication at the landscape level, making formal testing of the drivers encapsulated within the polder model problematic. While we acknowledge these limitations within our study, our results are strengthened by the strong significant patterns found in tests of the entire data set (**Supplementary Table S5**). As large parts of the total variation that can be explained by our models remain unexplained at the local scale (e.g., **Figure 4**), it is reasonable to assume that this variation may be explained at the landscape scale.

A surprising similarity in soil characteristic drivers of soil microbial community structure and functioning can be found for nutrient-related drivers (soil N:P ratio, soil P and N content). Specifically, soil N content was found as the only variable that was important in determining landscape scale community structure, as well as the community functional capacity. Additionally, for community structure, specifically P-related processes were important drivers at a local (organic P fertilization) and at a landscape scale (soil P content, organic P fertilization). Soil nutrient content and the relative P availability compared to other nutrients were also primary drivers of microbial biomass. In existing literature, little attention has been paid to the effects of P on peatland microbial communities and functioning (Lin et al., 2014; Veraart et al., 2015). Our results suggest that these effects of P enrichment on peatland microbial communities deserve additional consideration.

The relevance of the resident vegetation community for local microbial functional capacity, but not local microbial structure, is a noteworthy result. This could be caused by the study design in which we compare different polder areas with slightly different plant communities. An ecological explanation for this may be found in the stimulating role of plant presence and diversity on the function of soil microbes by (e.g., Zak et al., 2003). Furthermore, a well-developed, species-rich riparian zone will influence water and nutrient retention (Hefting et al., 2005) and thereby microbial functioning (Korol et al., 2016). This development of a riparian zone depends strongly on local disturbance by mowing and cattle grazing. We did not directly quantify these factors, although they should in part be represented in the nutrient and land management schemes. However, within these schemes, there is room for variation in grazing and mowing regimes at the digression of the land manager. As land managers tend to own different nearby fields within a landscape, this variation in mowing a grazing is likely to be spatially structured. Our results, where vegetation and spatial structure explain local functional capacity, may thus be (partially) explained by these unmeasured differences in management regimes.

We found evidence for spatial patterns that could not be explained by any of the measured environmental drivers at the level of the local functional capacity (uniquely explained variation of the SPACE model), which may represent a possible signal of dispersal limitation (Dray et al., 2006). While dispersal-limitation has been shown to be plausible within microbial communities (Evans et al., 2017; Langenheder et al., 2017), it is rarely a significant driver of microbial community structure (Martiny et al., 2011; O’Brien et al., 2016). Hence, our observed spatial patterns are likely to be caused by spatially structured environmental variables (e.g., light climate, soil redox conditions, readily available nutrient fractions, available substrates) that were not taken into account in this study (Martiny et al., 2006; Yao et al., 2011).

### Management of Soil Microbial Communities in Peatlands: An Integrative Approach

Our results suggest that microbial function is regulated by multiple different drivers that are distinct from those driving soil microbial structure, and that these drivers act at different spatial scales. This complicates the task of managing agricultural peatlands for desired ecological functioning. The traditional view maintains that environmental drivers influence community structure and that this structure in turn influences community functioning (Allison and Martiny, 2008). However, this paradigm has been proven to be insufficient to explain microbial functional patterns in nature (Strickland et al., 2009; Weedon et al., 2017). Microbial functions have been shown to change independently of microbial community structure (Tian et al., 2016; Weedon et al., 2017) and respond to different variables than structure (Boeddinghaus et al., 2015). However, disregarding community structure entirely and solely focusing on functioning is also clearly inappropriate, as microbial community structure serves as a constraint on the realized functioning of the community and the ecosystem as a whole (Pérez-Valera et al., 2015; Heijboer et al., 2016).

**FIGURE 6:**
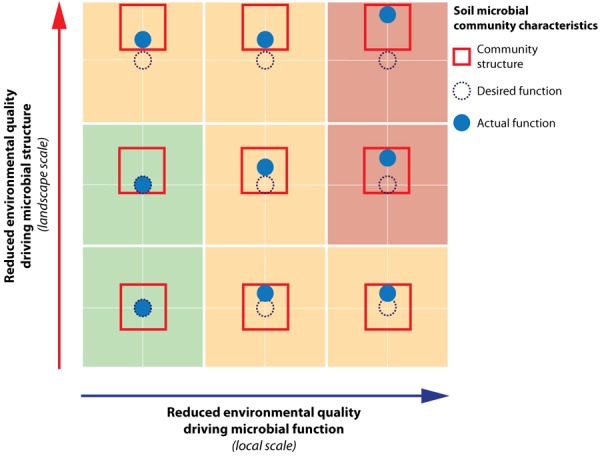
Schematic representation of the effects of reduced environmental quality on soil microbial community structure and functioning. This conceptual figure illustrates how reduced environmental quality of drivers relevant for functional capacity will directly lead to shifts of soil microbial functioning away from its desired function. Reduced environmental quality relevant for microbial structural composition will cause shifts in the soil microbial community structure box. This can ultimately also result in a shift in soil microbial community function through its constraint on microbial function. Within the context of the current study, the environmental drivers of microbial functioning were found to be manifest at the local scale, while the drivers shaping structure operated at the landscape scale.

We argue that for effective management of desired functioning to optimize the different societal benefits obtained from the landscape, both soil microbial structure and functioning need to be considered. Based on our study, environmental quality changes relevant for soil microbial functional capacity were most pronounced at the local scale. As local environmental quality shifts, this may lead to a direct shift in realized functioning away from the desired function (**Figure 6**, horizontal axis). However, the magnitude of this shift may be limited by the community structure, which constraints the extent of the shift in function (e.g., compare **Figure 6**, central-right and bottom-right, respectively unconstrained vs. constrained situation). Changes in environmental drivers governing structure (**Figure 6**, vertical axis) were primarily found to manifest themselves at the level of the landscape within the context of this study. A change in environment at the landscape level may hamper realization of the desired function by constraining the realized function negatively as well (e.g., **Figure 6**, top-left). Hence, a thorough understanding of the community structure and its potential to facilitate the desired function is an imperative first step in soil microbial management, followed by optimization of the conditions directly driving required soil microbial functioning. Throughout this process, the spatial scale at which microbial structure and functioning responds to these changes needs to be taken into account. Landscape measures, such as water level fluctuations and spatial planning set the constraints for the potential functioning (i.e., structure), and once this stage has been set, local management options such as mowing and fertilization regimes are decisive in determining if the desired functioning can be achieved.

## Conclusion

Our study showed that soil microbial communities of agricultural peatlands are driven by different factors at distinct, management-relevant spatial scales. Furthermore, our study provides a first indication that soil community structure and function do not necessarily respond to the same factors, or at the same spatial scales. We argue that it is important to take both these soil microbial community characteristics (structure and function) into account for management of these important ecosystems. Based on this study, we suggest optimizing management of microbial ecosystem functioning in peatlands by first focusing on landscape restoration, followed by suitable local scale management optimization. This is directly relation to recent initiatives such as the 4‰ initiative for increasing soil C storage in agricultural areas (Le Foll, 2015) and efforts to optimize long-term biogeochemical functioning of agricultural peatlands.

## Author Contributions

ST and AH conceived the conceptual idea of the study, carried out chemical, and microbial analyses respectively. ST and SD designed the field sampling design. AH designed the lab sampling protocols. ST carried out the field sampling. ST and AH carried out data analysis with help from SD. ST, AH, and AV wrote a first draft of the paper after useful discussions with SD and GK. All the authors contributed substantially to the final version of the manuscript for submission.

## Conflict of Interest Statement

The authors declare that the research was conducted in the absence of any commercial or financial relationships that could be construed as a potential conflict of interest.
