# Supplementary material for: Local Functioning, Landscape Structuring: Drivers of Soil Microbial Community Structure and Function in Peatlands
Source: Front Microbiol. 2018 Sep 3;9:2060. doi: 10.3389/fmicb.2018.02060 (PMC6129579; doi:10.3389/fmicb.2018.02060)
Supplement: Supplementary file 1 [file Table_1.docx]

Supplementary Information


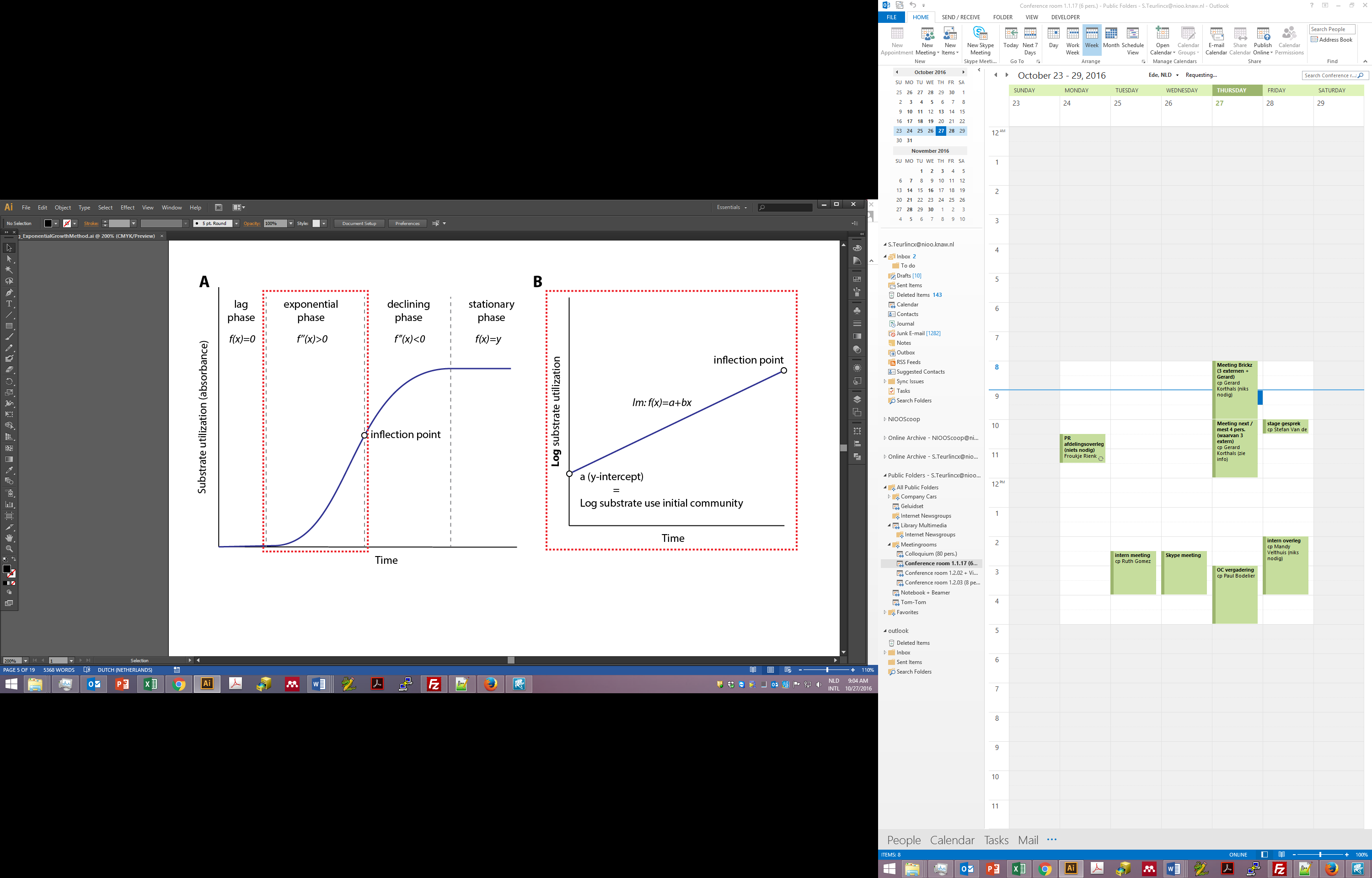


**FIG S1 | Schematization of the steps to calculate substrate use by the initial microbial community (CLPP).** A) Conceptually, the function of substrate utilisation through time consists of four distinct phases. The lag phase in which no measurable amount of substrate is utilized, the exponential phase in which the function is convex (f’’(x)>0), the declining phase in which the function is concave and a stationary phase in which the substrate is depleted. B) By extracting the exponential phase of the function (delimited by positive y values and the inflection point) and fitting a log-linear model, the y-intercept is found. This yields the substrate use without growth, the substrate utilization of the initial community.


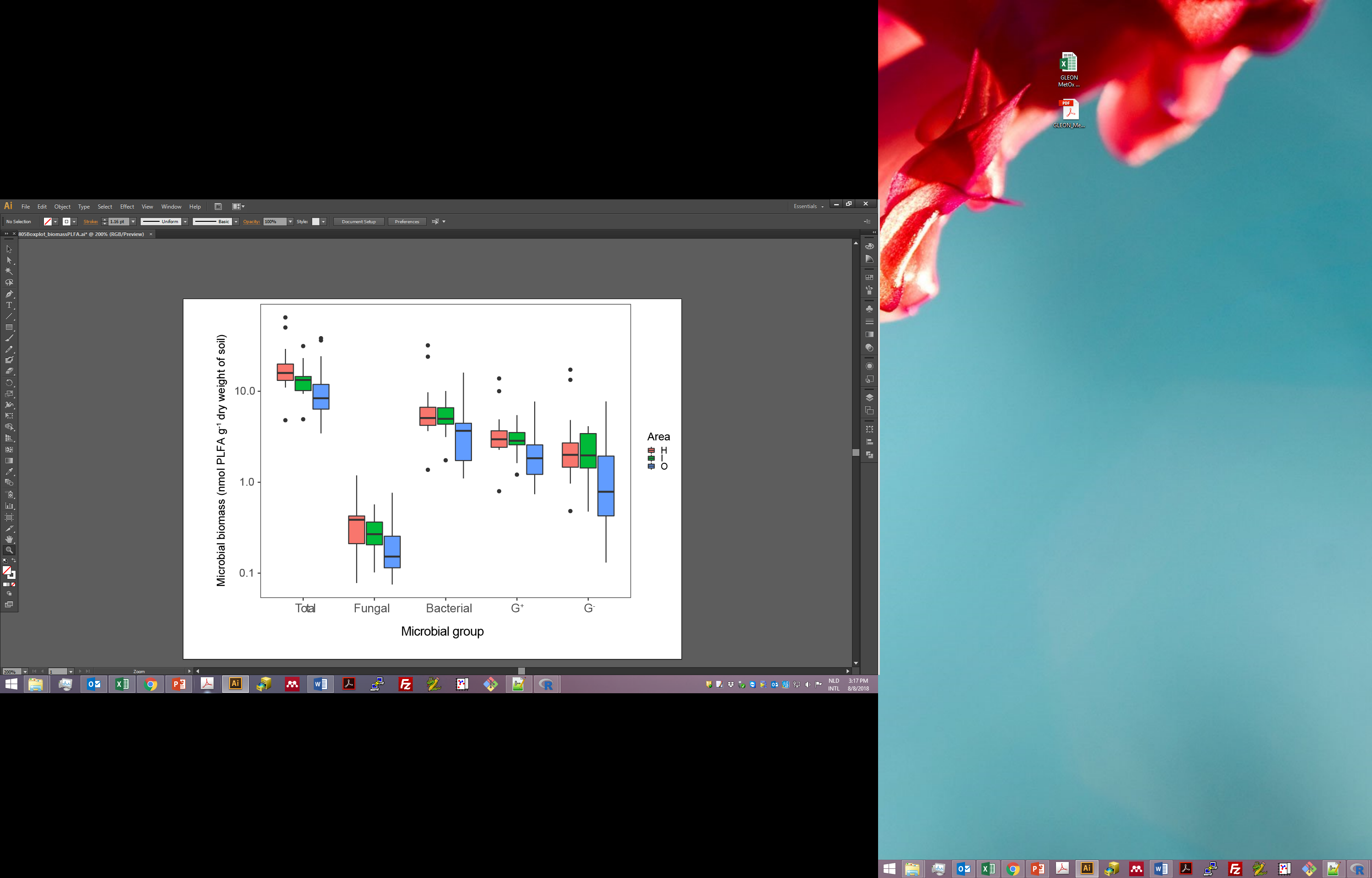


**FIG S2 | Boxplots of the microbial biomass of different sampling areas given for the different microbial groups as calculated from PLFA fingerprinting data.**


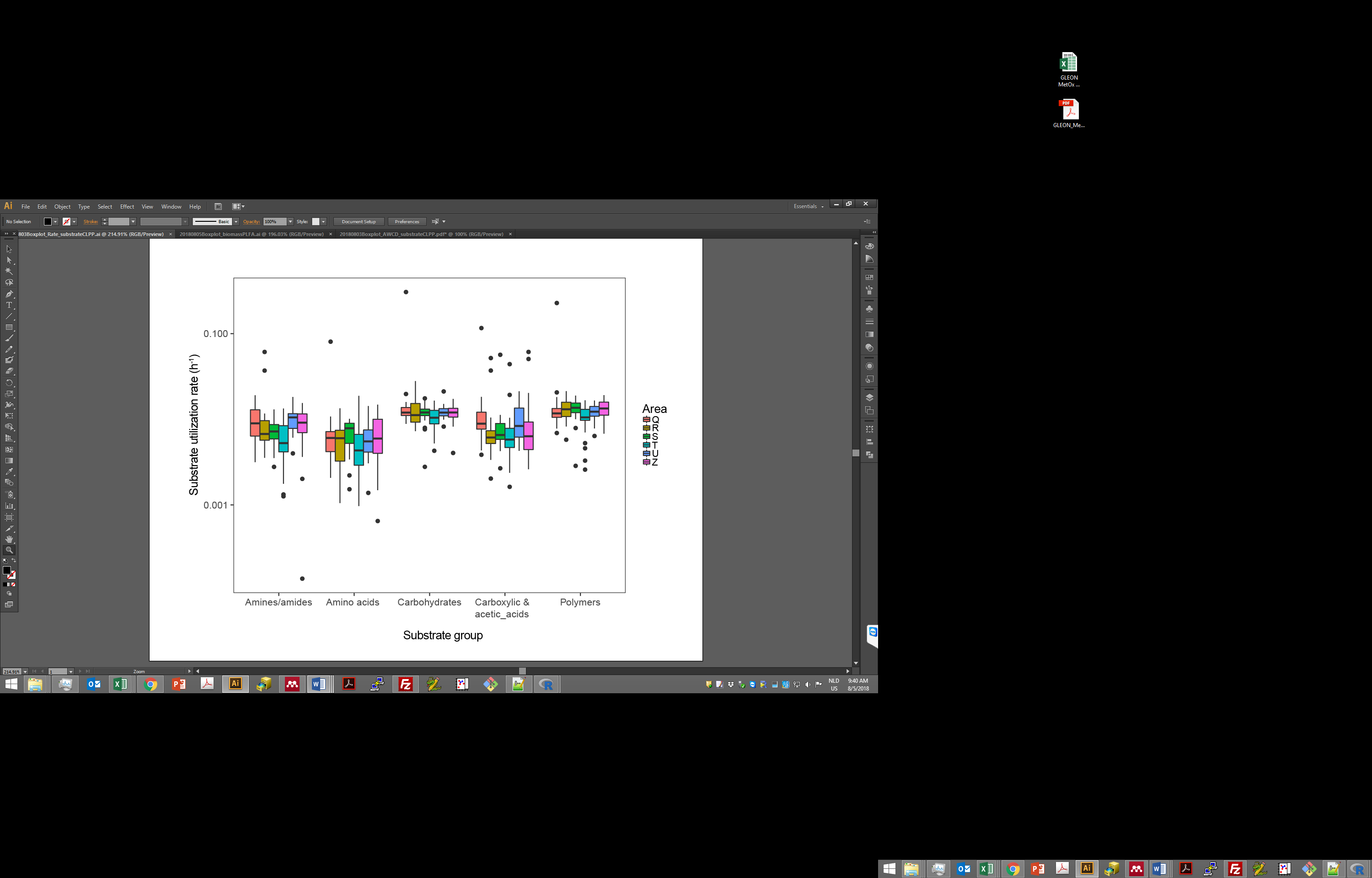
 **FIG S3 | Boxplots of the substrate utilization rate of different areas given for the groups of the substrate types as present in the Biolog Ecoplate.**


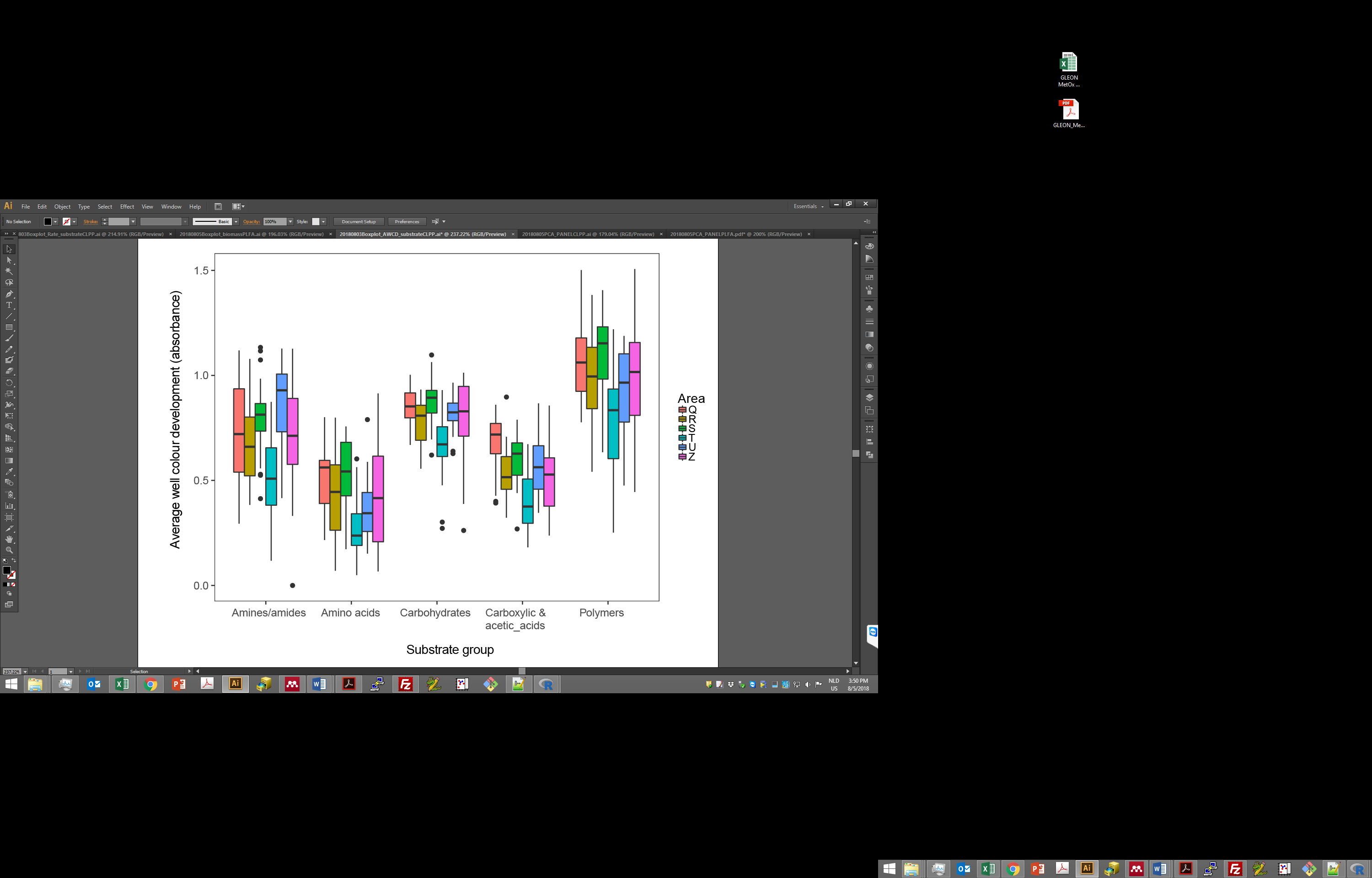


**FIG S4 | Boxplots of the average well colour development (AWCD) of different areas given for the groups of the substrate types as present in the Biolog Ecoplate.**

**Table S1 | Conversion of Tansley abundance classes to numeric classes and percentage cover.**

| Tansley score | Tansley numeric score | Cover  (%) |
| --- | --- | --- |
| *d* | 11 | 60 |
| *cd* | 10 | 40 |
| *ld* | 9 | 22 |
| *a* | 8 | 15 |
| *la* | 7 | 9 |
| *f* | 6 | 8 |
| *lf* | 5 | 3 |
| *o* | 4 | 2 |
| *lo* | 3 | 1 |
| *r* | 2 | 0.5 |
| *s* | 1 | 0.1 |

**Table S2** | **Nutrient-management schemes**. Description of the different nutrient-management schemes and their respective N and P fertilization limits based on resting periods and legislation.

| Nutrient management practice | | N_organic_ limit | N_inorganic_ limit | P limit | Times mown (per year) | First mowing day |
| --- | --- | --- | --- | --- | --- | --- |
| **Agri-environmental schemes** | *Meadow-bird grassland to June 1* | 170.0 | 18.8 | 67.7 | 4.0 | 153 |
|  | *Meadow-bird grassland to June 15* | 109.2 | 0.0 | 39.1 | 3.0 | 167 |
|  | *Meadow-bird grassland, pre-grazed* | 170.0 | 51.0 | 79.2 | 3.0 | 167 |
|  | *Meadow-bird nest protection* | 170.0 | 58.6 | 82.0 | 4.0 | 134 |
|  | *Herb-rich meadow-bird grassland* | 161.3 | 0.0 | 57.8 | 2.0 | 174 |
|  |  |  |  |  |  |  |
| **Nature** | *Swamp* | 0.0 | 0.0 | 0.0 | 0.0 | - |
|  | *Mowed reed land* | 0.0 | 0.0 | 0.0 | 1.0 | 106 |
|  | *Moist hay meadow* | 170.0 | 0.0 | 70.0 | 1.0 | 153 |
|  | *Herb and fauna rich grassland* | 170.0 | 0.0 | 70.0 | 1.0 | 153 |
|  | *Moist meadow-bird grassland* | 170.0 | 0.0 | 70.0 | 2.0 | 153 |
|  |  |  |  |  |  |  |
| **Intensive agriculture** | *Intensive agriculture* | 170.0 | 95.0 | 95.0 | 5.0 | 134 |

**Table S3 |** Summary of regression analysis of microbial biomass components against environmental variables. Generalized linear models with a gamma distribution were selected using an AIC selection. Mean coefficients, 95% confidence limits and test statistics are given for each variable (where selected) with stars indicating significance: *** p < 0.001; ** p < 0.01; * p < 0.05.

|  | Total | Fungal | Bacterial | Gram+ | Gram- |
| --- | --- | --- | --- | --- | --- |
| **Soil N content (mg/g)** | -0.14 [-0.33; 0.05]  (-1.40) |  |  |  | -0.19 [-0.50; 0.12] (-1.23) |
| **Bank angle (degrees)** | -0.14 [-0.29; 0.02]  (-1.74) |  | -0.16 [-0.35; 0.03]  (-1.64) | -0.13 [-0.29; 0.03] (-1.64) |  |
| **Moisture (%)** | 0.18 * [0.01; 0.36] (2.05) | 0.20 ** [0.06; 0.35] (2.83) | 0.12 [-0.08; 0.31] (1.17) | 0.09 [-0.07; 0.25] (1.12) | 0.34 * [0.07; 0.61] (2.51) |
| **Soil C:P ratio** | 0.24 ** [0.08; 0.41] (2.85) |  |  |  |  |
| **Organic N fertilizer (kg/ha/y)** | 0.12 [-0.02; 0.27] (1.63) |  | 0.10 [-0.08; 0.28] (1.12) | 0.08 [-0.07; 0.23] (1.06) |  |
| **Soil N:P ratio** |  | 0.18 * [0.04; 0.32] (2.51) | 0.19 * [0.01; 0.36] (2.10) | 0.18 * [0.04; 0.32] (2.48) | 0.27 [0.01; 0.53] (2.00) |
| **Width of bank (cm)** |  |  |  |  | 0.22 [-0.02; 0.45] (1.83) |
|  |  |  |  |  |  |
| **p-value** | 0.0001 | 0.0001 | 0.0016 | 0.0011 | 0.0052 |
| **deviance** | 14.74 | 17.31 | 22.37 | 15.86 | 42.00 |
| **pseudo R2** | 0.36 | 0.19 | 0.28 | 0.28 | 0.25 |
| **df.residual** | 57.00 | 60.00 | 58.00 | 58.00 | 58.00 |
|  |  |  |  |  |  |

**Table S4 | Variation partitioning of soil microbial community drivers.** Partial models are constrained for all variables contained in other models, giving the unique variation explained by the model under consideration, excluding the polder model where applicable. Dashes (-) indicate a model which was not tested due to limited replication at the landscape level.

| **A) Variation partitioning of PLFA data at the landscape scale** | | | | | | | |
| --- | --- | --- | --- | --- | --- | --- | --- |
| **Model** | | **R²adj (%)** | **F** | **p** | | **DF model** | **DF res** |
| *Soil characteristics* | full | 16.07 | - | - | - | 3 | 59 |
|  | Partial | 9.30 | - | - | - | 3 | 50 |
| *Vegetation community* | Full | 4.62 | - | - | - | 4 | 58 |
|  | Partial | 0.16 | - | - | - | 4 | 50 |
| *Polder* | Full | 19.83 | 8.67 | 0.0002 | *** | 3 | 59 |
|  | Partial | 2.93 | 1.01 | 0.3658 | ns | 3 | 59 |
| *Nutrient management* | Full | 4.48 | - | - | - | 5 | 57 |
|  | Partial | 0.33 | - | - | - | 5 | 50 |
|  |  |  |  |  |  |  |  |
| **B) Variation partitioning of CLPP data at the landscape scale** | | | | | | | |
| **Model** | | **R²adj (%)** | **F** | **p** | | **DF model** | **DF res** |
| *Soil characteristics* | Full | 2.85 | - | - | - | 8 | 135 |
|  | Partial | 1.27 | - | - | - | 8 | 130 |
| *Vegetation community* | Full | 2.17 | - | - | - | 5 | 138 |
|  | Partial | 0.59 | - | - | - | 5 | 130 |
| *Polder* | Full | 4.99 | 29.10 | 0.0020 | ** | 5 | 138 |
|  | Partial | 1.04 | 10.21 | 0.4434 | ns | 5 | 119 |
| *Nutrient management* | Full | 0.00 | - | - | - | NA | NA |
|  | Partial | 0.00 | - | - | - | NA | NA |
|  |  |  |  |  |  |  |  |
| **C) Variation partitioning of PLFA data at the local scale** | | | | | | | |
| **Model** | | **R²adj (%)** | **F** | **p** | | **DF model** | **DF res** |
| *Soil characteristics* | Full | 0.00 | NA | NA | ns | NA | NA |
|  | Partial | 0.00 | NA | NA | ns | NA | NA |
| *Vegetation community* | Full | 0.00 | NA | NA | ns | NA | NA |
|  | Partial | 0.00 | NA | NA | ns | NA | NA |
| *Spatial patterns* | Full | 0.00 | NA | NA | ns | NA | NA |
|  | Partial | 0.00 | NA | NA | ns | NA | NA |
| *Nutrient management* | Full | 4.02 | 2.59 | 0.0241 | * | 2 | 58 |
|  | Partial | 4.02 | 2.59 | 0.0241 | * | 2 | 58 |
| **D) Variation partitioning of CLPP data at the local scale** | | | | | | | |
| **Model** | | **R²adj (%)** | **F** | **p** | | **DF model** | **DF res** |
| *Soil characteristics* | Full | 0.00 | NA | NA | ns | NA | NA |
|  | Partial | 0.00 | NA | 1.0000 | ns | NA | NA |
| *Vegetation community* | Full | 8.75 | 4.50 | 0.0010 | *** | 4 | 134 |
|  | Partial | 5.14 | 3.09 | 0.0051 | ** | 4 | 128 |
| *Spatial patterns* | Full | 8.76 | 3.34 | 0.0010 | *** | 6 | 132 |
|  | Partial | 5.16 | 2.42 | 0.0007 | *** | 6 | 128 |
| *Nutrient management* | Full | 8.76 | NA | NA | ns | NA | NA |
|  | Partial | 0.00 | NA | 1.0000 | ns | NA | NA |

**Table S5 | Importance of different drivers in explaining the full variation in soil microbial community structure (PLFA) and functional capacity (CLPP) irrespective of spatial scale.**

|  | **Model** | **R²adj (%)** | **F** | **p** | | **DF res** |
| --- | --- | --- | --- | --- | --- | --- |
| *PLFA* | *Soil characteristics* | 19.76 | 2.27 | 0.0034 | ** | 50 |
|  | *Vegetation composition* | 15.11 | 2.58 | 0.0069 | ** | 55 |
|  | *Polder* | 19.83 | 8.67 | 0.0002 | *** | 60 |
|  | *Nutrient management* | 10.77 | 2.07 | 0.0276 | * | 55 |
| CLPP | *Soil characteristics* | 9.50 | 2.07 | 0.0131 | * | 129 |
|  | *Vegetation composition* | 9.15 | 2.80 | 0.0001 | *** | 135 |
|  | *Polder* | 4.99 | 2.50 | 0.0022 | ** | 138 |
|  | *Nutrient management* | 3.38 | 1.42 | 0.1309 | ns | 131 |
|  |  |  |  |  |  |  |
|  | |  |  |  |  |  |
|  |  | | | |  |  |
