# Supplementary material for: Local Functioning, Landscape Structuring: Drivers of Soil Microbial Community Structure and Function in Peatlands
Source: Front Microbiol. 2018 Sep 3;9:2060. doi: 10.3389/fmicb.2018.02060 (PMC6129579; doi:10.3389/fmicb.2018.02060)
Supplement: Supplementary file 3 [file Table_3.DOCX]

#libraries

library(ape)

library(packfor)

library(spacemakeR)

library(ade4)

library(spdep)

library(vegan)

library(PCNM)

library(foreach)

library(far)

library(foreach)

library(segmented)

library(compositions)

library(doSNOW)

library(gradientForest)

source("C:/Users/Sven/Documents/R/Scripts/beta-diversity/beta.div.R")

source("C:/Users/Sven/Documents/R/Scripts/beta-diversity/beta.div.comp.R")

source("C:/Users/Sven/Documents/R/Scripts/beta-diversity/LCBD.comp.R")

source("C:/Users/Sven/Documents/R/NEwR updated material/NEwR functions/cleanplot.pca.R")

source("C:/Users/Sven/Documents/R/Scripts/summarySE.R")

#Load data

dbSPEC_RAW = read.csv("C:/Users/Sven/Documents/PhD/Data/Veg 2014/20150803_VEG_CONVERSION.csv", header=TRUE, sep=",", dec = ".", row.names=1, stringsAsFactors=FALSE)

dbMIC_RAW = read.csv("C:/Users/Sven/Documents/PhD/Data/Microbial/20150806_PLFA_allremoved.csv", header=TRUE, sep=",", dec = ".", row.names=1, stringsAsFactors=FALSE)

dbENV_RAW = read.csv("C:/Users/Sven/Documents/PhD/Data/Env/20150406_ENV_VEG.csv", header=TRUE, sep=",", dec = ".", row.names=1)

dbLU_RAW = read.csv('C:/Users/Sven/Documents/PhD/Data/LandUse/201508_FERT_ALL_MICROBIAL.csv', header=TRUE, sep=",", dec = ".", row.names=1, stringsAsFactors=TRUE)

dbXY_RAW = read.table("C:/Users/Sven/Documents/PhD/Data/Location_20121314.txt", header=TRUE, sep=" ", row.names=1)

dbAREA_RAW = read.csv("C:/Users/Sven/Documents/PhD/Data/AreaDummies.csv", header=TRUE, sep=",", dec = ".", row.names=1)

dbSOIL_RAW = read.csv("C:/Users/Sven/Documents/PhD/Data/SoilType/20141202_SoilType_BOFEKPAWN.csv", header=TRUE, sep=",", dec = ".", row.names=1)

#____________________________________________________________________________

#_____________________________________________________

#_______________________________________

#---BETWEEN AREAS ANALYSIS-------------

#_______________________________________

#_____________________________________________________

#____________________________________________________________________________

lMIC_SEL = c('Area','Site','treatment','standard..19.00.', 'cy19.0')

dbMIC_RAW = dbMIC_RAW[,-which(colnames(dbMIC_RAW) %in% lMIC_SEL)]

dbMIC_PERC=data.frame()

for(nMIC_ROW in 1:nrow(dbMIC_RAW)){

dbMIC_PERC=rbind(dbMIC_PERC,(dbMIC_RAW[nMIC_ROW,]/colSums(dbMIC_RAW)))

}

dbMIC_RAW=dbMIC_PERC

lAREAS = c('H','I','O')

#Selection of subset of vegetation based on Ellenberg values

remove(dbSPEC)

nLOW = 4 #Lower bound ellenberg value

nUP = 10 #Upper bound ellenberg value

#build a dataframe of species data within a given ellenberg range

for(r in 1: ncol(dbSPEC_RAW)){

if(as.numeric(dbSPEC_RAW[c(2),c(r)]) <= max(nUP,nLOW) && as.numeric(dbSPEC_RAW[c(2),c(r)]) >= min(nLOW,nUP) ){

if(exists("dbSPEC")){

dbSPEC = cbind(dbSPEC, dbSPEC_RAW[c(r)])

}else{

dbSPEC = dbSPEC_RAW[c(r)]

}

}

}

dbSPEC = data.matrix(dbSPEC[-c(1,2),])

dbSPEC = subset(dbSPEC, grepl(25, row.names(dbSPEC)) != TRUE )#remove site 25, main waterway site (if it exists) #not relevant in this case

dbSPEC = subset(dbSPEC, substr(row.names(dbSPEC), 1, 1) %in% lAREAS & row.names(dbSPEC) %in% row.names(dbMIC_RAW))

if(length(which(colSums(dbSPEC) == 0))>0){dbSPEC = dbSPEC[,-(which(colSums(dbSPEC) == 0))]} #remove all zero columns

if(length(which(rowSums(dbSPEC) == 0))>0){dbSPEC = dbSPEC[-(which(rowSums(dbSPEC) == 0)),]} #remove all zero rows

lENVVEG_BANK = c("SOIL_PH","SOIL_C_BANK","SOIL_NTOT_BANK","SOIL_PTOT_BANK","ANGLE_MEAN",

"MORPH_WIDTH_BANK_MEAN","SOIL_MOISTURE")

#Select Soil Type variables

lSOIL_DESEL = c('Area','Site','Year_','POINT_X','POINT_Y','BOFEK2012','BOFEK_LABEL','PAWN')

dfSOIL1 = dbSOIL_RAW[,!(colnames(dbSOIL_RAW) %in% lSOIL_DESEL)]

dfSOIL2 = dfSOIL1[,which(grepl("PAWN", names(dfSOIL1))==1)]

dfSOIL3 = subset(dfSOIL2, substr(row.names(dfSOIL2), 1, 1) %in% lAREAS)

dfSOIL4 = subset(dfSOIL3, grepl(25, row.names(dfSOIL3)) != TRUE )#remove site 25 (if it exists)

dfSOIL_SEL = dfSOIL4[which(row.names(dfSOIL4) %in% row.names(dbMIC_RAW)),which(colnames(dfSOIL4) %in% names(which(colSums(dfSOIL4[,-1])!=0)))]

dfSOIL_SEL$SOIL_FAC = as.factor(dfSOIL4$PAWN_LABEL[which(row.names(dfSOIL4) %in% row.names(dbMIC_RAW))])

#Select abiotic variables

dfENV = dbENV_RAW[,(colnames(dbENV_RAW) %in% lENVVEG_BANK)]

dfENV1 = subset(dfENV, substr(row.names(dfENV), 1, 1) %in% lAREAS)

dfENV2 = subset(dfENV1, grepl(25, row.names(dfENV1)) != TRUE )#remove site 25 (if it exists)

dfENV3 = dfENV2[which(row.names(dfENV2) %in% row.names(dbMIC_RAW)),which(colSums(is.na(dfENV2))==0)]

dfENV_SEL = dfENV3

dfENV_WORK = cbind.data.frame(dfENV_SEL, dfSOIL_SEL[,ncol(dfSOIL_SEL),drop=F])

dfENV_WORK_FS = cbind.data.frame(dfENV_SEL, dfSOIL_SEL[,-ncol(dfSOIL_SEL),drop=F])

#Select areas

dfAREA1 = subset(dbAREA_RAW[,-1], substr(row.names(dbAREA_RAW[,-1]), 1, 1) %in% lAREAS)

dfAREA2 = subset(dfAREA1, grepl(25, row.names(dfAREA1)) != TRUE )#remove site 25 (if it exists)

dfAREA_SEL = dfAREA2[which(row.names(dfAREA2) %in% row.names(dbMIC_RAW)),which(colnames(dfAREA2) %in% names(which(colSums(dfAREA2[,-1])!=0)))]

dfAREA_SEL$AREA_FAC = as.factor(dfAREA2$Area[which(row.names(dfAREA2) %in% row.names(dbMIC_RAW))])

#Select land use

dfLU1 = subset(dbLU_RAW, substr(row.names(dbLU_RAW), 1, 1) %in% lAREAS)

dfLU_SEL = dfLU1[which(row.names(dfLU1) %in% row.names(dbMIC_RAW)), c(9:12),drop=F]

dfLU_WORK_FS = dfLU_SEL

# dfLU_WORK=dfLU_SEL

# fFORM_FAC = as.formula(paste(" ~ ","FACTOR", " -1", sep=""))

# dfLU_WORK_FS1 = as.data.frame(model.matrix(fFORM_FAC, data=dfLU_SEL))

# dfLU_WORK_FS =dfLU_WORK_FS1[,-which(colSums(dfLU_WORK_FS1)==0)]

# colnames(dfLU_WORK_FS)=substr(colnames(dfLU_WORK_FS),7, nchar(colnames(dfLU_WORK_FS)))

#-------------

#-----MEM-----

#_____________

#--------MICROBIAL COMM DISTANCE MATRIX-----------------

#---Create distance matrices for between area analyses---

#We make a full data bray-curtis distance matrix and then put all site combinations within areas to zero

distMIC = vegdist(dbMIC_RAW, "bray")

dfDIST_MIC = data.matrix(distMIC)

dfDIST_SAVE=data.frame(matrix(NA,nrow(dfDIST_MIC), 0))

for(nCOL_DIST in 1:ncol(dfDIST_MIC)){

dfDIST_COL = dfDIST_MIC[,nCOL_DIST,drop=F]

dfDIST_COL[which(substr(rownames(dfDIST_COL),1,1)==substr(colnames(dfDIST_COL),1,1)),]=0

dfDIST_SAVE=cbind.data.frame(dfDIST_SAVE,dfDIST_COL)

}

distMIC_BETWEEN = as.dist(dfDIST_SAVE)

dbMIC_PCOA = pcoa(distMIC_BETWEEN, correction="lingoes") #PCoA scores using Bray-Curtis dissimilarity, this can then be used in an RDA to create a distance based RDA

dfMIC_PCOA_LM = cbind(as.data.frame(dbMIC_PCOA$values[,'Rel_corr_eig']), as.data.frame(seq(1:length(dbMIC_PCOA$values[,'Rel_corr_eig']))))

colnames(dfMIC_PCOA_LM) = c('x', 'y')

lmMIC_PCOA = lm(x ~ y,data=dfMIC_PCOA_LM)

lmMIC_PCOA_SEG = segmented(lmMIC_PCOA, seg.Z=~y, psi=list(y=c(max(which(dbMIC_PCOA$values[,'Rel_corr_eig'] >0.01)))),control=seg.control(n.boot=10000))#probably a bit excessive, but it is to avoid deviation from the true break point (which can lead to drastically different results as seen on 16-01-2015 together with Amber)

nMIC_PCOA_POS = ceiling(lmMIC_PCOA_SEG$psi[2]) #round up the estimated break point to whole variables

dbMIC_PCOA=(dbMIC_PCOA$vectors.cor)[,1:nMIC_PCOA_POS]

colnames(dbMIC_PCOA) = paste("PCOA_VAR",seq(1,ncol(dbMIC_PCOA),le=ncol(dbMIC_PCOA)),sep="")#very important as otherwise there are no headers to select variables on for e.g. gradientForest function

rownames(dbMIC_PCOA) = rownames(dbMIC_RAW)

vMIC_EXPLVAR = dfMIC_PCOA_LM[1:nMIC_PCOA_POS,1]

#vegetation PCOA

distVEG = vegdist(dbSPEC, "bray")

dbVEG_PCOA = pcoa(distVEG, correction="lingoes") #PCoA scores using Bray-Curtis dissimilarity, this can then be used in an RDA to create a distance based RDA

dfVEG_PCOA_LM = cbind(as.data.frame(dbVEG_PCOA$values[,'Rel_corr_eig']), as.data.frame(seq(1:length(dbVEG_PCOA$values[,'Rel_corr_eig']))))

colnames(dfVEG_PCOA_LM) = c('x', 'y')

lmVEG_PCOA = lm(x ~ y,data=dfVEG_PCOA_LM)

lmVEG_PCOA_SEG = segmented(lmVEG_PCOA, seg.Z=~y, psi=list(y=c(max(which(dbVEG_PCOA$values[,'Rel_corr_eig'] >0.01)))),control=seg.control(n.boot=10000))#probably a bit excessive, but it is to avoid deviation from the true break point (which can lead to drastically different results as seen on 16-01-2015 together with Amber)

nVEG_PCOA_POS = ceiling(lmVEG_PCOA_SEG$psi[2]) #round up the estimated break point to whole variables

dbVEG_PCOA=(dbVEG_PCOA$vectors.cor)[,1:nVEG_PCOA_POS]

colnames(dbVEG_PCOA) = paste("PCOA_VAR",seq(1,ncol(dbVEG_PCOA),le=ncol(dbVEG_PCOA)),sep="")#very important as otherwise there are no headers to select variables on for e.g. gradientForest function

rownames(dbVEG_PCOA) = rownames(dbSPEC)

vVEG_EXPLVAR = dfVEG_PCOA_LM[1:nVEG_PCOA_POS,1]

#--between areas MEM--

dbXY = subset(dbXY_RAW[c(4,5)], grepl(25, row.names(dbXY_RAW)) != TRUE )#remove site 25 (if it exists)

dbXY = subset(dbXY[which(row.names(dbXY) %in% row.names(dbMIC_RAW)),], substr(row.names(dbXY[which(row.names(dbXY) %in% row.names(dbMIC_RAW)),]), 1, 1) %in% lAREAS) #Subselection of specific area based on area letter in lAREA

#select subset of XY by area and define distance related variables

mXY = as.matrix(dbXY) #XY coordinates

mDISTXY = dist(dbXY) #Distance matrix

##fDIST = function(D, dmax, y) {1 - (D/dmax)} #Weighing function for spatial weighing (B)

##(lTHRESH <- seq(give.thresh(mDISTXY), to=max(mDISTXY), le=1000))

###(lTHRESH <- seq(149584, to=149584, le=1))

##nbCON <- lapply(lTHRESH, dnearneigh, x=mXY, d1=0)

##

##memTHRESH <- lapply(nbCON, function(x) test.W(x, Y=dbMIC_PCOA, f=fDIST, dmax=max(mDISTXY), xy=mXY, MEM.autocor = c("positive")))

##memAIC_MIN <- sapply(memTHRESH, function(x) min(x$best$AICc, na.rm=TRUE))

##

##memMEMMODEL <- unlist(memTHRESH[which.min(memAIC_MIN)], recursive=FALSE)

### MEM variables selected in the best model

##lMEMID <- memMEMMODEL$best$ord

##vMEM_TOP <- memMEMMODEL$best$vectors[,c(lMEMID)]

### Adjusted R2 of best model

##RsquareAdj(memMEMMODEL$best$R2[which.min(memMEMMODEL$best$AICc)], nrow(dbMIC_PCOA), length(lMEMID))

##dfMEMMIC = as.data.frame(vMEM_TOP)

##colnames(dfMEMMIC) = paste("MEM_VAR_",seq(1,ncol(dfMEMMIC),le=ncol(dfMEMMIC)),sep="")

#non-cut down MEMS

mDISTXY2=1 - (mDISTXY/max(mDISTXY))

lwDIST = mat2listw(as.matrix(mDISTXY2))

mMEMSPEC = scores.listw(lwDIST, echo=TRUE)

vMEMSPEC = mMEMSPEC$vectors

vMORANI = test.scores(mMEMSPEC, lwDIST, 9999)

# Save MEMs with positive spatial correlation that are significant

vMORANI_POS = which(vMORANI[,1] > -1/(nrow(vMEMSPEC)-1))

dfMEMMIC = as.data.frame(vMEMSPEC[,vMORANI_POS])

colnames(dfMEMMIC) = paste("MEM_VAR_",seq(1,ncol(dfMEMMIC),le=ncol(dfMEMMIC)),sep="")

dfSPACE_WORK = cbind.data.frame(dfMEMMIC)#,dfAREA_SEL[,1:3])

dfSPACE_WORK_FS = cbind.data.frame(dfMEMMIC)#,dfAREA_SEL[,1:3])

#-----------------------------------

#---GRADIENT FOREST ANALYSIS--------

#___________________________________

#Select variables using GF (VI <0.001)

#Use these variables in an RDA and varpart (no FS)

#then use a gradient forest to identify species most affected by Env, Man and space

##---GRADIENT FOREST

dfPREDICT =cbind(dfENV_WORK_FS, dfSPACE_WORK_FS, dfLU_WORK_FS, dbVEG_PCOA)#dfVEG_WORK) #untransformed vegetation data goes in here

gf_COMM= gradientForest(cbind(dfPREDICT, dbMIC_PCOA), predictor.vars = colnames(dfPREDICT), response.vars = colnames(dbMIC_PCOA), ntree = 9999, mtry=ceiling(sqrt(ncol(dfPREDICT))), maxLevel=floor(log2(nrow(dfPREDICT) * 0.368/2)), corr.threshold=0.5)

vIMP = importance(gf_COMM, type="W")

fR2_GF_COMM = sum(importance(gf_COMM,type="S"))

gf_COMM$imp.rsq

#Idea: first run GF then RDA

#Select variables from GF with VI higher than 0.001 and use these in varpart without forsel

lVARS_GF_SEL = names(vIMP[which(vIMP>0.001)])

dfENV_SEL_GF = dfENV_WORK_FS[,which(colnames(dfENV_WORK_FS) %in% lVARS_GF_SEL),drop=F]

dfLU_SEL_GF = dfLU_WORK_FS[,which(colnames(dfLU_WORK_FS) %in% lVARS_GF_SEL),drop=F]

dfSPACE_SEL_GF = dfSPACE_WORK_FS[,which(colnames(dfSPACE_WORK_FS) %in% lVARS_GF_SEL), drop=F]

dfVEG_SEL_GF = dbVEG_PCOA[,which(colnames(dbVEG_PCOA) %in% lVARS_GF_SEL), drop=F]

#-----------------------------------

#---RDA ANALYSIS--------------------

#___________________________________

##RDA for between region differences

dfVEG_WORK =dfVEG_SEL_GF

dfVEG_SEL_GF = dfVEG_SEL_GF

dfENV_SEL_GF = dfENV_SEL_GF#cbind.data.frame(dfENV_SEL, dfSOIL_SEL[,ncol(dfSOIL_SEL),drop=F])

dfENV_SEL_GF = dfENV_SEL_GF#cbind.data.frame(dfENV_SEL, dfSOIL_SEL[,-ncol(dfSOIL_SEL),drop=F])

dfSPACE_SEL_GF = dfSPACE_SEL_GF#cbind.data.frame(dfMEMMIC, dfAREA_SEL[,4,drop=F])

dfSPACE_SEL_GF = dfSPACE_SEL_GF#cbind.data.frame(dfMEMMIC, dfAREA_SEL[,-4,drop=F])

dfLU_SEL_GF=dfLU_SEL_GF#dfLU_SEL

#Create formula's for RDA

sENV = ""

for(sVAR in colnames(dfENV_SEL_GF)){

if(sENV == ""){

sENV = sVAR

}else if(sENV != ""){

sENV = paste(sENV, sVAR, sep="+")

}

}

sSPACE = ""

for(sVAR in colnames(dfSPACE_SEL_GF)){

if(sSPACE == ""){

sSPACE = sVAR

}else if(sSPACE != ""){

sSPACE = paste(sSPACE, sVAR, sep="+")

}

}

sLU = ""

for(sVAR in colnames(dfLU_SEL_GF)){

if(sLU == ""){

sLU = sVAR

}else if(sLU != ""){

sLU = paste(sLU, sVAR, sep="+")

}

}

sVEG = ""

for(sVAR in colnames(dfVEG_SEL_GF)){

if(sVEG == ""){

sVEG = sVAR

}else if(sVEG != ""){

sVEG = paste(sVEG, sVAR, sep="+")

}

}

#sAREA_FORM = paste(" + ", "Condition(",colnames(dfAREA_SEL[,4,drop=F]),")", sep="")

fRDA_ENV = as.formula(paste('dbMIC_PCOA',' ~ ',sENV,sep=""))

fRDA_SPACE = as.formula(paste('dbMIC_PCOA',' ~ ',sSPACE,sep=""))

fRDA_LU = as.formula(paste('dbMIC_PCOA',' ~ ',sLU,sep=""))

fRDA_VEG = as.formula(paste('dbMIC_PCOA',' ~ ',sVEG,sep=""))

nPERM=99999

#--ENVIRONMENTAL+SOIL--

rdaSPEC_ENV = rda(fRDA_ENV, data=dfENV_SEL_GF)

fR2_SPEC_ENV=RsquareAdj(rdaSPEC_ENV)$adj.r.squared #extract the adjusted R2

testSPEC_ENV=anova(rdaSPEC_ENV,permutations=nPERM)

fFSTAT_SPEC_ENV=testSPEC_ENV$F[1] #extract the F statistic

fPSTAT_SPEC_ENV=testSPEC_ENV$Pr[1] #extract the p value

fsRDA_ENV = try(forward.sel(dbMIC_PCOA, dfENV_SEL_GF, nperm=nPERM, adjR2thresh=fR2_SPEC_ENV))

if(class(fsRDA_ENV)=="try-error" | fPSTAT_SPEC_ENV >0.05){lENV_FS = c()}else{lENV_FS = c(unlist(as.data.frame(fsRDA_ENV[,2])))}

#--SPATIAL--

rdaSPEC_MEM = rda(fRDA_SPACE, data=dfSPACE_SEL_GF)

fR2_SPEC_MEM=RsquareAdj(rdaSPEC_MEM)$adj.r.squared #extract the adjusted R2

testSPEC_MEM=anova(rdaSPEC_MEM,permutations=nPERM)

fFSTAT_SPEC_MEM=testSPEC_MEM$F[1] #extract the F statistic

fPSTAT_SPEC_MEM=testSPEC_MEM$Pr[1] #extract the p value

fsRDA_MEM = try(forward.sel(dbMIC_PCOA, dfSPACE_SEL_GF, nperm=nPERM, adjR2thresh=fR2_SPEC_MEM))

if(class(fsRDA_MEM)=="try-error" | fPSTAT_SPEC_MEM >0.05){lMEM_FS = c()}else{lMEM_FS = c(unlist(as.data.frame(fsRDA_MEM[,2])))}

#--MANAGEMENT--

rdaSPEC_MAN = rda(fRDA_LU, data=dfLU_SEL_GF)

fR2_SPEC_MAN=RsquareAdj(rdaSPEC_MAN)$adj.r.squared #extract the adjusted R2

testSPEC_MAN=anova(rdaSPEC_MAN,permutations =nPERM)

fFSTAT_SPEC_MAN=testSPEC_MAN$F[1] #extract the F statistic

fPSTAT_SPEC_MAN=testSPEC_MAN$Pr[1] #extract the p value

fsRDA_MAN = try(forward.sel(dbMIC_PCOA, dfLU_SEL_GF, nperm=nPERM, adjR2thresh=fR2_SPEC_MAN))

if(class(fsRDA_MAN)=="try-error" | fPSTAT_SPEC_MAN >0.05){lMAN_FS = c()}else{lMAN_FS = c(unlist(as.data.frame(fsRDA_MAN[,2])))}

#--VEGETATION--

rdaSPEC_VEG = rda(fRDA_VEG, data=as.data.frame(dfVEG_SEL_GF))

fR2_SPEC_VEG=RsquareAdj(rdaSPEC_VEG)$adj.r.squared #extract the adjusted R2

testSPEC_VEG=anova(rdaSPEC_VEG,permutations =nPERM)

fFSTAT_SPEC_VEG=testSPEC_VEG$F[1] #extract the F statistic

fPSTAT_SPEC_VEG=testSPEC_VEG$Pr[1] #extract the p value

fsRDA_VEG = try(forward.sel(dbMIC_PCOA, dfVEG_SEL_GF, nperm=nPERM,adjR2thresh=fR2_SPEC_VEG))

if(class(fsRDA_VEG)=="try-error" | fPSTAT_SPEC_VEG >0.05){lVEG_FS = c()}else{lVEG_FS = c(unlist(as.data.frame(fsRDA_VEG[,2])))}

#--COMBINED MODEL---

dfALL_WORK_FS = cbind.data.frame(dfENV_SEL_GF[,lENV_FS,drop=F], dfSPACE_SEL_GF[,lMEM_FS, drop=F], dfLU_SEL_GF[,lMAN_FS,drop=F], dfVEG_SEL_GF[,lVEG_FS,drop=F])

#dfALL_WORK_FS = cbind.data.frame(dfENV_SEL_GF, dfSPACE_SEL_GF, dfLU_SEL_GF_FS, dfVEG_SEL_GF)

#Create a total model

rdaSPEC_COMB = rda(dbMIC_PCOA~., dfALL_WORK_FS)

fR2_SPEC_COMB=RsquareAdj(rdaSPEC_COMB)$adj.r.squared #extract the adjusted R2

testSPEC_COMB=anova(rdaSPEC_COMB,permutations=nPERM)

#anova(rdaSPEC_COMB,permutations=nPERM, by='term')

fFSTAT_SPEC_COMB=testSPEC_COMB$F[1] #extract the F statistic

fPSTAT_SPEC_COMB=testSPEC_COMB$Pr[1] #extract the p value

#forward.sel(dbMIC_PCOA, dfALL_WORK_FS, nperm=nPERM,adjR2thresh=fR2_SPEC_COMB)

#vartpart plot

pdf(paste("C:/Users/Sven/Documents/PhD/R/Data/RESULTS_MIC/","VARPART_BETWEEN_POLDERS",".pdf",sep=""),width=10, height=7, pointsize=14)

if(length(lENV_FS)>0 & length(lMEM_FS)>0 & length(lMAN_FS)>0 & length(lVEG_FS)>0){

lPART = c('ENV', 'SPACE', 'FERTILIZATION', 'VEG')

plot(varpart(dbMIC_PCOA, dfENV_SEL_GF[,lENV_FS,drop=F], dfSPACE_SEL_GF[,lMEM_FS, drop=F], dfLU_SEL_GF[,lMAN_FS,drop=F], dfVEG_WORK[,lVEG_FS,drop=F]), Xnames=lPART)

}else if(length(lENV_FS)>0 & length(lMEM_FS)>0 & length(lMAN_FS)>0 ){

lPART = c('ENV', 'SPACE', 'FERTILIZATION')

plot(varpart(dbMIC_PCOA, dfENV_SEL_GF[,lENV_FS,drop=F], dfSPACE_SEL_GF[,lMEM_FS, drop=F], dfLU_SEL_GF[,lMAN_FS,drop=F]), Xnames=lPART)

}else if(length(lENV_FS)>0 & length(lMEM_FS)>0 & length(lVEG_FS)>0){

lPART = c('ENV', 'SPACE', 'VEG')

plot(varpart(dbMIC_PCOA, dfENV_SEL_GF[,lENV_FS,drop=F], dfSPACE_SEL_GF[,lMEM_FS, drop=F], dfVEG_WORK[,lVEG_FS,drop=F]), Xnames=lPART)

}else if(length(lENV_FS)>0 & length(lMAN_FS)>0 & length(lVEG_FS)>0){

lPART = c('ENV', 'FERTILIZATION', 'VEG')

plot(varpart(dbMIC_PCOA, dfENV_SEL_GF[,lENV_FS,drop=F], dfLU_SEL_GF[,lMAN_FS,drop=F], dfVEG_WORK[,lVEG_FS,drop=F]), Xnames=lPART)

}else if(length(lENV_FS)>0 & length(lMEM_FS)>0 & length(lMAN_FS)>0 & length(lVEG_FS)>0){

lPART = c('SPACE', 'FERTILIZATION', 'VEG')

plot(varpart(dbMIC_PCOA, dfSPACE_SEL_GF[,lMEM_FS, drop=F], dfLU_SEL_GF[,lMAN_FS,drop=F], dfVEG_WORK[,lVEG_FS,drop=F]), Xnames=lPART)

}else if(length(lENV_FS)>0 & length(lMEM_FS)>0 & length(lMAN_FS)>0 & length(lVEG_FS)>0){

lPART = c('ENV', 'SPACE')

plot(varpart(dbMIC_PCOA, dfENV_SEL_GF[,lENV_FS,drop=F], dfSPACE_SEL_GF[,lMEM_FS, drop=F]), Xnames=lPART)

}else if(length(lENV_FS)>0 & length(lVEG_FS)>0){

lPART = c('ENV', 'VEG')

plot(varpart(dbMIC_PCOA, dfENV_SEL_GF[,lENV_FS,drop=F], dfVEG_WORK[,lVEG_FS,drop=F]), Xnames=lPART)

}else if(length(lENV_FS)>0 & length(lMAN_FS)>0){

lPART = c('ENV', 'FERTILIZATION')

plot(varpart(dbMIC_PCOA, dfENV_SEL_GF[,lENV_FS,drop=F], dfLU_SEL_GF[,lMAN_FS,drop=F]), Xnames=lPART)

}else if(length(lMEM_FS)>0 & length(lVEG_FS)>0){

lPART = c('SPACE', 'VEG')

plot(varpart(dbMIC_PCOA, dfSPACE_SEL_GF[,lMEM_FS, drop=F], dfVEG_WORK[,lVEG_FS,drop=F]), Xnames=lPART)

}else if(length(lMEM_FS)>0 & length(lMAN_FS)>0){

lPART = c('SPACE', 'FERTILIZATION')

plot(varpart(dbMIC_PCOA, dfSPACE_SEL_GF[,lMEM_FS, drop=F], dfLU_SEL_GF[,lMAN_FS,drop=F]), Xnames=lPART)

}else if(length(lMAN_FS)>0 & length(lVEG_FS)>0){

lPART = c('FERTILIZATION', 'VEG')

plot(varpart(dbMIC_PCOA, dfLU_SEL_GF[,lMAN_FS,drop=F], dfVEG_WORK[,lVEG_FS,drop=F]), Xnames=lPART)

}else{

print('no variation partitioning possible')

}

dev.off()

##

##lPART = c('ENV', 'SPACE', 'FERTILIZATION', 'VEG')

##windows()

##plot(varpart(dbMIC_PCOA, dfENV_WORK_FS[,lENV_FS,drop=F], dfSPACE_WORK_FS[,lMEM_FS, drop=F], dfLU_WORK_FS[,lMAN_FS,drop=F], dfVEG_WORK[,lVEG_FS,drop=F]), Xnames=lPART)

##plot(varpart(dbMIC_PCOA, dfENV_WORK_FS, dfSPACE_WORK_FS, dfLU_WORK_FS, dfVEG_WORK), Xnames=lPART)

##

##windows()

##lPART = c('ENV', 'SPACE', 'VEG')

###plot(varpart(dbMIC_PCOA, dfENV_WORK_FS[,lENV_FS,drop=F], dfSPACE_WORK_FS[,lMEM_FS, drop=F], dfVEG_WORK[,lVEG_FS,drop=F]), Xnames=lPART)

##plot(varpart(dbMIC_PCOA, dfENV_WORK_FS, dfSPACE_WORK_FS, dfVEG_WORK), Xnames=lPART)

##

#--forward selected (full) and partial models (marginal partitions)

dfOUTPUT_RDA = data.frame(matrix(NA,0,5))

lMODELS_RDA = c('FULL_ENV', 'PURE_ENV','FULL_SPACE','PURE_SPACE','FULL_FERTILIZATION','PURE_FERTILIZATION','FULL_VEGETATION','PURE_VEGETATION')

for(sMODEL_RDA in lMODELS_RDA){

if(sMODEL_RDA == 'PURE_ENV'){dfEXPL = dfENV_SEL_GF[,lENV_FS,drop=F]

dfCONDITION = dfALL_WORK_FS[,-which(colnames(dfALL_WORK_FS) %in% colnames(dfEXPL))]}

if(sMODEL_RDA == 'PURE_SPACE'){dfEXPL = dfSPACE_SEL_GF[,lMEM_FS, drop=F]

dfCONDITION = dfALL_WORK_FS[,-which(colnames(dfALL_WORK_FS) %in% colnames(dfEXPL))]}

if(sMODEL_RDA == 'PURE_FERTILIZATION'){dfEXPL = dfLU_SEL_GF[,lMAN_FS,drop=F]

dfCONDITION = dfALL_WORK_FS[,-which(colnames(dfALL_WORK_FS) %in% colnames(dfEXPL))]}

if(sMODEL_RDA == 'PURE_VEGETATION'){dfEXPL = dbVEG_PCOA[,lVEG_FS,drop=F]

dfCONDITION = dfALL_WORK_FS[,-which(colnames(dfALL_WORK_FS) %in% colnames(dfEXPL))]}

if(sMODEL_RDA == 'FULL_ENV'){dfEXPL = dfENV_SEL_GF[,lENV_FS,drop=F]

dfCONDITION = data.frame(matrix(0,nrow(dfALL_WORK_FS),0))}

if(sMODEL_RDA == 'FULL_SPACE'){dfEXPL = dfSPACE_SEL_GF[,lMEM_FS, drop=F]

dfCONDITION = data.frame(matrix(0,nrow(dfALL_WORK_FS),0))}

if(sMODEL_RDA == 'FULL_FERTILIZATION'){dfEXPL = dfLU_SEL_GF[,lMAN_FS,drop=F]

dfCONDITION = data.frame(matrix(0,nrow(dfALL_WORK_FS),0))}

if(sMODEL_RDA == 'FULL_VEGETATION'){dfEXPL = dbVEG_PCOA[,lVEG_FS,drop=F]

dfCONDITION = data.frame(matrix(0,nrow(dfALL_WORK_FS),0))}

if(ncol(dfCONDITION) >0 & ncol(dfEXPL)>0){

rdaSPEC_PART = rda(dbMIC_PCOA, dfEXPL, dfCONDITION)

fR2_SPEC_PART=RsquareAdj(rdaSPEC_PART)$adj.r.squared #extract the adjusted R2

testSPEC_PART=anova(rdaSPEC_PART,permutations=nPERM)

fFSTAT_SPEC_PART=testSPEC_PART$F[1] #extract the F statistic

fPSTAT_SPEC_PART=testSPEC_PART$Pr[1] #extract the p value

sFS_VARS = paste(colnames(dfEXPL),collapse = "; ")

}else if(substr(sMODEL_RDA,1,4)=='FULL' & ncol(dfEXPL)>0){

rdaSPEC_PART = rda(dbMIC_PCOA, dfEXPL, dfCONDITION)

fR2_SPEC_PART=RsquareAdj(rdaSPEC_PART)$adj.r.squared #extract the adjusted R2

testSPEC_PART=anova(rdaSPEC_PART,permutations=nPERM)

fFSTAT_SPEC_PART=testSPEC_PART$F[1] #extract the F statistic

fPSTAT_SPEC_PART=testSPEC_PART$Pr[1] #extract the p value

sFS_VARS = paste(colnames(dfEXPL),collapse = "; ")

}else{

fR2_SPEC_PART=0

fFSTAT_SPEC_PART=0

fPSTAT_SPEC_PART=0

sFS_VARS = ""

}

dfOUT1 = t(as.data.frame(c(sMODEL_RDA, fR2_SPEC_PART,fFSTAT_SPEC_PART,fPSTAT_SPEC_PART,sFS_VARS)))

dfOUTPUT_RDA=rbind(dfOUTPUT_RDA,dfOUT1)

}

colnames(dfOUTPUT_RDA) = c('MODEL','adR2', 'F_val', 'p_val', 'FS_Variables')

write.table(dfOUTPUT_RDA,paste("C:/Users/Sven/Documents/PhD/R/Data/RESULTS_MIC/","VARPART_BETWEEN_POLDERS",".csv",sep=""), sep=',')

#run a gradient forest to determine variable importance

#Define dbSPEC data with discriminating abilities (not all zero, or 1 etc.)

dfSPEC_DISC=data.frame(matrix(NA,nrow(dbSPEC),0))

for(nSPEC_DISC in 1:ncol(dbSPEC)){

if(length(unique(dbSPEC[,nSPEC_DISC])) >2){

dfSPEC_DISC=cbind.data.frame(dfSPEC_DISC, dbSPEC[,nSPEC_DISC,drop=F])

}

}

dfPREDICT1 = cbind.data.frame(dfENV_WORK_FS, dfSPACE_WORK_FS, dfLU_WORK_FS, dfVEG_SEL_GF, dfSPEC_DISC)

#Do so for every model type seperately (ENV, MAN, SPACE, VEG, VEG_SPECS)

lMODEL = c('ENV', 'SPACE', 'FERTILIZATION', 'VEG', 'VEG_SPECS')

dfIMP_STORE=data.frame(matrix(NA, ncol(dfPREDICT1), ncol(dbMIC_PCOA)))

colnames(dfIMP_STORE) = colnames(dbMIC_PCOA)

rownames(dfIMP_STORE) = colnames(dfPREDICT1)

#make data frames expressing explained variation of different PCOA axes

dfMIC_EXPLVAR=t(as.data.frame(rep(as.data.frame(vMIC_EXPLVAR/sum(vMIC_EXPLVAR)), nrow(dfIMP_STORE))))

colnames(dfMIC_EXPLVAR) = colnames(dbMIC_PCOA)

rownames(dfMIC_EXPLVAR) = colnames(dfPREDICT1)

dfVEG_EXPLVAR=as.data.frame(rep(as.data.frame(vVEG_EXPLVAR[which(colnames(dbVEG_PCOA) %in% lVARS_GF_SEL)]/sum(vVEG_EXPLVAR[which(colnames(dbVEG_PCOA) %in% lVARS_GF_SEL)])), ncol(dfIMP_STORE)))

colnames(dfVEG_EXPLVAR) = colnames(dbMIC_PCOA)

rownames(dfVEG_EXPLVAR) = colnames(dfVEG_SEL_GF)

for(sMODEL in lMODEL){

if(sMODEL == 'ENV'){dfPREDICT = dfENV_WORK_FS}

if(sMODEL == 'SPACE'){dfPREDICT = dfSPACE_WORK_FS}

if(sMODEL == 'FERTILIZATION'){dfPREDICT = dfLU_WORK_FS}

if(sMODEL == 'VEG'){dfPREDICT = as.data.frame(dfVEG_SEL_GF)}

if(sMODEL == 'VEG_SPECS'){dfPREDICT = dfSPEC_DISC}

gf_SPEC = gradientForest(cbind(dfPREDICT, dbMIC_PCOA), predictor.vars = colnames(dfPREDICT), response.vars = colnames(dbMIC_PCOA), ntree = 9999, mtry=ceiling(sqrt(ncol(dfPREDICT))), maxLevel=floor(log2(nrow(dfPREDICT) * 0.368/2)), corr.threshold=0.5)

(vIMP = importance(gf_SPEC, type="W"))

dfIMP_STORE_MODEL = as.data.frame(gf_SPEC$imp.rsq)

dfIMP_STORE[which(rownames(dfIMP_STORE) %in% rownames(dfIMP_STORE_MODEL)),which(colnames(dfIMP_STORE) %in% colnames(dfIMP_STORE_MODEL))] = dfIMP_STORE_MODEL

}

dfIMP_STORE[is.na(dfIMP_STORE)]=0

dfIMP_STORE1=dfIMP_STORE * dfMIC_EXPLVAR

dfIMP_STORE1[which(rownames(dfIMP_STORE1) %in% rownames(dfVEG_EXPLVAR)),which(colnames(dfIMP_STORE1) %in% colnames(dfVEG_EXPLVAR))] = dfVEG_EXPLVAR * dfIMP_STORE1[which(rownames(dfIMP_STORE1) %in% rownames(dfVEG_EXPLVAR)),which(colnames(dfIMP_STORE1) %in% colnames(dfVEG_EXPLVAR))]

write.table(dfIMP_STORE1,paste("C:/Users/Sven/Documents/PhD/R/Data/RESULTS_MIC/","GF_VIMP_BETWEEN_POLDERS",".csv",sep=""), sep=',')

#consider hanging a significance value on this using permutation testing

#----END BETWEEN AREAS----

#____________________________________________________________________________

#Load data

dbSPEC_RAW = read.csv("C:/Users/Sven/Documents/PhD/Data/Veg 2014/20150803_VEG_CONVERSION.csv", header=TRUE, sep=",", dec = ".", row.names=1, stringsAsFactors=FALSE)

dbMIC_RAW = read.csv("C:/Users/Sven/Documents/PhD/Data/Microbial/20150806_PLFA_allremoved.csv", header=TRUE, sep=",", dec = ".", row.names=1, stringsAsFactors=FALSE)

dbENV_RAW = read.csv("C:/Users/Sven/Documents/PhD/Data/Env/20150406_ENV_VEG.csv", header=TRUE, sep=",", dec = ".", row.names=1)

dbLU_RAW = read.csv('C:/Users/Sven/Documents/PhD/Data/LandUse/201508_FERT_ALL_MICROBIAL.csv', header=TRUE, sep=",", dec = ".", row.names=1, stringsAsFactors=TRUE)

dbXY_RAW = read.table("C:/Users/Sven/Documents/PhD/Data/Location_20121314.txt", header=TRUE, sep=" ", row.names=1)

dbAREA_RAW = read.csv("C:/Users/Sven/Documents/PhD/Data/AreaDummies.csv", header=TRUE, sep=",", dec = ".", row.names=1)

dbSOIL_RAW = read.csv("C:/Users/Sven/Documents/PhD/Data/SoilType/20141202_SoilType_BOFEKPAWN.csv", header=TRUE, sep=",", dec = ".", row.names=1)

#____________________________________________________________________________

#_____________________________________________________

#_______________________________________

#---WITHIN AREAS ANALYSIS-------------

#_______________________________________

#_____________________________________________________

#____________________________________________________________________________

lMIC_SEL = c('Area','Site','treatment','standard..19.00.', 'cy19.0')

dbMIC_RAW = dbMIC_RAW[,-which(colnames(dbMIC_RAW) %in% lMIC_SEL)]

lAREAS = c('H','I','O')

#Selection of subset of vegetation based on Ellenberg values

remove(dbSPEC)

nLOW = 4 #Lower bound ellenberg value

nUP = 10 #Upper bound ellenberg value

#build a dataframe of species data within a given ellenberg range

for(r in 1: ncol(dbSPEC_RAW)){

if(as.numeric(dbSPEC_RAW[c(2),c(r)]) <= max(nUP,nLOW) && as.numeric(dbSPEC_RAW[c(2),c(r)]) >= min(nLOW,nUP) ){

if(exists("dbSPEC")){

dbSPEC = cbind(dbSPEC, dbSPEC_RAW[c(r)])

}else{

dbSPEC = dbSPEC_RAW[c(r)]

}

}

}

dbSPEC = data.matrix(dbSPEC[-c(1,2),])

dbSPEC = subset(dbSPEC, grepl(25, row.names(dbSPEC)) != TRUE )#remove site 25, main waterway site (if it exists) #not relevant in this case

dbSPEC = subset(dbSPEC, substr(row.names(dbSPEC), 1, 1) %in% lAREAS)

dbSPEC = dbSPEC[which(row.names(dbSPEC) %in% row.names(dbMIC_RAW)),]

if(length(which(colSums(dbSPEC) == 0))>0){dbSPEC = dbSPEC[,-(which(colSums(dbSPEC) == 0))]} #remove all zero columns

if(length(which(rowSums(dbSPEC) == 0))>0){dbSPEC = dbSPEC[-(which(rowSums(dbSPEC) == 0)),]} #remove all zero rows

lENVVEG_BANK = c("SOIL_PH","SOIL_C_BANK","SOIL_NTOT_BANK","SOIL_PTOT_BANK","ANGLE_MEAN",

"MORPH_WIDTH_BANK_MEAN","SOIL_MOISTURE")

#Select Soil Type variables

lSOIL_DESEL = c('Area','Site','Year_','POINT_X','POINT_Y','BOFEK2012','BOFEK_LABEL','PAWN')

dfSOIL1 = dbSOIL_RAW[,!(colnames(dbSOIL_RAW) %in% lSOIL_DESEL)]

dfSOIL2 = dfSOIL1[,which(grepl("PAWN", names(dfSOIL1))==1)]

dfSOIL3 = subset(dfSOIL2, row.names(dfSOIL2) %in% row.names(dbMIC_RAW))

dfSOIL4 = subset(dfSOIL3, grepl(25, row.names(dfSOIL3)) != TRUE )#remove site 25 (if it exists)

dfSOIL_SEL = dfSOIL4[,which(colnames(dfSOIL4) %in% names(which(colSums(dfSOIL4[,-1])!=0)))]

dfSOIL_SEL$SOIL_FAC = as.factor(dfSOIL4$PAWN_LABEL)

#Select abiotic variables

dfENV = dbENV_RAW[,(colnames(dbENV_RAW) %in% lENVVEG_BANK)]

dfENV1 = subset(dfENV, row.names(dfENV) %in% row.names(dbMIC_RAW))

dfENV2 = subset(dfENV1, grepl(25, row.names(dfENV1)) != TRUE )#remove site 25 (if it exists)

dfENV3 = dfENV2[,which(colSums(is.na(dfENV2))==0)]

dfENV_SEL = dfENV3

dfENV_WORK = cbind.data.frame(dfENV_SEL, dfSOIL_SEL[,ncol(dfSOIL_SEL),drop=F])

dfENV_WORK_FS = cbind.data.frame(dfENV_SEL, dfSOIL_SEL[,-ncol(dfSOIL_SEL),drop=F])

#Select areas

dfAREA1 = subset(dbAREA_RAW[,-1], row.names(dbAREA_RAW) %in% row.names(dbMIC_RAW))

dfAREA2 = subset(dfAREA1, grepl(25, row.names(dfAREA1)) != TRUE )#remove site 25 (if it exists)

dfAREA_SEL = dfAREA2[,which(colnames(dfAREA2) %in% names(which(colSums(dfAREA2[,-1])!=0)))]

dfAREA_SEL$AREA_FAC = as.factor(dfAREA2$Area)

#Select land use

dfLU1 = subset(dbLU_RAW, row.names(dbLU_RAW) %in% row.names(dbMIC_RAW))

dfLU_SEL = dfLU1[, c(9:12),drop=F]

dfLU_WORK=dfLU_SEL

#-------------

#-----MEM-----

#_____________

#--------MICROBIAL COMM DISTANCE MATRIX-----------------

#---Create distance matrices for within area analyses---

vMIC_EXPLVAR=c()

vVEG_EXPLVAR=c()

lHEAD_MICPCOA=c()

for(sAREA in lAREAS){lHEAD_MICPCOA = c(lHEAD_MICPCOA, paste("PCOA_VAR",seq(1,23,le=23),"_",sAREA,sep=""))}

dfPCOA_SAVE=data.frame(matrix(NA,nrow(dbMIC_RAW), length(lHEAD_MICPCOA)))

colnames(dfPCOA_SAVE)=lHEAD_MICPCOA

rownames(dfPCOA_SAVE) = rownames(dbMIC_RAW)

for(sAREA in lAREAS){

#--scaled per plfa, raw data

dbMIC_RAW1 = dbMIC_RAW[which(substr(rownames(dbMIC_RAW),1,1) %in% sAREA),]

dbMIC_PERC=data.frame()

for(nMIC_ROW in 1:nrow(dbMIC_RAW1)){

dbMIC_PERC=rbind(dbMIC_PERC,(dbMIC_RAW1[nMIC_ROW,]/colSums(dbMIC_RAW1)))

}

dbMIC_RAW2=dbMIC_PERC

#Use a segmented regression to estimate the breakpoint of explained variation. Use this break point to select variables.

distMIC = vegdist(dbMIC_RAW2, "bray")

dbMIC_PCOA = pcoa(distMIC, correction="lingoes") #PCoA scores using Bray-Curtis dissimilarity, this can then be used in an RDA to create a distance based RDA

dfMIC_PCOA_LM = cbind(as.data.frame(dbMIC_PCOA$values[,'Rel_corr_eig']), as.data.frame(seq(1:length(dbMIC_PCOA$values[,'Rel_corr_eig']))))

colnames(dfMIC_PCOA_LM) = c('x', 'y')

lmMIC_PCOA = lm(x ~ y,data=dfMIC_PCOA_LM)

lmMIC_PCOA_SEG = segmented(lmMIC_PCOA, seg.Z=~y, psi=list(y=c(max(which(dbMIC_PCOA$values[,'Rel_corr_eig'] >0.05)))),control=seg.control(n.boot=10000))#probably a bit excessive, but it is to avoid deviation from the true break point (which can lead to drastically different results as seen on 16-01-2015 together with Amber)

nMIC_PCOA_POS = ceiling(lmMIC_PCOA_SEG$psi[2]) #round up the estimated break point to whole variables

dbMIC_PCOA=(dbMIC_PCOA$vectors.cor)[,1:nMIC_PCOA_POS]

colnames(dbMIC_PCOA) = paste("PCOA_VAR",seq(1,ncol(dbMIC_PCOA),le=ncol(dbMIC_PCOA)),"_",sAREA,sep="")#very important as otherwise there are no headers to select variables on for e.g. gradientForest function

rownames(dbMIC_PCOA) = rownames(dbMIC_RAW2)

vMIC_EXPLVAR = c(vMIC_EXPLVAR, dfMIC_PCOA_LM[1:nMIC_PCOA_POS,1])

dfPCOA_SAVE[which(rownames(dfPCOA_SAVE) %in% rownames(dbMIC_PCOA)),which(colnames(dfPCOA_SAVE) %in% colnames(dbMIC_PCOA))] = dbMIC_PCOA

}

dfPCOA_SAVE[is.na(dfPCOA_SAVE)]=0

if(length(which(colSums(dfPCOA_SAVE) == 0))>0){dfPCOA_SAVE = dfPCOA_SAVE[,-(which(colSums(dfPCOA_SAVE) == 0))]} #remove all zero columns

dbMIC_PCOA = dfPCOA_SAVE

#Vegetation

lHEAD_VEGPCOA=c()

for(sAREA in lAREAS){lHEAD_VEGPCOA = c(lHEAD_VEGPCOA, paste("PCOA_VAR",seq(1,23,le=23),"_",sAREA,sep=""))}

dfPCOA_SAVE=data.frame(matrix(NA,nrow(dbSPEC), length(lHEAD_VEGPCOA)))

colnames(dfPCOA_SAVE)=lHEAD_VEGPCOA

rownames(dfPCOA_SAVE) = rownames(dbSPEC)

for(sAREA in lAREAS){

dbSPEC1 = dbSPEC[which(substr(rownames(dbSPEC),1,1) %in% sAREA),]

distVEG = vegdist(dbSPEC1, "bray")

dbVEG_PCOA = pcoa(distVEG, correction="lingoes") #PCoA scores using Bray-Curtis dissimilarity, this can then be used in an RDA to create a distance based RDA

dfVEG_PCOA_LM = cbind(as.data.frame(dbVEG_PCOA$values[,'Rel_corr_eig']), as.data.frame(seq(1:length(dbVEG_PCOA$values[,'Rel_corr_eig']))))

colnames(dfVEG_PCOA_LM) = c('x', 'y')

lmVEG_PCOA = lm(x ~ y,data=dfVEG_PCOA_LM)

lmVEG_PCOA_SEG = segmented(lmVEG_PCOA, seg.Z=~y, psi=list(y=c(max(which(dbVEG_PCOA$values[,'Rel_corr_eig'] >0.05)))),control=seg.control(n.boot=10000))#probably a bit excessive, but it is to avoid deviation from the true break point (which can lead to drastically different results as seen on 16-01-2015 together with Amber)

nVEG_PCOA_POS = ceiling(lmVEG_PCOA_SEG$psi[2]) #round up the estimated break point to whole variables

dbVEG_PCOA=(dbVEG_PCOA$vectors.cor)[,1:nVEG_PCOA_POS]

colnames(dbVEG_PCOA) = paste("PCOA_VAR",seq(1,ncol(dbVEG_PCOA),le=ncol(dbVEG_PCOA)),"_",sAREA,sep="")#very important as otherwise there are no headers to select variables on for e.g. gradientForest function

rownames(dbVEG_PCOA) = rownames(dbSPEC1)

vVEG_EXPLVAR = c(vVEG_EXPLVAR, dfVEG_PCOA_LM[1:nVEG_PCOA_POS,1])

dfPCOA_SAVE[which(rownames(dfPCOA_SAVE) %in% rownames(dbVEG_PCOA)),which(colnames(dfPCOA_SAVE) %in% colnames(dbVEG_PCOA))] = dbVEG_PCOA

}

dfPCOA_SAVE[is.na(dfPCOA_SAVE)]=0

if(length(which(colSums(dfPCOA_SAVE) == 0))>0){dfPCOA_SAVE = dfPCOA_SAVE[,-(which(colSums(dfPCOA_SAVE) == 0))]} #remove all zero columns

dbVEG_PCOA = dfPCOA_SAVE

#--within areas MEM--

dfMEMMIC=data.frame(matrix(0,nrow(dfAREA_SEL),40))

rownames(dfMEMMIC)=rownames(dfAREA_SEL)

lMEM_HEAD=c()

for(sAREA in lAREAS){

dbXY = subset(dbXY_RAW[c(4,5)], grepl(25, row.names(dbXY_RAW)) != TRUE )#remove site 25 (if it exists)

dbXY = dbXY[which(row.names(dbXY) %in% row.names(dbMIC_PCOA)),]

dbXY = subset(dbXY, substr(row.names(dbXY), 1, 1) %in% sAREA) #Subselection of specific area based on area letter in lAREA

#select subset of XY by area and define distance related variables

mXY = as.matrix(dbXY) #XY coordinates

mDISTXY = dist(dbXY) #Distance matrix

#non-cut down MEMS

mDISTXY2=1 - (mDISTXY/max(mDISTXY))

lwDIST = mat2listw(as.matrix(mDISTXY2))

mMEMSPEC = scores.listw(lwDIST, echo=TRUE)

vMEMSPEC = mMEMSPEC$vectors

vMORANI = test.scores(mMEMSPEC, lwDIST, 1000)

# Save MEMs with positive spatial correlation that are significant

vMORANI_POS = which(vMORANI[,1] > -1/(nrow(vMEMSPEC)-1))

vMEM_USE = as.data.frame(vMEMSPEC[,vMORANI_POS])

colnames(vMEM_USE) = paste("MEM_VAR_",sAREA,"_",seq(1,ncol(vMEM_USE),le=ncol(vMEM_USE)),sep="")

nMEM_MINCOL = min(which(colSums(dfMEMMIC)==0))

if(is.infinite(nMEM_MINCOL)==TRUE){nMEM_MINCOL=1}

dfMEMMIC[which(grepl(sAREA, rownames(dfMEMMIC))==1),c(nMEM_MINCOL:(nMEM_MINCOL+ncol(vMEM_USE)-1))] = vMEM_USE

lMEM_HEAD = c(lMEM_HEAD,colnames(vMEM_USE))

}

dfMEMMIC=dfMEMMIC[,which(colSums(dfMEMMIC)!=0)]

colnames(dfMEMMIC)=lMEM_HEAD

dfSPACE_WORK = cbind.data.frame(dfMEMMIC)

dfSPACE_WORK_FS = cbind.data.frame(dfMEMMIC)

#-----------------------------------

#---GRADIENT FOREST ANALYSIS--------

#___________________________________

#Select variables using GF (VI <0.001)

#Use these variables in an RDA and varpart (no FS)

#then use a gradient forest to identify species most affected by Env, Man and space

##---GRADIENT FOREST

###run for each area seperately

##dfIMP_STORE = data.frame(matrix(NA,ncol(cbind(dfENV_WORK_FS, dfSPACE_WORK_FS, dfLU_WORK, dbSPEC)),0))

##for(sAREA in lAREAS){

## dfPREDICT =cbind(dfENV_WORK_FS[which(substr(rownames(dfENV_WORK_FS),1,1) %in% sAREA),], dfSPACE_WORK_FS[which(substr(rownames(dfENV_WORK_FS),1,1) %in% sAREA),], dfLU_WORK[which(substr(rownames(dfENV_WORK_FS),1,1) %in% sAREA),], dbSPEC[which(substr(rownames(dbSPEC),1,1) %in% sAREA),])#dfVEG_WORK) #untransformed vegetation data goes in here

## #dfPREDICT = dfPREDICT[,-which((colSums(dfPREDICT==0)>nrow(dfPREDICT)-1) | (colSums(dfPREDICT==1)>nrow(dfPREDICT)-1))] #remove values with no variation (e.g. dummies that are all 0/1)

## #select microbial community

## #--scaled per plfa, raw data

## dbMIC_PCOA2 = dbMIC_PCOA[which(substr(rownames(dbMIC_PCOA),1,1) %in% sAREA),]

##

## gf_COMM= gradientForest(cbind(dfPREDICT, dbMIC_PCOA2), predictor.vars = colnames(dfPREDICT), response.vars = colnames(dbMIC_PCOA2), ntree = 999, mtry=ceiling(sqrt(ncol(dfPREDICT))), maxLevel=floor(log2(nrow(dfPREDICT) * 0.368/2)), corr.threshold=0.5)

## if(is.null(gf_COMM)==TRUE){

## vIMP = rep(0, nrow(dfIMP_STORE))

## }else{

## vIMP = importance(gf_COMM, type="W")

## }

## dfIMP_STORE = cbind(dfIMP_STORE, vIMP)

##}

##colnames(dfIMP_STORE)=lAREAS

##

##vIMP = rowSums(dfIMP_STORE)/ncol(dfIMP_STORE)

##

#run for all data

dfPREDICT =cbind(dfENV_WORK_FS, dfSPACE_WORK_FS, dfLU_WORK, dbVEG_PCOA)

gf_COMM= gradientForest(cbind(dfPREDICT, dbMIC_PCOA), predictor.vars = colnames(dfPREDICT), response.vars = colnames(dbMIC_PCOA), ntree = 9999, mtry=ceiling(sqrt(ncol(dfPREDICT))), maxLevel=floor(log2(nrow(dfPREDICT) * 0.368/2)), corr.threshold=0.5)

vIMP = importance(gf_COMM, type="W")

#Idea: first run GF then RDA

#Select variables from GF with VI higher than 0.001 and use these in varpart without forsel

lVARS_GF_SEL = names(vIMP[which(vIMP>0.001)])

dfENV_SEL_GF = dfENV_WORK_FS[,which(colnames(dfENV_WORK_FS) %in% lVARS_GF_SEL),drop=F]

dfLU_SEL_GF = dfLU_WORK[,which(colnames(dfLU_WORK) %in% lVARS_GF_SEL),drop=F]

dfSPACE_SEL_GF = dfSPACE_WORK_FS[,which(colnames(dfSPACE_WORK_FS) %in% lVARS_GF_SEL), drop=F]

dfVEG_SEL_GF = dbVEG_PCOA[,which(colnames(dbVEG_PCOA) %in% lVARS_GF_SEL), drop=F]

#dfVEG_SEL_GF1 = dbSPEC[,which(colnames(dbSPEC) %in% lVARS_GF_SEL), drop=F]

#dfVEG_SEL_GF = decostand(dfVEG_SEL_GF1, "hellinger")

#-----------------------------------

#---RDA ANALYSIS--------------------

#___________________________________

#IDEA: create euclidean/bc distance matrices for the env/LU/VEG vars and use these in RDA instead of actual data

#then plot mean env-heterogeneity and mean spatial distance between sites

##RDA for between region differences

nPERM=99999

#Create formula's for RDA

sENV = ""

for(sVAR in colnames(dfENV_SEL_GF)){

if(sENV == ""){

sENV = sVAR

}else if(sENV != ""){

sENV = paste(sENV, sVAR, sep="+")

}

}

sSPACE = ""

for(sVAR in colnames(dfSPACE_SEL_GF)){

if(sSPACE == ""){

sSPACE = sVAR

}else if(sSPACE != ""){

sSPACE = paste(sSPACE, sVAR, sep="+")

}

}

sLU = ""

for(sVAR in colnames(dfLU_SEL_GF)){

if(sLU == ""){

sLU = sVAR

}else if(sLU != ""){

sLU = paste(sLU, sVAR, sep="+")

}

}

sVEG = ""

for(sVAR in colnames(dfVEG_SEL_GF)){

if(sVEG == ""){

sVEG = sVAR

}else if(sVEG != ""){

sVEG = paste(sVEG, sVAR, sep="+")

}

}

sAREA_FORM = paste(" + ", "Condition(",colnames(dfAREA_SEL[,4,drop=F]),")", sep="")

fRDA_ENV = as.formula(paste('dbMIC_PCOA',' ~ ',sENV,sep=""))

fRDA_SPACE = as.formula(paste('dbMIC_PCOA',' ~ ',sSPACE,sep=""))

fRDA_LU = as.formula(paste('dbMIC_PCOA',' ~ ',sLU,sep=""))

fRDA_VEG = as.formula(paste('dbMIC_PCOA',' ~ ',sVEG,sep=""))

#--ENVIRONMENTAL+SOIL--

dfVEG_WORK =dfVEG_SEL_GF

rdaSPEC_ENV = rda(fRDA_ENV, data=cbind(dfENV_SEL_GF,dfAREA_SEL))

fR2_SPEC_ENV=RsquareAdj(rdaSPEC_ENV)$adj.r.squared #extract the adjusted R2

testSPEC_ENV=anova(rdaSPEC_ENV,permutations=nPERM)

# anova(rdaSPEC_ENV,permutations =nPERM,by='term')

fFSTAT_SPEC_ENV=testSPEC_ENV$F[1] #extract the F statistic

fPSTAT_SPEC_ENV=testSPEC_ENV$Pr[1] #extract the p value

fsRDA_ENV = try(forward.sel(dbMIC_PCOA, dfENV_SEL_GF, nperm=nPERM,adjR2thresh=fR2_SPEC_ENV))

if(class(fsRDA_ENV)=="try-error"| fPSTAT_SPEC_ENV >0.05){lENV_FS = c()}else{lENV_FS = c(unlist(as.data.frame(fsRDA_ENV[,2])))}

#--SPATIAL--

rdaSPEC_MEM = rda(fRDA_SPACE, data=cbind(dfSPACE_SEL_GF,dfAREA_SEL))

fR2_SPEC_MEM=RsquareAdj(rdaSPEC_MEM)$adj.r.squared #extract the adjusted R2

testSPEC_MEM=anova(rdaSPEC_MEM,permutations=nPERM)

# anova(rdaSPEC_MEM,permutations =nPERM,by='term')

fFSTAT_SPEC_MEM=testSPEC_MEM$F[1] #extract the F statistic

fPSTAT_SPEC_MEM=testSPEC_MEM$Pr[1] #extract the p value

fsRDA_MEM = try(forward.sel(dbMIC_PCOA, dfSPACE_SEL_GF, nperm=nPERM,adjR2thresh=fR2_SPEC_MEM))

if(class(fsRDA_MEM)=="try-error"| fPSTAT_SPEC_MEM >0.05){lMEM_FS = c()}else{lMEM_FS = c(unlist(as.data.frame(fsRDA_MEM[,2])))}

#--MANAGEMENT--

rdaSPEC_MAN = rda(fRDA_LU, data=cbind(dfLU_SEL_GF,dfAREA_SEL))

fR2_SPEC_MAN=RsquareAdj(rdaSPEC_MAN)$adj.r.squared #extract the adjusted R2

testSPEC_MAN=anova(rdaSPEC_MAN,permutations =nPERM)

# anova(rdaSPEC_MAN,permutations =nPERM,by='term')

fFSTAT_SPEC_MAN=testSPEC_MAN$F[1] #extract the F statistic

fPSTAT_SPEC_MAN=testSPEC_MAN$Pr[1] #extract the p value

fsRDA_MAN = try(forward.sel(dbMIC_PCOA, dfLU_SEL_GF, nperm=nPERM,adjR2thresh=fR2_SPEC_MAN))

if(class(fsRDA_MAN)=="try-error"| fPSTAT_SPEC_MAN >0.05){lMAN_FS = c()}else{lMAN_FS = c(unlist(as.data.frame(fsRDA_MAN[,2])))}

#--VEGETATION--

#rdaSPEC_VEG = rda(dbMIC_PCOA~., as.data.frame(dbVEG_PCOA))

rdaSPEC_VEG = rda(fRDA_VEG, data=cbind(dfVEG_SEL_GF,dfAREA_SEL))

fR2_SPEC_VEG=RsquareAdj(rdaSPEC_VEG)$adj.r.squared #extract the adjusted R2

testSPEC_VEG=anova(rdaSPEC_VEG,permutations =nPERM)

# anova(rdaSPEC_VEG,permutations =nPERM,by='term')

fFSTAT_SPEC_VEG=testSPEC_VEG$F[1] #extract the F statistic

fPSTAT_SPEC_VEG=testSPEC_VEG$Pr[1] #extract the p value

#fsRDA_VEG = try(forward.sel(dbMIC_PCOA, dfVEG_SEL_GF, nperm=nPERM,adjR2thresh=fR2_SPEC_VEG))

fsRDA_VEG = try(forward.sel(dbMIC_PCOA, dfVEG_WORK, nperm=nPERM,adjR2thresh=fR2_SPEC_VEG))

if(class(fsRDA_VEG)=="try-error"| fPSTAT_SPEC_VEG >0.05){lVEG_FS = c()}else{lVEG_FS = c(unlist(as.data.frame(fsRDA_VEG[,2])))}

#--COMBINED MODEL---

dfALL_WORK_FS = cbind.data.frame(dfENV_SEL_GF[,lENV_FS,drop=F], dfSPACE_SEL_GF[,lMEM_FS, drop=F], dfLU_SEL_GF[,lMAN_FS,drop=F], dfVEG_WORK[,lVEG_FS,drop=F])

#dfALL_WORK_FS = cbind.data.frame(dfENV_SEL_GF, dfSPACE_SEL_GF, dfLU_SEL_GF, dfVEG_SEL_GF)

#Create a total model

rdaSPEC_COMB = rda(dbMIC_PCOA~., dfALL_WORK_FS)

fR2_SPEC_COMB=RsquareAdj(rdaSPEC_COMB)$adj.r.squared #extract the adjusted R2

testSPEC_COMB=anova(rdaSPEC_COMB,permutations=nPERM)

#anova(rdaSPEC_COMB,permutations=nPERM, by='term')

fFSTAT_SPEC_COMB=testSPEC_COMB$F[1] #extract the F statistic

fPSTAT_SPEC_COMB=testSPEC_COMB$Pr[1] #extract the p value

#forward.sel(dbMIC_PCOA, dfALL_WORK_FS, nperm=nPERM,adjR2thresh=fR2_SPEC_COMB)

#---VAR PART---

#vartpart plot

pdf(paste("C:/Users/Sven/Documents/PhD/R/Data/RESULTS_MIC/","VARPART_WITHIN_POLDERS",".pdf",sep=""),width=10, height=7, pointsize=14)

if(length(lENV_FS)>0 & length(lMEM_FS)>0 & length(lMAN_FS)>0 & length(lVEG_FS)>0){

lPART = c('ENV', 'SPACE', 'FERTILIZATION', 'VEG')

plot(varpart(dbMIC_PCOA, dfENV_SEL_GF[,lENV_FS,drop=F], dfSPACE_SEL_GF[,lMEM_FS, drop=F], dfLU_SEL_GF[,lMAN_FS,drop=F], dfVEG_WORK[,lVEG_FS,drop=F]), Xnames=lPART)

}else if(length(lENV_FS)>0 & length(lMEM_FS)>0 & length(lMAN_FS)>0 ){

lPART = c('ENV', 'SPACE', 'FERTILIZATION')

plot(varpart(dbMIC_PCOA, dfENV_SEL_GF[,lENV_FS,drop=F], dfSPACE_SEL_GF[,lMEM_FS, drop=F], dfLU_SEL_GF[,lMAN_FS,drop=F]), Xnames=lPART)

}else if(length(lENV_FS)>0 & length(lMEM_FS)>0 & length(lVEG_FS)>0){

lPART = c('ENV', 'SPACE', 'VEG')

plot(varpart(dbMIC_PCOA, dfENV_SEL_GF[,lENV_FS,drop=F], dfSPACE_SEL_GF[,lMEM_FS, drop=F], dfVEG_WORK[,lVEG_FS,drop=F]), Xnames=lPART)

}else if(length(lENV_FS)>0 & length(lMAN_FS)>0 & length(lVEG_FS)>0){

lPART = c('ENV', 'FERTILIZATION', 'VEG')

plot(varpart(dbMIC_PCOA, dfENV_SEL_GF[,lENV_FS,drop=F], dfLU_SEL_GF[,lMAN_FS,drop=F], dfVEG_WORK[,lVEG_FS,drop=F]), Xnames=lPART)

}else if(length(lENV_FS)>0 & length(lMEM_FS)>0 & length(lMAN_FS)>0 & length(lVEG_FS)>0){

lPART = c('SPACE', 'FERTILIZATION', 'VEG')

plot(varpart(dbMIC_PCOA, dfSPACE_SEL_GF[,lMEM_FS, drop=F], dfLU_SEL_GF[,lMAN_FS,drop=F], dfVEG_WORK[,lVEG_FS,drop=F]), Xnames=lPART)

}else if(length(lENV_FS)>0 & length(lMEM_FS)>0 & length(lMAN_FS)>0 & length(lVEG_FS)>0){

lPART = c('ENV', 'SPACE')

plot(varpart(dbMIC_PCOA, dfENV_SEL_GF[,lENV_FS,drop=F], dfSPACE_SEL_GF[,lMEM_FS, drop=F]), Xnames=lPART)

}else if(length(lENV_FS)>0 & length(lVEG_FS)>0){

lPART = c('ENV', 'VEG')

plot(varpart(dbMIC_PCOA, dfENV_SEL_GF[,lENV_FS,drop=F], dfVEG_WORK[,lVEG_FS,drop=F]), Xnames=lPART)

}else if(length(lENV_FS)>0 & length(lMAN_FS)>0){

lPART = c('ENV', 'FERTILIZATION')

plot(varpart(dbMIC_PCOA, dfENV_SEL_GF[,lENV_FS,drop=F], dfLU_SEL_GF[,lMAN_FS,drop=F]), Xnames=lPART)

}else if(length(lMEM_FS)>0 & length(lVEG_FS)>0){

lPART = c('SPACE', 'VEG')

plot(varpart(dbMIC_PCOA, dfSPACE_SEL_GF[,lMEM_FS, drop=F], dfVEG_WORK[,lVEG_FS,drop=F]), Xnames=lPART)

}else if(length(lMEM_FS)>0 & length(lMAN_FS)>0){

lPART = c('SPACE', 'FERTILIZATION')

plot(varpart(dbMIC_PCOA, dfSPACE_SEL_GF[,lMEM_FS, drop=F], dfLU_SEL_GF[,lMAN_FS,drop=F]), Xnames=lPART)

}else if(length(lMAN_FS)>0 & length(lVEG_FS)>0){

lPART = c('FERTILIZATION', 'VEG')

plot(varpart(dbMIC_PCOA, dfLU_SEL_GF[,lMAN_FS,drop=F], dfVEG_WORK[,lVEG_FS,drop=F]), Xnames=lPART)

}else{

print('no variation partitioning possible')

}

dev.off()

##lPART = c('ENV', 'SPACE', 'MANAG', 'VEG')

##windows()

##plot(varpart(dbMIC_PCOA, dfENV_SEL_GF[,lENV_FS,drop=F], dfSPACE_SEL_GF[,lMEM_FS, drop=F], dfLU_SEL_GF_FS[,lMAN_FS,drop=F], dbVEG_PCOA[,lVEG_FS,drop=F]), Xnames=lPART)

##plot(varpart(dbMIC_PCOA, dfENV_SEL_GF, dfSPACE_SEL_GF, dfLU_SEL_GF, dbVEG_PCOA), Xnames=lPART)

##

##windows()

##lPART = c('SPACE', 'VEG')

##plot(varpart(dbMIC_PCOA, dfSPACE_SEL_GF[,lMEM_FS,drop=F], dbVEG_PCOA[,lVEG_FS,drop=F]), Xnames=lPART)

###plot(varpart(dbMIC_PCOA, dfENV_SEL_GF, dfSPACE_SEL_GF, dfVEG_SEL_GF), Xnames=lPART)

#--forward selected (full) and partial models (marginal partitions)

dfOUTPUT_RDA = data.frame(matrix(NA,0,5))

lMODELS_RDA = c('FULL_ENV', 'PURE_ENV','FULL_SPACE','PURE_SPACE','FULL_FERTILIZATION','PURE_FERTILIZATION','FULL_VEGETATION','PURE_VEGETATION')

##if(length(lENV_FS)>0){lMODELS_RDA = c(lMODELS_RDA, 'PURE_ENV')}

##if(length(lMEM_FS)>0){lMODELS_RDA = c(lMODELS_RDA, 'PURE_SPACE')}

##if(length(lMAN_FS)>0){lMODELS_RDA = c(lMODELS_RDA, 'PURE_FERTILIZATION')}

##if(length(lVEG_FS)>0){lMODELS_RDA = c(lMODELS_RDA, 'PURE_VEGETATION')}

for(sMODEL_RDA in lMODELS_RDA){

if(sMODEL_RDA == 'PURE_ENV'){dfEXPL = dfENV_SEL_GF[,lENV_FS,drop=F]

dfCONDITION = dfALL_WORK_FS[,-which(colnames(dfALL_WORK_FS) %in% colnames(dfEXPL))]}

if(sMODEL_RDA == 'PURE_SPACE'){dfEXPL = dfSPACE_SEL_GF[,lMEM_FS, drop=F]

dfCONDITION = dfALL_WORK_FS[,-which(colnames(dfALL_WORK_FS) %in% colnames(dfEXPL))]}

if(sMODEL_RDA == 'PURE_FERTILIZATION'){dfEXPL = dfLU_SEL_GF[,lMAN_FS,drop=F]

dfCONDITION = dfALL_WORK_FS[,-which(colnames(dfALL_WORK_FS) %in% colnames(dfEXPL))]}

if(sMODEL_RDA == 'PURE_VEGETATION'){dfEXPL = dbVEG_PCOA[,lVEG_FS,drop=F]

dfCONDITION = dfALL_WORK_FS[,-which(colnames(dfALL_WORK_FS) %in% colnames(dfEXPL))]}

if(sMODEL_RDA == 'FULL_ENV'){dfEXPL = dfENV_SEL_GF[,lENV_FS,drop=F]

dfCONDITION = data.frame(matrix(0,nrow(dfALL_WORK_FS),0))}

if(sMODEL_RDA == 'FULL_SPACE'){dfEXPL = dfSPACE_SEL_GF[,lMEM_FS, drop=F]

dfCONDITION = data.frame(matrix(0,nrow(dfALL_WORK_FS),0))}

if(sMODEL_RDA == 'FULL_FERTILIZATION'){dfEXPL = dfLU_SEL_GF[,lMAN_FS,drop=F]

dfCONDITION = data.frame(matrix(0,nrow(dfALL_WORK_FS),0))}

if(sMODEL_RDA == 'FULL_VEGETATION'){dfEXPL = dbVEG_PCOA[,lVEG_FS,drop=F]

dfCONDITION = data.frame(matrix(0,nrow(dfALL_WORK_FS),0))}

if(ncol(dfCONDITION) >0 & ncol(dfEXPL)>0){

rdaSPEC_PART = rda(dbMIC_PCOA, dfEXPL, dfCONDITION)

fR2_SPEC_PART=RsquareAdj(rdaSPEC_PART)$adj.r.squared #extract the adjusted R2

testSPEC_PART=anova(rdaSPEC_PART,permutations=nPERM)

fFSTAT_SPEC_PART=testSPEC_PART$F[1] #extract the F statistic

fPSTAT_SPEC_PART=testSPEC_PART$Pr[1] #extract the p value

sFS_VARS = paste(colnames(dfEXPL),collapse = "; ")

}else if(substr(sMODEL_RDA,1,4)=='FULL' & ncol(dfEXPL)>0){

rdaSPEC_PART = rda(dbMIC_PCOA, dfEXPL, dfCONDITION)

fR2_SPEC_PART=RsquareAdj(rdaSPEC_PART)$adj.r.squared #extract the adjusted R2

testSPEC_PART=anova(rdaSPEC_PART,permutations=nPERM)

fFSTAT_SPEC_PART=testSPEC_PART$F[1] #extract the F statistic

fPSTAT_SPEC_PART=testSPEC_PART$Pr[1] #extract the p value

sFS_VARS = paste(colnames(dfEXPL),collapse = "; ")

}else{

fR2_SPEC_PART=0

fFSTAT_SPEC_PART=0

fPSTAT_SPEC_PART=0

sFS_VARS = ""

}

dfOUT1 = t(as.data.frame(c(sMODEL_RDA, fR2_SPEC_PART,fFSTAT_SPEC_PART,fPSTAT_SPEC_PART,sFS_VARS)))

dfOUTPUT_RDA=rbind(dfOUTPUT_RDA,dfOUT1)

}

colnames(dfOUTPUT_RDA) = c('MODEL','adR2', 'F_val', 'p_val', 'FS_Variables')

write.table(dfOUTPUT_RDA,paste("C:/Users/Sven/Documents/PhD/R/Data/RESULTS_MIC/","VARPART_WITHIN_POLDERS",".csv",sep=""), sep=',')

###---RAW data variant

###run a gradient forest to determine variable importance

##dfPREDICT1 = cbind.data.frame(dfENV_WORK_FS, dfSPACE_WORK_FS, dfLU_WORK_FS, as.data.frame(dbSPEC))

###Do so for every model type seperately (ENV, MAN, SPACE, VEG)

##lMODEL = c('ENV', 'SPACE', 'FERTILIZATION', 'VEG')

##dfIMP_STORE=data.frame(matrix(NA, ncol(dfPREDICT1), ncol(dbMIC_RAW)))

##colnames(dfIMP_STORE) = colnames(dbMIC_RAW)

##rownames(dfIMP_STORE) = colnames(dfPREDICT1)

##for(sAREA in lAREAS){

## for(sMODEL in lMODEL){

## if(sMODEL == 'ENV'){dfPREDICT = dfENV_WORK_FS[which(substr(rownames(dfENV_WORK_FS),1,1) %in% sAREA),]}

## if(sMODEL == 'SPACE'){dfPREDICT = dfSPACE_WORK_FS[which(substr(rownames(dfENV_WORK_FS),1,1) %in% sAREA),]}

## if(sMODEL == 'FERTILIZATION'){dfPREDICT = dfLU_WORK_FS[which(substr(rownames(dfENV_WORK_FS),1,1) %in% sAREA),]}

## if(sMODEL == 'VEG'){dfPREDICT = as.data.frame(dbSPEC)[which(substr(rownames(dfENV_WORK_FS),1,1) %in% sAREA),]}

## dfPREDICT = dfPREDICT[,which((colSums(dfPREDICT==0)<nrow(dfPREDICT)-1))] #remove values with no variation (e.g. dummies that are all 0/1)

##

## gf_SPEC = gradientForest(cbind(dfPREDICT, dbMIC_RAW[which(substr(rownames(dfENV_WORK_FS),1,1) %in% sAREA),]), predictor.vars = colnames(dfPREDICT), response.vars = colnames(dbMIC_RAW), ntree = 999, mtry=ceiling(sqrt(ncol(dfPREDICT))), maxLevel=floor(log2(nrow(dfPREDICT) * 0.368/2)), corr.threshold=0.5)

## if(is.null(gf_SPEC)==FALSE){

## (vIMP = importance(gf_SPEC, type="W"))

## dfIMP_STORE_MODEL = as.data.frame(gf_SPEC$imp.rsq)

## }else{

## dfIMP_STORE_MODEL=data.frame(matrix(0,ncol(dfPREDICT), ncol(dbMIC_RAW)))

## colnames(dfIMP_STORE_MODEL) = colnames(dbMIC_RAW)

## rownames(dfIMP_STORE_MODEL) = colnames(dfPREDICT)

## }

## dfIMP_STORE[which(rownames(dfIMP_STORE) %in% rownames(dfIMP_STORE_MODEL)),which(colnames(dfIMP_STORE) %in% colnames(dfIMP_STORE_MODEL))] = dfIMP_STORE_MODEL

## }

##}

##dfIMP_STORE[is.na(dfIMP_STORE)]=0

##rowSums(dfIMP_STORE)[which(rowSums(dfIMP_STORE)>0.0)]

#---PCOA variant

#run a gradient forest to determine variable importance

#Define dbSPEC data with discriminating abilities (not all zero, or 1 etc.)

dfSPEC_DISC=data.frame(matrix(NA,nrow(dbSPEC),0))

for(nSPEC_DISC in 1:ncol(dbSPEC)){

if(length(unique(dbSPEC[,nSPEC_DISC])) >2){

dfSPEC_DISC=cbind.data.frame(dfSPEC_DISC, dbSPEC[,nSPEC_DISC,drop=F])

}

}

dfPREDICT1 = cbind.data.frame(dfENV_WORK_FS, dfSPACE_WORK_FS, dfLU_WORK_FS, dfVEG_SEL_GF, dfSPEC_DISC)

#Do so for every model type seperately (ENV, MAN, SPACE, VEG)

lMODEL = c('ENV', 'SPACE', 'FERTILIZATION', 'VEG', 'VEG_SPECS')

dfIMP_STORE=data.frame(matrix(NA, ncol(dfPREDICT1), ncol(dbMIC_PCOA)))

colnames(dfIMP_STORE) = colnames(dbMIC_PCOA)

rownames(dfIMP_STORE) = colnames(dfPREDICT1)

#make data frames expressing explained variation of different PCOA axes

dfMIC_EXPLVAR=t(as.data.frame(rep(as.data.frame(vMIC_EXPLVAR/sum(vMIC_EXPLVAR)), nrow(dfIMP_STORE))))

colnames(dfMIC_EXPLVAR) = colnames(dbMIC_PCOA)

rownames(dfMIC_EXPLVAR) = colnames(dfPREDICT1)

dfVEG_EXPLVAR=as.data.frame(rep(as.data.frame(vVEG_EXPLVAR[which(colnames(dbVEG_PCOA) %in% lVARS_GF_SEL)]/sum(vVEG_EXPLVAR[which(colnames(dbVEG_PCOA) %in% lVARS_GF_SEL)])), ncol(dfIMP_STORE)))

colnames(dfVEG_EXPLVAR) = colnames(dbMIC_PCOA)

rownames(dfVEG_EXPLVAR) = colnames(dfVEG_SEL_GF)

for(sAREA in lAREAS){

for(sMODEL in lMODEL){

if(sMODEL == 'ENV'){dfPREDICT = dfENV_WORK_FS[which(substr(rownames(dfENV_WORK_FS),1,1) %in% sAREA),]}

if(sMODEL == 'SPACE'){dfPREDICT = dfSPACE_WORK_FS[which(substr(rownames(dfENV_WORK_FS),1,1) %in% sAREA),]}

if(sMODEL == 'FERTILIZATION'){dfPREDICT = dfLU_WORK_FS[which(substr(rownames(dfENV_WORK_FS),1,1) %in% sAREA),]}

if(sMODEL == 'VEG'){dfPREDICT = as.data.frame(dbVEG_PCOA)[which(substr(rownames(dfENV_WORK_FS),1,1) %in% sAREA),]}

if(sMODEL == 'VEG_SPECS'){dfPREDICT = dfSPEC_DISC[which(substr(rownames(dfENV_WORK_FS),1,1) %in% sAREA),]}

dfPREDICT = dfPREDICT[,which((colSums(dfPREDICT==0)<nrow(dfPREDICT)-1))] #remove values with no variation (e.g. dummies that are all 0/1)

gf_SPEC = gradientForest(cbind(dfPREDICT, dbMIC_PCOA[which(substr(rownames(dfENV_WORK_FS),1,1) %in% sAREA),]), predictor.vars = colnames(dfPREDICT), response.vars = colnames(dbMIC_PCOA), ntree = 9999, mtry=ceiling(sqrt(ncol(dfPREDICT))), maxLevel=floor(log2(nrow(dfPREDICT) * 0.368/2)), corr.threshold=0.5)

if(is.null(gf_SPEC)==FALSE){

(vIMP = importance(gf_SPEC, type="W"))

dfIMP_STORE_MODEL = as.data.frame(gf_SPEC$imp.rsq)

}else{

dfIMP_STORE_MODEL=data.frame(matrix(0,ncol(dfPREDICT), ncol(dbMIC_PCOA)))

colnames(dfIMP_STORE_MODEL) = colnames(dbMIC_PCOA)

rownames(dfIMP_STORE_MODEL) = colnames(dfPREDICT)

}

dfIMP_STORE[which(rownames(dfIMP_STORE) %in% rownames(dfIMP_STORE_MODEL)),which(colnames(dfIMP_STORE) %in% colnames(dfIMP_STORE_MODEL))] = dfIMP_STORE_MODEL

}

}

dfIMP_STORE[is.na(dfIMP_STORE)]=0

dfIMP_STORE=dfIMP_STORE * dfMIC_EXPLVAR

dfIMP_STORE[which(rownames(dfIMP_STORE) %in% rownames(dfVEG_EXPLVAR)),which(colnames(dfIMP_STORE) %in% colnames(dfVEG_EXPLVAR))] = dfVEG_EXPLVAR * dfIMP_STORE[which(rownames(dfIMP_STORE) %in% rownames(dfVEG_EXPLVAR)),which(colnames(dfIMP_STORE) %in% colnames(dfVEG_EXPLVAR))]

write.table(dfIMP_STORE, paste("C:/Users/Sven/Documents/PhD/R/Data/RESULTS_MIC/","GF_VIMP_WITHIN_POLDERS",".csv",sep=""), sep=',')

#rowSums(dfIMP_STORE)[which(rowSums(dfIMP_STORE)>0.0)]

#------------------------------------------------------------

#------------------------------------------------------------

#------------------BIOLOG DATA-------------------------------

#------------------------------------------------------------

#------------------------------------------------------------

#_____________

#Load data

dbSPEC_RAW = read.csv("C:/Users/Sven/Documents/PhD/Data/Veg 2014/20150803_VEG_CONVERSION.csv", header=TRUE, sep=",", dec = ".", row.names=1, stringsAsFactors=FALSE)

dbMIC_RAW = read.csv("C:/Users/Sven/Documents/PhD/Data/Microbial/20150805_BIOLOG.csv", header=TRUE, sep=",", dec = ".", row.names=1, stringsAsFactors=FALSE)

dbENV_RAW = read.csv("C:/Users/Sven/Documents/PhD/Data/Env/20150406_ENV_VEG.csv", header=TRUE, sep=",", dec = ".", row.names=1)

dbLU_RAW = read.csv('C:/Users/Sven/Documents/PhD/Data/LandUse/201508_FERT_ALL_MICROBIAL.csv', header=TRUE, sep=",", dec = ".", row.names=1, stringsAsFactors=TRUE)

dbXY_RAW = read.table("C:/Users/Sven/Documents/PhD/Data/Location_20121314.txt", header=TRUE, sep=" ", row.names=1)

dbAREA_RAW = read.csv("C:/Users/Sven/Documents/PhD/Data/AreaDummies.csv", header=TRUE, sep=",", dec = ".", row.names=1)

dbSOIL_RAW = read.csv("C:/Users/Sven/Documents/PhD/Data/SoilType/20141202_SoilType_BOFEKPAWN.csv", header=TRUE, sep=",", dec = ".", row.names=1)

#Sort and transform the biolog data

lMIC_SEL = c('CODE', 'Area', 'Site', 'Timepoint', 'Replicate', 'Wavelength','A1') #A1 is blank, already corrected for by subtraction from each value

lTIME_SEL = c(1,2,3,4)

lAREAS = c('Q','R','S','T','U','Z')

dfMIC_590 = subset(dbMIC_RAW, dbMIC_RAW$Wavelength == 590 & dbMIC_RAW$Area != 'P' & dbMIC_RAW$Timepoint %in% lTIME_SEL)

dfMIC_750 = subset(dbMIC_RAW, dbMIC_RAW$Wavelength == 750 & dbMIC_RAW$Area != 'P' & dbMIC_RAW$Timepoint %in% lTIME_SEL)

#subtract 750nm non-specific absorbance from the actual absorbance signal

dfMIC_CORR1 = dfMIC_590[,-which(colnames(dfMIC_590) %in% lMIC_SEL)]-dfMIC_750[,-which(colnames(dfMIC_750) %in% lMIC_SEL)]

dfMIC_CORR1[dfMIC_CORR1<(mean(data.matrix(dfMIC_750[,-which(colnames(dfMIC_750) %in% lMIC_SEL)]))/2)] = 0

dfMIC_CORR2 = cbind.data.frame(dfMIC_590[,which(colnames(dfMIC_590) %in% lMIC_SEL)], dfMIC_CORR1)

lSITES_SEL = unique(dfMIC_CORR2$CODE)

#combine technical reps

dfMIC_CORR3 = aggregate(dfMIC_CORR2[, -c(1:6)], list(dfMIC_CORR2$Area, dfMIC_CORR2$Site, dfMIC_CORR2$Timepoint), mean)

colnames(dfMIC_CORR3) = c(colnames(dfMIC_CORR2[,2:4]),colnames(dfMIC_CORR3[,4:ncol(dfMIC_CORR3)]))

#Selection of subset of vegetation based on Ellenberg values

remove(dbSPEC)

nLOW = 4 #Lower bound ellenberg value

nUP = 10 #Upper bound ellenberg value

#build a dataframe of species data within a given ellenberg range

for(r in 1: ncol(dbSPEC_RAW)){

if(as.numeric(dbSPEC_RAW[c(2),c(r)]) <= max(nUP,nLOW) && as.numeric(dbSPEC_RAW[c(2),c(r)]) >= min(nLOW,nUP) ){

if(exists("dbSPEC")){

dbSPEC = cbind(dbSPEC, dbSPEC_RAW[c(r)])

}else{

dbSPEC = dbSPEC_RAW[c(r)]

}

}

}

dbSPEC = data.matrix(dbSPEC[-c(1,2),])

dbSPEC = subset(dbSPEC, grepl(25, row.names(dbSPEC)) != TRUE )#remove site 25, main waterway site (if it exists) #not relevant in this case

dbSPEC = subset(dbSPEC, substr(row.names(dbSPEC), 1, 1) %in% lAREAS & row.names(dbSPEC) %in% lSITES_SEL)

if(length(which(colSums(dbSPEC) == 0))>0){dbSPEC = dbSPEC[,-(which(colSums(dbSPEC) == 0))]} #remove all zero columns

if(length(which(rowSums(dbSPEC) == 0))>0){dbSPEC = dbSPEC[-(which(rowSums(dbSPEC) == 0)),]} #remove all zero rows

#Define dbSPEC data with discriminating abilities (not all zero, or 1 etc.)

dfSPEC_DISC=data.frame(matrix(NA,nrow(dbSPEC),0))

for(nSPEC_DISC in 1:ncol(dbSPEC)){

if(length(unique(dbSPEC[,nSPEC_DISC])) >2){

dfSPEC_DISC=cbind.data.frame(dfSPEC_DISC, dbSPEC[,nSPEC_DISC,drop=F])

}

}

#vegetation PCOA

distVEG = vegdist(dbSPEC, "bray")

dbVEG_PCOA = pcoa(distVEG, correction="lingoes") #PCoA scores using Bray-Curtis dissimilarity, this can then be used in an RDA to create a distance based RDA

dfVEG_PCOA_LM = cbind(as.data.frame(dbVEG_PCOA$values[,'Rel_corr_eig']), as.data.frame(seq(1:length(dbVEG_PCOA$values[,'Rel_corr_eig']))))

colnames(dfVEG_PCOA_LM) = c('x', 'y')

lmVEG_PCOA = lm(x ~ y,data=dfVEG_PCOA_LM)

lmVEG_PCOA_SEG = segmented(lmVEG_PCOA, seg.Z=~y, psi=list(y=c(max(which(dbVEG_PCOA$values[,'Rel_corr_eig'] >0.01)))),control=seg.control(n.boot=10000))#probably a bit excessive, but it is to avoid deviation from the true break point (which can lead to drastically different results as seen on 16-01-2015 together with Amber)

nVEG_PCOA_POS = ceiling(lmVEG_PCOA_SEG$psi[2]) #round up the estimated break point to whole variables

dbVEG_PCOA=(dbVEG_PCOA$vectors.cor)[,1:nVEG_PCOA_POS]

vVEG_EXPLVAR = dfVEG_PCOA_LM[1:nVEG_PCOA_POS,1]/sum(dfVEG_PCOA_LM[1:nVEG_PCOA_POS,1])

colnames(dbVEG_PCOA) = paste("PCOA_VAR",seq(1,ncol(dbVEG_PCOA),le=ncol(dbVEG_PCOA)),sep="")#very important as otherwise there are no headers to select variables on for e.g. gradientForest function

rownames(dbVEG_PCOA) = rownames(dbSPEC)

lENVVEG_BANK = c("SOIL_PH","SOIL_C_BANK","SOIL_NTOT_BANK","SOIL_PTOT_BANK","ANGLE_MEAN",

"MORPH_WIDTH_BANK_MEAN","SOIL_MOISTURE")

#Select Soil Type variables

lSOIL_DESEL = c('Area','Site','Year_','POINT_X','POINT_Y','BOFEK2012','BOFEK_LABEL','PAWN')

dfSOIL1 = dbSOIL_RAW[,!(colnames(dbSOIL_RAW) %in% lSOIL_DESEL)]

dfSOIL2 = dfSOIL1[,which(grepl("PAWN", names(dfSOIL1))==1)]

dfSOIL3 = subset(dfSOIL2, substr(row.names(dfSOIL2), 1, 1) %in% lAREAS)

dfSOIL4 = subset(dfSOIL3, grepl(25, row.names(dfSOIL3)) != TRUE )#remove site 25 (if it exists)

dfSOIL_SEL = dfSOIL4[which(row.names(dfSOIL4) %in% lSITES_SEL),which(colnames(dfSOIL4) %in% names(which(colSums(dfSOIL4[,-1])!=0)))]

dfSOIL_SEL$SOIL_FAC = as.factor(dfSOIL4$PAWN_LABEL[which(row.names(dfSOIL4) %in% lSITES_SEL)])

#Select abiotic variables

dfENV = dbENV_RAW[,(colnames(dbENV_RAW) %in% lENVVEG_BANK)]

dfENV1 = subset(dfENV, substr(row.names(dfENV), 1, 1) %in% lAREAS)

dfENV2 = subset(dfENV1, grepl(25, row.names(dfENV1)) != TRUE )#remove site 25 (if it exists)

dfENV3 = dfENV2[which(row.names(dfENV2) %in% lSITES_SEL),which(colSums(is.na(dfENV2))==0)]

dfENV_SEL = dfENV3

dfENV_WORK = cbind.data.frame(dfENV_SEL, dfSOIL_SEL[,ncol(dfSOIL_SEL),drop=F])

dfENV_WORK_FS = cbind.data.frame(dfENV_SEL, dfSOIL_SEL[,-ncol(dfSOIL_SEL),drop=F])

#Select land use

dfLU1 = subset(dbLU_RAW, substr(row.names(dbLU_RAW), 1, 1) %in% lAREAS)

dfLU_SEL = dfLU1[which(row.names(dfLU1) %in% lSITES_SEL), c(9:12),drop=F]

dfLU_WORK_FS = dfLU_SEL

dfLU_WORK = dfLU_SEL

dfMIC_SUBSTRATE=dfMIC_CORR3[order(dfMIC_CORR3[,1]),]

#test per substrate with GF

dfMIC_SEL = data.frame(matrix(NA,nrow(dfENV_WORK_FS),0))

for(nSUBSTR in 5:ncol(dfMIC_SUBSTRATE)){

vR2 = c()

dfSUBSTR_EXPL_TIME=data.frame(matrix(NA,nrow(dfENV_WORK_FS),0))

for(nTIME in lTIME_SEL){

#--scaled per substrate, raw data

dfSUBSTR_EXPL = dfMIC_SUBSTRATE[which(dfMIC_SUBSTRATE$Timepoint %in% nTIME),nSUBSTR,drop=F]

dbMIC_PERC=data.frame()

for(nMIC_ROW in 1:nrow(dfSUBSTR_EXPL)){

dbMIC_PERC=rbind(dbMIC_PERC,(dfSUBSTR_EXPL[nMIC_ROW,]/colSums(dfSUBSTR_EXPL)))

}

dbMIC_PERC[is.na(dbMIC_PERC)]=0

dfSUBSTR_EXPL2=dbMIC_PERC

colnames(dfSUBSTR_EXPL2)=colnames(dfSUBSTR_EXPL)

dfSUBSTR_EXPL_TIME=cbind.data.frame(dfSUBSTR_EXPL_TIME, dfSUBSTR_EXPL2)

dfSUBSTR_PREDICT = cbind.data.frame(dfENV_WORK_FS,dbVEG_PCOA,dfLU_WORK_FS)

rdaSUBSTR = rda(dfSUBSTR_EXPL2,dfSUBSTR_PREDICT)

fR2_SUBSTR=RsquareAdj(rdaSUBSTR)$adj.r.squared #extract the adjusted R2

if(is.na(fR2_SUBSTR)==TRUE){fR2_SUBSTR=0}

if(fR2_SUBSTR<0){fR2_SUBSTR=0}

#gfSUBSTR_CAL = gradientForest(cbind(dfSUBSTR_PREDICT, dfSUBSTR_EXPL2), predictor.vars = colnames(dfSUBSTR_PREDICT), response.vars = colnames(dfSUBSTR_EXPL2), ntree = 999, mtry=ceiling(sqrt(ncol(dfSUBSTR_PREDICT))), maxLevel=floor(log2(nrow(dfSUBSTR_PREDICT) * 0.368/2)), corr.threshold=0.5)

vR2 = c(vR2,fR2_SUBSTR)

}

vWA = vR2/sum(vR2)

if(is.na(sum(vWA))==TRUE){

dfMIC_ADD = as.data.frame(rowMeans(dfSUBSTR_EXPL_TIME))

colnames(dfMIC_ADD) = colnames(dfSUBSTR_EXPL_TIME[,1,drop=F])

dfMIC_SEL=cbind.data.frame(dfMIC_SEL,dfMIC_ADD)

}else{

dfWA=t(as.data.frame(rep(as.data.frame(vWA), nrow(dfSUBSTR_EXPL_TIME))))

dfWA[is.na(dfWA)]=0

dfMIC_ADD = as.data.frame(rowSums(dfWA*dfSUBSTR_EXPL_TIME))

colnames(dfMIC_ADD) = colnames(dfSUBSTR_EXPL_TIME[,1,drop=F])

dfMIC_SEL=cbind.data.frame(dfMIC_SEL,dfMIC_ADD)

}

}

rownames(dfMIC_SEL)=rownames(dfENV_WORK_FS)

#Select areas

dfAREA1 = subset(dbAREA_RAW[,-1], substr(row.names(dbAREA_RAW[,-1]), 1, 1) %in% lAREAS)

dfAREA2 = subset(dfAREA1, grepl(25, row.names(dfAREA1)) != TRUE )#remove site 25 (if it exists)

dfAREA_SEL = dfAREA2[which(row.names(dfAREA2) %in% row.names(dfENV_WORK_FS)),which(colnames(dfAREA2) %in% names(which(colSums(dfAREA2[,-1])!=0)))]

dfAREA_SEL$AREA_FAC = as.factor(dfAREA2$Area[which(row.names(dfAREA2) %in% row.names(dfENV_WORK_FS))])

#---BETWEEN AREAS ANALYSIS-------------

#_______________________________________

#_____________________________________________________

#____________________________________________________________________________

nTREES=9999

nPERM=99999

#tested to see if one time point had the same explanatory power as the weighted averaged combined set; answer HELL NO!

#This enforces the idea that optimization per substrate is a good idea

#dfMIC_SEL=dfMIC_SUBSTRATE[which(dfMIC_SUBSTRATE$Timepoint %in% c(2)),-c(1:4)]

#--------MICROBIAL COMM DISTANCE MATRIX-----------------

#---Create distance matrices for between area analyses---

#We make a full data bray-curtis distance matrix and then put all site combinations within areas to zero

distMIC = vegdist(dfMIC_SEL, "bray")

dfDIST_MIC = data.matrix(distMIC)

dfDIST_SAVE=data.frame(matrix(NA,nrow(dfDIST_MIC), 0))

for(nCOL_DIST in 1:ncol(dfDIST_MIC)){

dfDIST_COL = dfDIST_MIC[,nCOL_DIST,drop=F]

dfDIST_COL[which(substr(rownames(dfDIST_COL),1,1)==substr(colnames(dfDIST_COL),1,1)),]=0

dfDIST_SAVE=cbind.data.frame(dfDIST_SAVE,dfDIST_COL)

}

distMIC_BETWEEN = as.dist(dfDIST_SAVE)

dbMIC_PCOA = pcoa(distMIC_BETWEEN, correction="lingoes") #PCoA scores using Bray-Curtis dissimilarity, this can then be used in an RDA to create a distance based RDA

dfMIC_PCOA_LM = cbind(as.data.frame(dbMIC_PCOA$values[,'Rel_corr_eig']), as.data.frame(seq(1:length(dbMIC_PCOA$values[,'Rel_corr_eig']))))

colnames(dfMIC_PCOA_LM) = c('x', 'y')

lmMIC_PCOA = lm(x ~ y,data=dfMIC_PCOA_LM)

lmMIC_PCOA_SEG = segmented(lmMIC_PCOA, seg.Z=~y, psi=list(y=c(max(which(dbMIC_PCOA$values[,'Rel_corr_eig'] >0.01)))),control=seg.control(n.boot=10000))#probably a bit excessive, but it is to avoid deviation from the true break point (which can lead to drastically different results as seen on 16-01-2015 together with Amber)

nMIC_PCOA_POS = ceiling(lmMIC_PCOA_SEG$psi[2]) #round up the estimated break point to whole variables

vMIC_EXPLVAR = dfMIC_PCOA_LM[1:nMIC_PCOA_POS,1]/sum(dfMIC_PCOA_LM[1:nMIC_PCOA_POS,1])

dbMIC_PCOA=(dbMIC_PCOA$vectors.cor)[,1:nMIC_PCOA_POS]

colnames(dbMIC_PCOA) = paste("PCOA_VAR",seq(1,ncol(dbMIC_PCOA),le=ncol(dbMIC_PCOA)),sep="")#very important as otherwise there are no headers to select variables on for e.g. gradientForest function

rownames(dbMIC_PCOA) = rownames(dfENV_WORK_FS)

#--between areas MEM--

dbXY = subset(dbXY_RAW[c(4,5)], grepl(25, row.names(dbXY_RAW)) != TRUE )#remove site 25 (if it exists)

dbXY = subset(dbXY[which(row.names(dbXY) %in% row.names(dbMIC_PCOA)),], substr(row.names(dbXY[which(row.names(dbXY) %in% row.names(dbMIC_PCOA)),]), 1, 1) %in% lAREAS) #Subselection of specific area based on area letter in lAREA

#select subset of XY by area and define distance related variables

mXY = as.matrix(dbXY) #XY coordinates

mDISTXY = dist(dbXY) #Distance matrix

#non-cut down MEMS

mDISTXY2=1 - (mDISTXY/max(mDISTXY))

lwDIST = mat2listw(as.matrix(mDISTXY2))

mMEMSPEC = scores.listw(lwDIST, echo=TRUE)

vMEMSPEC = mMEMSPEC$vectors

vMORANI = test.scores(mMEMSPEC, lwDIST, 9999)

# Save MEMs with positive spatial correlation that are significant

vMORANI_POS = which(vMORANI[,1] > -1/(nrow(vMEMSPEC)-1))

dfMEMMIC = as.data.frame(vMEMSPEC[,vMORANI_POS])

colnames(dfMEMMIC) = paste("MEM_VAR_",seq(1,ncol(dfMEMMIC),le=ncol(dfMEMMIC)),sep="")

dfSPACE_WORK = cbind.data.frame(dfMEMMIC)#,dfAREA_SEL[,1:length(lAREAS)])

dfSPACE_WORK_FS = cbind.data.frame(dfMEMMIC)#,dfAREA_SEL[,1:length(lAREAS)])

#-----------------------------------

#---GRADIENT FOREST ANALYSIS--------

#___________________________________

#Select variables using GF (VI <0.001)

#Use these variables in an RDA and varpart (no FS)

#then use a gradient forest to identify species most affected by Env, Man and space

##---GRADIENT FOREST

dfPREDICT =cbind(dfENV_WORK_FS, dfSPACE_WORK_FS, dfLU_WORK_FS, dbVEG_PCOA)#dfVEG_WORK) #untransformed vegetation data goes in here

gf_COMM= gradientForest(cbind(dfPREDICT, dbMIC_PCOA), predictor.vars = colnames(dfPREDICT), response.vars = colnames(dbMIC_PCOA), ntree = nTREES, mtry=ceiling(sqrt(ncol(dfPREDICT))), maxLevel=floor(log2(nrow(dfPREDICT) * 0.368/2)), corr.threshold=0.5)

vIMP = importance(gf_COMM, type="W")

fR2_GF_COMM = sum(importance(gf_COMM,type="S"))

gf_COMM$imp.rsq

#Idea: first run GF then RDA

#Select variables from GF with VI higher than 0.001 and use these in varpart without forsel

lVARS_GF_SEL = names(vIMP[which(vIMP>0.001)])

dfENV_SEL_GF = dfENV_WORK_FS[,which(colnames(dfENV_WORK_FS) %in% lVARS_GF_SEL),drop=F]

dfLU_SEL_GF = dfLU_WORK_FS[,which(colnames(dfLU_WORK_FS) %in% lVARS_GF_SEL),drop=F]

dfSPACE_SEL_GF = dfSPACE_WORK_FS[,which(colnames(dfSPACE_WORK_FS) %in% lVARS_GF_SEL), drop=F]

dfVEG_SEL_GF = dbVEG_PCOA[,which(colnames(dbVEG_PCOA) %in% lVARS_GF_SEL), drop=F]

#-----------------------------------

#---RDA ANALYSIS--------------------

#___________________________________

##RDA for between region differences

dfVEG_WORK =dfVEG_SEL_GF

dfVEG_SEL_GF = dfVEG_SEL_GF

dfENV_SEL_GF = dfENV_SEL_GF#cbind.data.frame(dfENV_SEL, dfSOIL_SEL[,ncol(dfSOIL_SEL),drop=F])

dfENV_SEL_GF = dfENV_SEL_GF#cbind.data.frame(dfENV_SEL, dfSOIL_SEL[,-ncol(dfSOIL_SEL),drop=F])

dfSPACE_SEL_GF = dfSPACE_SEL_GF#cbind.data.frame(dfMEMMIC, dfAREA_SEL[,4,drop=F])

dfSPACE_SEL_GF = dfSPACE_SEL_GF#cbind.data.frame(dfMEMMIC, dfAREA_SEL[,-4,drop=F])

dfLU_SEL_GF=dfLU_SEL_GF#dfLU_SEL

#Create formula's for RDA

sENV = ""

for(sVAR in colnames(dfENV_SEL_GF)){

if(sENV == ""){

sENV = sVAR

}else if(sENV != ""){

sENV = paste(sENV, sVAR, sep="+")

}

}

sSPACE = ""

for(sVAR in colnames(dfSPACE_SEL_GF)){

if(sSPACE == ""){

sSPACE = sVAR

}else if(sSPACE != ""){

sSPACE = paste(sSPACE, sVAR, sep="+")

}

}

sLU = ""

for(sVAR in colnames(dfLU_SEL_GF)){

if(sLU == ""){

sLU = sVAR

}else if(sLU != ""){

sLU = paste(sLU, sVAR, sep="+")

}

}

sVEG = ""

for(sVAR in colnames(dfVEG_SEL_GF)){

if(sVEG == ""){

sVEG = sVAR

}else if(sVEG != ""){

sVEG = paste(sVEG, sVAR, sep="+")

}

}

#sAREA_FORM = paste(" + ", "Condition(",colnames(dfAREA_SEL[,4,drop=F]),")", sep="")

fRDA_ENV = as.formula(paste('dbMIC_PCOA',' ~ ',sENV,sep=""))

fRDA_SPACE = as.formula(paste('dbMIC_PCOA',' ~ ',sSPACE,sep=""))

fRDA_LU = as.formula(paste('dbMIC_PCOA',' ~ ',sLU,sep=""))

fRDA_VEG = as.formula(paste('dbMIC_PCOA',' ~ ',sVEG,sep=""))

#--ENVIRONMENTAL+SOIL--

rdaSPEC_ENV = rda(fRDA_ENV, data=dfENV_SEL_GF)

fR2_SPEC_ENV=RsquareAdj(rdaSPEC_ENV)$adj.r.squared #extract the adjusted R2

testSPEC_ENV=anova(rdaSPEC_ENV,permutations=nPERM)

fFSTAT_SPEC_ENV=testSPEC_ENV$F[1] #extract the F statistic

fPSTAT_SPEC_ENV=testSPEC_ENV$Pr[1] #extract the p value

fsRDA_ENV = try(forward.sel(dbMIC_PCOA, dfENV_SEL_GF, nperm=nPERM, adjR2thresh=fR2_SPEC_ENV))

if(class(fsRDA_ENV)=="try-error" | fPSTAT_SPEC_ENV >0.05){lENV_FS = c()}else{lENV_FS = c(unlist(as.data.frame(fsRDA_ENV[,2])))}

#--SPATIAL--

rdaSPEC_MEM = rda(fRDA_SPACE, data=dfSPACE_SEL_GF)

fR2_SPEC_MEM=RsquareAdj(rdaSPEC_MEM)$adj.r.squared #extract the adjusted R2

testSPEC_MEM=anova(rdaSPEC_MEM,permutations=nPERM)

fFSTAT_SPEC_MEM=testSPEC_MEM$F[1] #extract the F statistic

fPSTAT_SPEC_MEM=testSPEC_MEM$Pr[1] #extract the p value

fsRDA_MEM = try(forward.sel(dbMIC_PCOA, dfSPACE_SEL_GF, nperm=nPERM, adjR2thresh=fR2_SPEC_MEM))

if(class(fsRDA_MEM)=="try-error" | fPSTAT_SPEC_MEM >0.05){lMEM_FS = c()}else{lMEM_FS = c(unlist(as.data.frame(fsRDA_MEM[,2])))}

#--MANAGEMENT--

rdaSPEC_MAN = rda(fRDA_LU, data=dfLU_SEL_GF)

fR2_SPEC_MAN=RsquareAdj(rdaSPEC_MAN)$adj.r.squared #extract the adjusted R2

testSPEC_MAN=anova(rdaSPEC_MAN,permutations =nPERM)

fFSTAT_SPEC_MAN=testSPEC_MAN$F[1] #extract the F statistic

fPSTAT_SPEC_MAN=testSPEC_MAN$Pr[1] #extract the p value

fsRDA_MAN = try(forward.sel(dbMIC_PCOA, dfLU_SEL_GF, nperm=nPERM, adjR2thresh=fR2_SPEC_MAN))

if(class(fsRDA_MAN)=="try-error" | fPSTAT_SPEC_MAN >0.05){lMAN_FS = c()}else{lMAN_FS = c(unlist(as.data.frame(fsRDA_MAN[,2])))}

#--VEGETATION--

rdaSPEC_VEG = rda(fRDA_VEG, data=as.data.frame(dfVEG_SEL_GF))

fR2_SPEC_VEG=RsquareAdj(rdaSPEC_VEG)$adj.r.squared #extract the adjusted R2

testSPEC_VEG=anova(rdaSPEC_VEG,permutations =nPERM)

fFSTAT_SPEC_VEG=testSPEC_VEG$F[1] #extract the F statistic

fPSTAT_SPEC_VEG=testSPEC_VEG$Pr[1] #extract the p value

fsRDA_VEG = try(forward.sel(dbMIC_PCOA, dfVEG_SEL_GF, nperm=nPERM,adjR2thresh=fR2_SPEC_VEG))

if(class(fsRDA_VEG)=="try-error" | fPSTAT_SPEC_VEG >0.05){lVEG_FS = c()}else{lVEG_FS = c(unlist(as.data.frame(fsRDA_VEG[,2])))}

#--COMBINED MODEL---

dfALL_WORK_FS = cbind.data.frame(dfENV_SEL_GF[,lENV_FS,drop=F], dfSPACE_SEL_GF[,lMEM_FS, drop=F], dfLU_SEL_GF[,lMAN_FS,drop=F], dfVEG_SEL_GF[,lVEG_FS,drop=F])

#dfALL_WORK_FS = cbind.data.frame(dfENV_SEL_GF, dfSPACE_SEL_GF, dfLU_SEL_GF_FS, dfVEG_SEL_GF)

#Create a total model

rdaSPEC_COMB = rda(dbMIC_PCOA~., dfALL_WORK_FS)

fR2_SPEC_COMB=RsquareAdj(rdaSPEC_COMB)$adj.r.squared #extract the adjusted R2

testSPEC_COMB=anova(rdaSPEC_COMB,permutations=nPERM)

#anova(rdaSPEC_COMB,permutations=nPERM, by='term')

fFSTAT_SPEC_COMB=testSPEC_COMB$F[1] #extract the F statistic

fPSTAT_SPEC_COMB=testSPEC_COMB$Pr[1] #extract the p value

#forward.sel(dbMIC_PCOA, dfALL_WORK_FS, nperm=nPERM,adjR2thresh=fR2_SPEC_COMB)

#vartpart plot

pdf(paste("C:/Users/Sven/Documents/PhD/R/Data/RESULTS_MIC/","BIOLOG_","VARPART_BETWEEN_POLDERS",".pdf",sep=""),width=10, height=7, pointsize=14)

if(length(lENV_FS)>0 & length(lMEM_FS)>0 & length(lMAN_FS)>0 & length(lVEG_FS)>0){

lPART = c('ENV', 'SPACE', 'FERTILIZATION', 'VEG')

plot(varpart(dbMIC_PCOA, dfENV_SEL_GF[,lENV_FS,drop=F], dfSPACE_SEL_GF[,lMEM_FS, drop=F], dfLU_SEL_GF[,lMAN_FS,drop=F], dfVEG_WORK[,lVEG_FS,drop=F]), Xnames=lPART)

}else if(length(lENV_FS)>0 & length(lMEM_FS)>0 & length(lMAN_FS)>0 ){

lPART = c('ENV', 'SPACE', 'FERTILIZATION')

plot(varpart(dbMIC_PCOA, dfENV_SEL_GF[,lENV_FS,drop=F], dfSPACE_SEL_GF[,lMEM_FS, drop=F], dfLU_SEL_GF[,lMAN_FS,drop=F]), Xnames=lPART)

}else if(length(lENV_FS)>0 & length(lMEM_FS)>0 & length(lVEG_FS)>0){

lPART = c('ENV', 'SPACE', 'VEG')

plot(varpart(dbMIC_PCOA, dfENV_SEL_GF[,lENV_FS,drop=F], dfSPACE_SEL_GF[,lMEM_FS, drop=F], dfVEG_WORK[,lVEG_FS,drop=F]), Xnames=lPART)

}else if(length(lENV_FS)>0 & length(lMAN_FS)>0 & length(lVEG_FS)>0){

lPART = c('ENV', 'FERTILIZATION', 'VEG')

plot(varpart(dbMIC_PCOA, dfENV_SEL_GF[,lENV_FS,drop=F], dfLU_SEL_GF[,lMAN_FS,drop=F], dfVEG_WORK[,lVEG_FS,drop=F]), Xnames=lPART)

}else if(length(lENV_FS)>0 & length(lMEM_FS)>0 & length(lMAN_FS)>0 & length(lVEG_FS)>0){

lPART = c('SPACE', 'FERTILIZATION', 'VEG')

plot(varpart(dbMIC_PCOA, dfSPACE_SEL_GF[,lMEM_FS, drop=F], dfLU_SEL_GF[,lMAN_FS,drop=F], dfVEG_WORK[,lVEG_FS,drop=F]), Xnames=lPART)

}else if(length(lENV_FS)>0 & length(lMEM_FS)>0 & length(lMAN_FS)>0 & length(lVEG_FS)>0){

lPART = c('ENV', 'SPACE')

plot(varpart(dbMIC_PCOA, dfENV_SEL_GF[,lENV_FS,drop=F], dfSPACE_SEL_GF[,lMEM_FS, drop=F]), Xnames=lPART)

}else if(length(lENV_FS)>0 & length(lVEG_FS)>0){

lPART = c('ENV', 'VEG')

plot(varpart(dbMIC_PCOA, dfENV_SEL_GF[,lENV_FS,drop=F], dfVEG_WORK[,lVEG_FS,drop=F]), Xnames=lPART)

}else if(length(lENV_FS)>0 & length(lMAN_FS)>0){

lPART = c('ENV', 'FERTILIZATION')

plot(varpart(dbMIC_PCOA, dfENV_SEL_GF[,lENV_FS,drop=F], dfLU_SEL_GF[,lMAN_FS,drop=F]), Xnames=lPART)

}else if(length(lMEM_FS)>0 & length(lVEG_FS)>0){

lPART = c('SPACE', 'VEG')

plot(varpart(dbMIC_PCOA, dfSPACE_SEL_GF[,lMEM_FS, drop=F], dfVEG_WORK[,lVEG_FS,drop=F]), Xnames=lPART)

}else if(length(lMEM_FS)>0 & length(lMAN_FS)>0){

lPART = c('SPACE', 'FERTILIZATION')

plot(varpart(dbMIC_PCOA, dfSPACE_SEL_GF[,lMEM_FS, drop=F], dfLU_SEL_GF[,lMAN_FS,drop=F]), Xnames=lPART)

}else if(length(lMAN_FS)>0 & length(lVEG_FS)>0){

lPART = c('FERTILIZATION', 'VEG')

plot(varpart(dbMIC_PCOA, dfLU_SEL_GF[,lMAN_FS,drop=F], dfVEG_WORK[,lVEG_FS,drop=F]), Xnames=lPART)

}else{

print('no variation partitioning possible')

}

dev.off()

##

##lPART = c('ENV', 'SPACE', 'FERTILIZATION', 'VEG')

##windows()

##plot(varpart(dbMIC_PCOA, dfENV_WORK_FS[,lENV_FS,drop=F], dfSPACE_WORK_FS[,lMEM_FS, drop=F], dfLU_WORK_FS[,lMAN_FS,drop=F], dfVEG_WORK[,lVEG_FS,drop=F]), Xnames=lPART)

##plot(varpart(dbMIC_PCOA, dfENV_WORK_FS, dfSPACE_WORK_FS, dfLU_WORK_FS, dfVEG_WORK), Xnames=lPART)

##

##windows()

##lPART = c('ENV', 'SPACE', 'VEG')

###plot(varpart(dbMIC_PCOA, dfENV_WORK_FS[,lENV_FS,drop=F], dfSPACE_WORK_FS[,lMEM_FS, drop=F], dfVEG_WORK[,lVEG_FS,drop=F]), Xnames=lPART)

##plot(varpart(dbMIC_PCOA, dfENV_WORK_FS, dfSPACE_WORK_FS, dfVEG_WORK), Xnames=lPART)

##

#--forward selected (full) and partial models (marginal partitions)

dfOUTPUT_RDA = data.frame(matrix(NA,0,5))

lMODELS_RDA = c('FULL_ENV', 'PURE_ENV','FULL_SPACE','PURE_SPACE','FULL_FERTILIZATION','PURE_FERTILIZATION','FULL_VEGETATION','PURE_VEGETATION')

for(sMODEL_RDA in lMODELS_RDA){

if(sMODEL_RDA == 'PURE_ENV'){dfEXPL = dfENV_SEL_GF[,lENV_FS,drop=F]

dfCONDITION = dfALL_WORK_FS[,-which(colnames(dfALL_WORK_FS) %in% colnames(dfEXPL))]}

if(sMODEL_RDA == 'PURE_SPACE'){dfEXPL = dfSPACE_SEL_GF[,lMEM_FS, drop=F]

dfCONDITION = dfALL_WORK_FS[,-which(colnames(dfALL_WORK_FS) %in% colnames(dfEXPL))]}

if(sMODEL_RDA == 'PURE_FERTILIZATION'){dfEXPL = dfLU_SEL_GF[,lMAN_FS,drop=F]

dfCONDITION = dfALL_WORK_FS[,-which(colnames(dfALL_WORK_FS) %in% colnames(dfEXPL))]}

if(sMODEL_RDA == 'PURE_VEGETATION'){dfEXPL = dfVEG_SEL_GF[,lVEG_FS,drop=F]

dfCONDITION = dfALL_WORK_FS[,-which(colnames(dfALL_WORK_FS) %in% colnames(dfEXPL))]}

if(sMODEL_RDA == 'FULL_ENV'){dfEXPL = dfENV_SEL_GF[,lENV_FS,drop=F]

dfCONDITION = data.frame(matrix(0,nrow(dfALL_WORK_FS),0))}

if(sMODEL_RDA == 'FULL_SPACE'){dfEXPL = dfSPACE_SEL_GF[,lMEM_FS, drop=F]

dfCONDITION = data.frame(matrix(0,nrow(dfALL_WORK_FS),0))}

if(sMODEL_RDA == 'FULL_FERTILIZATION'){dfEXPL = dfLU_SEL_GF[,lMAN_FS,drop=F]

dfCONDITION = data.frame(matrix(0,nrow(dfALL_WORK_FS),0))}

if(sMODEL_RDA == 'FULL_VEGETATION'){dfEXPL = dfVEG_SEL_GF[,lVEG_FS,drop=F]

dfCONDITION = data.frame(matrix(0,nrow(dfALL_WORK_FS),0))}

if(ncol(dfCONDITION) >0 & ncol(dfEXPL)>0){

rdaSPEC_PART = rda(dbMIC_PCOA, dfEXPL, dfCONDITION)

fR2_SPEC_PART=RsquareAdj(rdaSPEC_PART)$adj.r.squared #extract the adjusted R2

testSPEC_PART=anova(rdaSPEC_PART,permutations=nPERM)

fFSTAT_SPEC_PART=testSPEC_PART$F[1] #extract the F statistic

fPSTAT_SPEC_PART=testSPEC_PART$Pr[1] #extract the p value

sFS_VARS = paste(colnames(dfEXPL),collapse = "; ")

}else if(substr(sMODEL_RDA,1,4)=='FULL' & ncol(dfEXPL)>0){

rdaSPEC_PART = rda(dbMIC_PCOA, dfEXPL, dfCONDITION)

fR2_SPEC_PART=RsquareAdj(rdaSPEC_PART)$adj.r.squared #extract the adjusted R2

testSPEC_PART=anova(rdaSPEC_PART,permutations=nPERM)

fFSTAT_SPEC_PART=testSPEC_PART$F[1] #extract the F statistic

fPSTAT_SPEC_PART=testSPEC_PART$Pr[1] #extract the p value

sFS_VARS = paste(colnames(dfEXPL),collapse = "; ")

}else{

fR2_SPEC_PART=0

fFSTAT_SPEC_PART=0

fPSTAT_SPEC_PART=0

sFS_VARS = ""

}

dfOUT1 = t(as.data.frame(c(sMODEL_RDA, fR2_SPEC_PART,fFSTAT_SPEC_PART,fPSTAT_SPEC_PART,sFS_VARS)))

dfOUTPUT_RDA=rbind(dfOUTPUT_RDA,dfOUT1)

}

colnames(dfOUTPUT_RDA) = c('MODEL','adR2', 'F_val', 'p_val', 'FS_Variables')

write.table(dfOUTPUT_RDA,paste("C:/Users/Sven/Documents/PhD/R/Data/RESULTS_MIC/","BIOLOG_","VARPART_BETWEEN_POLDERS",".csv",sep=""), sep=',')

#make data frames expressing explained variation of different PCOA axes

dfMIC_EXPLVAR=t(as.data.frame(rep(as.data.frame(vMIC_EXPLVAR), nrow(dfIMP_STORE))))

colnames(dfMIC_EXPLVAR) = colnames(dbMIC_PCOA)

rownames(dfMIC_EXPLVAR) = colnames(dfPREDICT1)

dfVEG_EXPLVAR=as.data.frame(rep(as.data.frame(vVEG_EXPLVAR[which(colnames(dbVEG_PCOA) %in% lVARS_GF_SEL)]), ncol(dfIMP_STORE)))

colnames(dfVEG_EXPLVAR) = colnames(dbMIC_PCOA)

rownames(dfVEG_EXPLVAR) = colnames(dfVEG_SEL_GF)

dfPREDICT1 = cbind.data.frame(dfENV_WORK_FS, dfSPACE_WORK_FS, dfLU_WORK_FS, dfVEG_SEL_GF, dfSPEC_DISC)

#Do so for every model type seperately (ENV, MAN, SPACE, VEG, VEG_SPECS)

lMODEL = c('ENV', 'SPACE', 'FERTILIZATION', 'VEG', 'VEG_SPECS')

dfIMP_STORE=data.frame(matrix(NA, ncol(dfPREDICT1), ncol(dbMIC_PCOA)))

colnames(dfIMP_STORE) = colnames(dbMIC_PCOA)

rownames(dfIMP_STORE) = colnames(dfPREDICT1)

for(sMODEL in lMODEL){

if(sMODEL == 'ENV'){dfPREDICT = dfENV_WORK_FS}

if(sMODEL == 'SPACE'){dfPREDICT = dfSPACE_WORK_FS}

if(sMODEL == 'FERTILIZATION'){dfPREDICT = dfLU_WORK_FS}

if(sMODEL == 'VEG'){dfPREDICT = as.data.frame(dbVEG_PCOA)}

if(sMODEL == 'VEG_SPECS'){dfPREDICT = dfSPEC_DISC}

gf_SPEC = gradientForest(cbind(dfPREDICT, dbMIC_PCOA), predictor.vars = colnames(dfPREDICT), response.vars = colnames(dbMIC_PCOA), ntree = nTREES, mtry=ceiling(sqrt(ncol(dfPREDICT))), maxLevel=floor(log2(nrow(dfPREDICT) * 0.368/2)), corr.threshold=0.5)

(vIMP = importance(gf_SPEC, type="W"))

dfIMP_STORE_MODEL = as.data.frame(gf_SPEC$imp.rsq)

dfIMP_STORE[which(rownames(dfIMP_STORE) %in% rownames(dfIMP_STORE_MODEL)),which(colnames(dfIMP_STORE) %in% colnames(dfIMP_STORE_MODEL))] = dfIMP_STORE_MODEL

}

dfIMP_STORE[is.na(dfIMP_STORE)]=0

dfIMP_STORE=dfIMP_STORE * dfMIC_EXPLVAR

dfIMP_STORE[which(rownames(dfIMP_STORE) %in% rownames(dfVEG_EXPLVAR)),which(colnames(dfIMP_STORE) %in% colnames(dfVEG_EXPLVAR))] = dfVEG_EXPLVAR * dfIMP_STORE[which(rownames(dfIMP_STORE) %in% rownames(dfVEG_EXPLVAR)),which(colnames(dfIMP_STORE) %in% colnames(dfVEG_EXPLVAR))]

write.table(dfIMP_STORE,paste("C:/Users/Sven/Documents/PhD/R/Data/RESULTS_MIC/","BIOLOG_","GF_VIMP_BETWEEN_POLDERS",".csv",sep=""), sep=',')

#consider hanging a significance value on this using permutation testing

#----END BETWEEN AREAS----

#____________________________________________________________________________

#---WITHIN AREAS ANALYSIS-------------

#_______________________________________

#_____________________________________________________

#____________________________________________________________________________

#--------MICROBIAL COMM DISTANCE MATRIX-----------------

#---Create distance matrices for within area analyses---

lHEAD_MICPCOA=c()

for(sAREA in lAREAS){lHEAD_MICPCOA = c(lHEAD_MICPCOA, paste("PCOA_VAR",seq(1,23,le=23),"_",sAREA,sep=""))}

dfPCOA_SAVE=data.frame(matrix(NA,nrow(dfENV_WORK_FS), length(lHEAD_MICPCOA)))

colnames(dfPCOA_SAVE)=lHEAD_MICPCOA

rownames(dfPCOA_SAVE) = rownames(dfENV_WORK_FS)

vMIC_EXPLVAR=c()

for(sAREA in lAREAS){

#--scaled per plfa, raw data

dbMIC_RAW1 = dfMIC_SEL[which(substr(rownames(dfENV_WORK_FS),1,1) %in% sAREA),]

dbMIC_PERC=data.frame()

for(nMIC_ROW in 1:nrow(dbMIC_RAW1)){

dbMIC_PERC=rbind(dbMIC_PERC,(dbMIC_RAW1[nMIC_ROW,]/colSums(dbMIC_RAW1)))

}

dbMIC_RAW2=dbMIC_PERC

#Use a segmented regression to estimate the breakpoint of explained variation. Use this break point to select variables.

distMIC = vegdist(dbMIC_RAW2, "bray")

dbMIC_PCOA = pcoa(distMIC, correction="lingoes") #PCoA scores using Bray-Curtis dissimilarity, this can then be used in an RDA to create a distance based RDA

dfMIC_PCOA_LM = cbind(as.data.frame(dbMIC_PCOA$values[,'Rel_corr_eig']), as.data.frame(seq(1:length(dbMIC_PCOA$values[,'Rel_corr_eig']))))

colnames(dfMIC_PCOA_LM) = c('x', 'y')

lmMIC_PCOA = lm(x ~ y,data=dfMIC_PCOA_LM)

lmMIC_PCOA_SEG = segmented(lmMIC_PCOA, seg.Z=~y, psi=list(y=c(max(which(dbMIC_PCOA$values[,'Rel_corr_eig'] >0.05)))),control=seg.control(n.boot=10000))#probably a bit excessive, but it is to avoid deviation from the true break point (which can lead to drastically different results as seen on 16-01-2015 together with Amber)

nMIC_PCOA_POS = ceiling(lmMIC_PCOA_SEG$psi[2]) #round up the estimated break point to whole variables

dbMIC_PCOA=(dbMIC_PCOA$vectors.cor)[,1:nMIC_PCOA_POS]

vMIC_EXPLVAR = c(vMIC_EXPLVAR, dfMIC_PCOA_LM[1:nMIC_PCOA_POS,1]/sum(dfMIC_PCOA_LM[1:nMIC_PCOA_POS,1]))

colnames(dbMIC_PCOA) = paste("PCOA_VAR",seq(1,ncol(dbMIC_PCOA),le=ncol(dbMIC_PCOA)),"_",sAREA,sep="")#very important as otherwise there are no headers to select variables on for e.g. gradientForest function

rownames(dbMIC_PCOA) = rownames(dbMIC_RAW2)

dfPCOA_SAVE[which(rownames(dfPCOA_SAVE) %in% rownames(dbMIC_PCOA)),which(colnames(dfPCOA_SAVE) %in% colnames(dbMIC_PCOA))] = dbMIC_PCOA

}

dfPCOA_SAVE[is.na(dfPCOA_SAVE)]=0

if(length(which(colSums(dfPCOA_SAVE) == 0))>0){dfPCOA_SAVE = dfPCOA_SAVE[,-(which(colSums(dfPCOA_SAVE) == 0))]} #remove all zero columns

dbMIC_PCOA = dfPCOA_SAVE

#Vegetation

lHEAD_VEGPCOA=c()

for(sAREA in lAREAS){lHEAD_VEGPCOA = c(lHEAD_VEGPCOA, paste("PCOA_VAR",seq(1,23,le=23),"_",sAREA,sep=""))}

dfPCOA_SAVE=data.frame(matrix(NA,nrow(dbSPEC), length(lHEAD_VEGPCOA)))

colnames(dfPCOA_SAVE)=lHEAD_VEGPCOA

rownames(dfPCOA_SAVE) = rownames(dbSPEC)

vVEG_EXPLVAR=c()

for(sAREA in lAREAS){

dbSPEC1 = dbSPEC[which(substr(rownames(dbSPEC),1,1) %in% sAREA),]

distVEG = vegdist(dbSPEC1, "bray")

dbVEG_PCOA = pcoa(distVEG, correction="lingoes") #PCoA scores using Bray-Curtis dissimilarity, this can then be used in an RDA to create a distance based RDA

dfVEG_PCOA_LM = cbind(as.data.frame(dbVEG_PCOA$values[,'Rel_corr_eig']), as.data.frame(seq(1:length(dbVEG_PCOA$values[,'Rel_corr_eig']))))

colnames(dfVEG_PCOA_LM) = c('x', 'y')

lmVEG_PCOA = lm(x ~ y,data=dfVEG_PCOA_LM)

lmVEG_PCOA_SEG = segmented(lmVEG_PCOA, seg.Z=~y, psi=list(y=c(max(which(dbVEG_PCOA$values[,'Rel_corr_eig'] >0.05)))),control=seg.control(n.boot=10000))#probably a bit excessive, but it is to avoid deviation from the true break point (which can lead to drastically different results as seen on 16-01-2015 together with Amber)

nVEG_PCOA_POS = ceiling(lmVEG_PCOA_SEG$psi[2]) #round up the estimated break point to whole variables

dbVEG_PCOA=(dbVEG_PCOA$vectors.cor)[,1:nVEG_PCOA_POS]

vVEG_EXPLVAR = c(vVEG_EXPLVAR, dfVEG_PCOA_LM[1:nVEG_PCOA_POS,1]/sum(dfVEG_PCOA_LM[1:nVEG_PCOA_POS,1]))

colnames(dbVEG_PCOA) = paste("PCOA_VAR",seq(1,ncol(dbVEG_PCOA),le=ncol(dbVEG_PCOA)),"_",sAREA,sep="")#very important as otherwise there are no headers to select variables on for e.g. gradientForest function

rownames(dbVEG_PCOA) = rownames(dbSPEC1)

dfPCOA_SAVE[which(rownames(dfPCOA_SAVE) %in% rownames(dbVEG_PCOA)),which(colnames(dfPCOA_SAVE) %in% colnames(dbVEG_PCOA))] = dbVEG_PCOA

}

dfPCOA_SAVE[is.na(dfPCOA_SAVE)]=0

if(length(which(colSums(dfPCOA_SAVE) == 0))>0){dfPCOA_SAVE = dfPCOA_SAVE[,-(which(colSums(dfPCOA_SAVE) == 0))]} #remove all zero columns

dbVEG_PCOA = dfPCOA_SAVE

#--within areas MEM--

dfMEMMIC=data.frame(matrix(0,nrow(dfAREA_SEL),180))

rownames(dfMEMMIC)=rownames(dfAREA_SEL)

lMEM_HEAD=c()

for(sAREA in lAREAS){

dbXY = subset(dbXY_RAW[c(4,5)], grepl(25, row.names(dbXY_RAW)) != TRUE )#remove site 25 (if it exists)

dbXY = dbXY[which(row.names(dbXY) %in% row.names(dbMIC_PCOA)),]

dbXY = subset(dbXY, substr(row.names(dbXY), 1, 1) %in% sAREA) #Subselection of specific area based on area letter in lAREA

#select subset of XY by area and define distance related variables

mXY = as.matrix(dbXY) #XY coordinates

mDISTXY = dist(dbXY) #Distance matrix

## fDIST = function(D, dmax, y) {1 - (D/dmax)} #Weighing function for spatial weighing (B)

## (lTHRESH <- seq(give.thresh(mDISTXY), to=max(mDISTXY), le=10000))

## #(lTHRESH <- seq(149584, to=149584, le=1))

## nbCON <- lapply(lTHRESH, dnearneigh, x=mXY, d1=0)

##

## dbMIC = subset(dbMIC_PCOA, substr(row.names(dbMIC_PCOA), 1, 1) %in% sAREA)

##

## memTHRESH <- lapply(nbCON, function(x) test.W(x, Y=dbMIC, f=fDIST, dmax=max(mDISTXY), xy=mXY, MEM.autocor = c("positive")))

## memAIC_MIN <- sapply(memTHRESH, function(x) min(x$best$AICc, na.rm=TRUE))

##

## memMEMMODEL <- unlist(memTHRESH[which.min(memAIC_MIN)], recursive=FALSE)

## # MEM variables selected in the best model

## lMEMID <- memMEMMODEL$best$ord

## vMEM_TOP <- memMEMMODEL$best$vectors[,c(lMEMID)]

## # Adjusted R2 of best model

## RsquareAdj(memMEMMODEL$best$R2[which.min(memMEMMODEL$best$AICc)], nrow(dbMIC), length(lMEMID))

## vMEM_USE = as.data.frame(vMEM_TOP)

## colnames(vMEM_USE) = paste("MEM_VAR_",sAREA,"_",seq(1,ncol(vMEM_USE),le=ncol(vMEM_USE)),sep="")

## nMEM_MINCOL = min(which(colSums(dfMEMMIC)==0))

## if(is.infinite(nMEM_MINCOL)==TRUE){nMEM_MINCOL=1}

## dfMEMMIC[which(grepl(sAREA, rownames(dfMEMMIC))==1),c(nMEM_MINCOL:(nMEM_MINCOL+ncol(vMEM_USE)-1))] = vMEM_USE

## lMEM_HEAD = c(lMEM_HEAD,colnames(vMEM_USE))

#non-cut down MEMS

mDISTXY2=1 - (mDISTXY/max(mDISTXY))

lwDIST = mat2listw(as.matrix(mDISTXY2))

mMEMSPEC = scores.listw(lwDIST, echo=TRUE)

vMEMSPEC = mMEMSPEC$vectors

vMORANI = test.scores(mMEMSPEC, lwDIST, 1000)

# Save MEMs with positive spatial correlation that are significant

vMORANI_POS = which(vMORANI[,1] > -1/(nrow(vMEMSPEC)-1))

vMEM_USE = as.data.frame(vMEMSPEC[,vMORANI_POS])

colnames(vMEM_USE) = paste("MEM_VAR_",sAREA,"_",seq(1,ncol(vMEM_USE),le=ncol(vMEM_USE)),sep="")

nMEM_MINCOL = min(which(colSums(dfMEMMIC)==0))

if(is.infinite(nMEM_MINCOL)==TRUE){nMEM_MINCOL=1}

dfMEMMIC[which(grepl(sAREA, rownames(dfMEMMIC))==1),c(nMEM_MINCOL:(nMEM_MINCOL+ncol(vMEM_USE)-1))] = vMEM_USE

lMEM_HEAD = c(lMEM_HEAD,colnames(vMEM_USE))

}

dfMEMMIC=dfMEMMIC[,which(colSums(dfMEMMIC)!=0)]

colnames(dfMEMMIC)=lMEM_HEAD

dfSPACE_WORK = cbind.data.frame(dfMEMMIC)

dfSPACE_WORK_FS = cbind.data.frame(dfMEMMIC)

#-----------------------------------

#---GRADIENT FOREST ANALYSIS--------

#___________________________________

#Select variables using GF (VI <0.001)

#Use these variables in an RDA and varpart

#run for all data

dfPREDICT =cbind(dfENV_WORK_FS, dfSPACE_WORK_FS, dfLU_WORK, dbVEG_PCOA)

gf_COMM= gradientForest(cbind(dfPREDICT, dbMIC_PCOA), predictor.vars = colnames(dfPREDICT), response.vars = colnames(dbMIC_PCOA), ntree = nTREES, mtry=ceiling(sqrt(ncol(dfPREDICT))), maxLevel=floor(log2(nrow(dfPREDICT) * 0.368/2)), corr.threshold=0.5)

vIMP = importance(gf_COMM, type="W")

#Select variables from GF with VI higher than 0.001 and use these in varpart without forsel

lVARS_GF_SEL = names(vIMP[which(vIMP>0.001)])

dfENV_SEL_GF = dfENV_WORK_FS[,which(colnames(dfENV_WORK_FS) %in% lVARS_GF_SEL),drop=F]

dfLU_SEL_GF = dfLU_WORK[,which(colnames(dfLU_WORK) %in% lVARS_GF_SEL),drop=F]

dfSPACE_SEL_GF = dfSPACE_WORK_FS[,which(colnames(dfSPACE_WORK_FS) %in% lVARS_GF_SEL), drop=F]

dfVEG_SEL_GF = dbVEG_PCOA[,which(colnames(dbVEG_PCOA) %in% lVARS_GF_SEL), drop=F]

#-----------------------------------

#---RDA ANALYSIS--------------------

#___________________________________

##RDA for between region differences

#Create formula's for RDA

sENV = ""

for(sVAR in colnames(dfENV_SEL_GF)){

if(sENV == ""){

sENV = sVAR

}else if(sENV != ""){

sENV = paste(sENV, sVAR, sep="+")

}

}

sSPACE = ""

for(sVAR in colnames(dfSPACE_SEL_GF)){

if(sSPACE == ""){

sSPACE = sVAR

}else if(sSPACE != ""){

sSPACE = paste(sSPACE, sVAR, sep="+")

}

}

sLU = ""

for(sVAR in colnames(dfLU_SEL_GF)){

if(sLU == ""){

sLU = sVAR

}else if(sLU != ""){

sLU = paste(sLU, sVAR, sep="+")

}

}

sVEG = ""

for(sVAR in colnames(dfVEG_SEL_GF)){

if(sVEG == ""){

sVEG = sVAR

}else if(sVEG != ""){

sVEG = paste(sVEG, sVAR, sep="+")

}

}

sAREA_FORM = paste(" + ", "Condition(",colnames(dfAREA_SEL[,4,drop=F]),")", sep="")

fRDA_ENV = as.formula(paste('dbMIC_PCOA',' ~ ',sENV,sep=""))

fRDA_SPACE = as.formula(paste('dbMIC_PCOA',' ~ ',sSPACE,sep=""))

fRDA_LU = as.formula(paste('dbMIC_PCOA',' ~ ',sLU,sep=""))

fRDA_VEG = as.formula(paste('dbMIC_PCOA',' ~ ',sVEG,sep=""))

#--ENVIRONMENTAL+SOIL--

dfVEG_WORK =dfVEG_SEL_GF

rdaSPEC_ENV = rda(fRDA_ENV, data=cbind(dfENV_SEL_GF,dfAREA_SEL))

fR2_SPEC_ENV=RsquareAdj(rdaSPEC_ENV)$adj.r.squared #extract the adjusted R2

testSPEC_ENV=anova(rdaSPEC_ENV,permutations=nPERM)

# anova(rdaSPEC_ENV,permutations =nPERM,by='term')

fFSTAT_SPEC_ENV=testSPEC_ENV$F[1] #extract the F statistic

fPSTAT_SPEC_ENV=testSPEC_ENV$Pr[1] #extract the p value

fsRDA_ENV = try(forward.sel(dbMIC_PCOA, dfENV_SEL_GF, nperm=nPERM,adjR2thresh=fR2_SPEC_ENV))

if(class(fsRDA_ENV)=="try-error"| fPSTAT_SPEC_ENV >0.05){lENV_FS = c()}else{lENV_FS = c(unlist(as.data.frame(fsRDA_ENV[,2])))}

#--SPATIAL--

rdaSPEC_MEM = rda(fRDA_SPACE, data=cbind(dfSPACE_SEL_GF,dfAREA_SEL))

fR2_SPEC_MEM=RsquareAdj(rdaSPEC_MEM)$adj.r.squared #extract the adjusted R2

testSPEC_MEM=anova(rdaSPEC_MEM,permutations=nPERM)

# anova(rdaSPEC_MEM,permutations =nPERM,by='term')

fFSTAT_SPEC_MEM=testSPEC_MEM$F[1] #extract the F statistic

fPSTAT_SPEC_MEM=testSPEC_MEM$Pr[1] #extract the p value

fsRDA_MEM = try(forward.sel(dbMIC_PCOA, dfSPACE_SEL_GF, nperm=nPERM,adjR2thresh=fR2_SPEC_MEM))

if(class(fsRDA_MEM)=="try-error"| fPSTAT_SPEC_MEM >0.05){lMEM_FS = c()}else{lMEM_FS = c(unlist(as.data.frame(fsRDA_MEM[,2])))}

#--MANAGEMENT--

rdaSPEC_MAN = rda(fRDA_LU, data=cbind(dfLU_SEL_GF,dfAREA_SEL))

fR2_SPEC_MAN=RsquareAdj(rdaSPEC_MAN)$adj.r.squared #extract the adjusted R2

testSPEC_MAN=anova(rdaSPEC_MAN,permutations =nPERM)

# anova(rdaSPEC_MAN,permutations =nPERM,by='term')

fFSTAT_SPEC_MAN=testSPEC_MAN$F[1] #extract the F statistic

fPSTAT_SPEC_MAN=testSPEC_MAN$Pr[1] #extract the p value

fsRDA_MAN = try(forward.sel(dbMIC_PCOA, dfLU_SEL_GF, nperm=nPERM,adjR2thresh=fR2_SPEC_MAN))

if(class(fsRDA_MAN)=="try-error"| fPSTAT_SPEC_MAN >0.05){lMAN_FS = c()}else{lMAN_FS = c(unlist(as.data.frame(fsRDA_MAN[,2])))}

#--VEGETATION--

#rdaSPEC_VEG = rda(dbMIC_PCOA~., as.data.frame(dbVEG_PCOA))

rdaSPEC_VEG = rda(fRDA_VEG, data=cbind(dfVEG_WORK,dfAREA_SEL))

fR2_SPEC_VEG=RsquareAdj(rdaSPEC_VEG)$adj.r.squared #extract the adjusted R2

testSPEC_VEG=anova(rdaSPEC_VEG,permutations =nPERM)

# anova(rdaSPEC_VEG,permutations =nPERM,by='term')

fFSTAT_SPEC_VEG=testSPEC_VEG$F[1] #extract the F statistic

fPSTAT_SPEC_VEG=testSPEC_VEG$Pr[1] #extract the p value

#fsRDA_VEG = try(forward.sel(dbMIC_PCOA, dfVEG_WORK, nperm=nPERM,adjR2thresh=fR2_SPEC_VEG))

fsRDA_VEG = try(forward.sel(dbMIC_PCOA, dfVEG_WORK, nperm=nPERM,adjR2thresh=fR2_SPEC_VEG))

if(class(fsRDA_VEG)=="try-error"| fPSTAT_SPEC_VEG >0.05){lVEG_FS = c()}else{lVEG_FS = c(unlist(as.data.frame(fsRDA_VEG[,2])))}

#--COMBINED MODEL---

dfALL_WORK_FS = cbind.data.frame(dfENV_SEL_GF[,lENV_FS,drop=F], dfSPACE_SEL_GF[,lMEM_FS, drop=F], dfLU_SEL_GF[,lMAN_FS,drop=F], dfVEG_WORK[,lVEG_FS,drop=F])

#dfALL_WORK_FS = cbind.data.frame(dfENV_SEL_GF, dfSPACE_SEL_GF, dfLU_SEL_GF, dfVEG_SEL_GF)

#Create a total model

rdaSPEC_COMB = rda(dbMIC_PCOA~., dfALL_WORK_FS)

fR2_SPEC_COMB=RsquareAdj(rdaSPEC_COMB)$adj.r.squared #extract the adjusted R2

testSPEC_COMB=anova(rdaSPEC_COMB,permutations=nPERM)

#anova(rdaSPEC_COMB,permutations=nPERM, by='term')

fFSTAT_SPEC_COMB=testSPEC_COMB$F[1] #extract the F statistic

fPSTAT_SPEC_COMB=testSPEC_COMB$Pr[1] #extract the p value

#forward.sel(dbMIC_PCOA, dfALL_WORK_FS, nperm=nPERM,adjR2thresh=fR2_SPEC_COMB)

#---VAR PART---

#vartpart plot

pdf(paste("C:/Users/Sven/Documents/PhD/R/Data/RESULTS_MIC/","BIOLOG_","VARPART_WITHIN_POLDERS",".pdf",sep=""),width=10, height=7, pointsize=14)

if(length(lENV_FS)>0 & length(lMEM_FS)>0 & length(lMAN_FS)>0 & length(lVEG_FS)>0){

lPART = c('ENV', 'SPACE', 'FERTILIZATION', 'VEG')

plot(varpart(dbMIC_PCOA, dfENV_SEL_GF[,lENV_FS,drop=F], dfSPACE_SEL_GF[,lMEM_FS, drop=F], dfLU_SEL_GF[,lMAN_FS,drop=F], dfVEG_WORK[,lVEG_FS,drop=F]), Xnames=lPART)

}else if(length(lENV_FS)>0 & length(lMEM_FS)>0 & length(lMAN_FS)>0 ){

lPART = c('ENV', 'SPACE', 'FERTILIZATION')

plot(varpart(dbMIC_PCOA, dfENV_SEL_GF[,lENV_FS,drop=F], dfSPACE_SEL_GF[,lMEM_FS, drop=F], dfLU_SEL_GF[,lMAN_FS,drop=F]), Xnames=lPART)

}else if(length(lENV_FS)>0 & length(lMEM_FS)>0 & length(lVEG_FS)>0){

lPART = c('ENV', 'SPACE', 'VEG')

plot(varpart(dbMIC_PCOA, dfENV_SEL_GF[,lENV_FS,drop=F], dfSPACE_SEL_GF[,lMEM_FS, drop=F], dfVEG_WORK[,lVEG_FS,drop=F]), Xnames=lPART)

}else if(length(lENV_FS)>0 & length(lMAN_FS)>0 & length(lVEG_FS)>0){

lPART = c('ENV', 'FERTILIZATION', 'VEG')

plot(varpart(dbMIC_PCOA, dfENV_SEL_GF[,lENV_FS,drop=F], dfLU_SEL_GF[,lMAN_FS,drop=F], dfVEG_WORK[,lVEG_FS,drop=F]), Xnames=lPART)

}else if(length(lENV_FS)>0 & length(lMEM_FS)>0 & length(lMAN_FS)>0 & length(lVEG_FS)>0){

lPART = c('SPACE', 'FERTILIZATION', 'VEG')

plot(varpart(dbMIC_PCOA, dfSPACE_SEL_GF[,lMEM_FS, drop=F], dfLU_SEL_GF[,lMAN_FS,drop=F], dfVEG_WORK[,lVEG_FS,drop=F]), Xnames=lPART)

}else if(length(lENV_FS)>0 & length(lMEM_FS)>0 & length(lMAN_FS)>0 & length(lVEG_FS)>0){

lPART = c('ENV', 'SPACE')

plot(varpart(dbMIC_PCOA, dfENV_SEL_GF[,lENV_FS,drop=F], dfSPACE_SEL_GF[,lMEM_FS, drop=F]), Xnames=lPART)

}else if(length(lENV_FS)>0 & length(lVEG_FS)>0){

lPART = c('ENV', 'VEG')

plot(varpart(dbMIC_PCOA, dfENV_SEL_GF[,lENV_FS,drop=F], dfVEG_WORK[,lVEG_FS,drop=F]), Xnames=lPART)

}else if(length(lENV_FS)>0 & length(lMAN_FS)>0){

lPART = c('ENV', 'FERTILIZATION')

plot(varpart(dbMIC_PCOA, dfENV_SEL_GF[,lENV_FS,drop=F], dfLU_SEL_GF[,lMAN_FS,drop=F]), Xnames=lPART)

}else if(length(lMEM_FS)>0 & length(lVEG_FS)>0){

lPART = c('SPACE', 'VEG')

plot(varpart(dbMIC_PCOA, dfSPACE_SEL_GF[,lMEM_FS, drop=F], dfVEG_WORK[,lVEG_FS,drop=F]), Xnames=lPART)

}else if(length(lMEM_FS)>0 & length(lMAN_FS)>0){

lPART = c('SPACE', 'FERTILIZATION')

plot(varpart(dbMIC_PCOA, dfSPACE_SEL_GF[,lMEM_FS, drop=F], dfLU_SEL_GF[,lMAN_FS,drop=F]), Xnames=lPART)

}else if(length(lMAN_FS)>0 & length(lVEG_FS)>0){

lPART = c('FERTILIZATION', 'VEG')

plot(varpart(dbMIC_PCOA, dfLU_SEL_GF[,lMAN_FS,drop=F], dfVEG_WORK[,lVEG_FS,drop=F]), Xnames=lPART)

}else{

print('no variation partitioning possible')

}

dev.off()

##lPART = c('ENV', 'SPACE', 'MANAG', 'VEG')

##windows()

##plot(varpart(dbMIC_PCOA, dfENV_SEL_GF[,lENV_FS,drop=F], dfSPACE_SEL_GF[,lMEM_FS, drop=F], dfLU_SEL_GF_FS[,lMAN_FS,drop=F], dbVEG_PCOA[,lVEG_FS,drop=F]), Xnames=lPART)

##plot(varpart(dbMIC_PCOA, dfENV_SEL_GF, dfSPACE_SEL_GF, dfLU_SEL_GF, dbVEG_PCOA), Xnames=lPART)

##

##windows()

##lPART = c('SPACE', 'VEG')

##plot(varpart(dbMIC_PCOA, dfSPACE_SEL_GF[,lMEM_FS,drop=F], dbVEG_PCOA[,lVEG_FS,drop=F]), Xnames=lPART)

###plot(varpart(dbMIC_PCOA, dfENV_SEL_GF, dfSPACE_SEL_GF, dfVEG_SEL_GF), Xnames=lPART)

#--forward selected (full) and partial models (marginal partitions)

dfOUTPUT_RDA = data.frame(matrix(NA,0,5))

lMODELS_RDA = c('FULL_ENV', 'PURE_ENV','FULL_SPACE','PURE_SPACE','FULL_FERTILIZATION','PURE_FERTILIZATION','FULL_VEGETATION','PURE_VEGETATION')

##if(length(lENV_FS)>0){lMODELS_RDA = c(lMODELS_RDA, 'PURE_ENV')}

##if(length(lMEM_FS)>0){lMODELS_RDA = c(lMODELS_RDA, 'PURE_SPACE')}

##if(length(lMAN_FS)>0){lMODELS_RDA = c(lMODELS_RDA, 'PURE_FERTILIZATION')}

##if(length(lVEG_FS)>0){lMODELS_RDA = c(lMODELS_RDA, 'PURE_VEGETATION')}

for(sMODEL_RDA in lMODELS_RDA){

if(sMODEL_RDA == 'PURE_ENV'){dfEXPL = dfENV_SEL_GF[,lENV_FS,drop=F]

dfCONDITION = dfALL_WORK_FS[,-which(colnames(dfALL_WORK_FS) %in% colnames(dfEXPL))]}

if(sMODEL_RDA == 'PURE_SPACE'){dfEXPL = dfSPACE_SEL_GF[,lMEM_FS, drop=F]

dfCONDITION = dfALL_WORK_FS[,-which(colnames(dfALL_WORK_FS) %in% colnames(dfEXPL))]}

if(sMODEL_RDA == 'PURE_FERTILIZATION'){dfEXPL = dfLU_SEL_GF[,lMAN_FS,drop=F]

dfCONDITION = dfALL_WORK_FS[,-which(colnames(dfALL_WORK_FS) %in% colnames(dfEXPL))]}

if(sMODEL_RDA == 'PURE_VEGETATION'){dfEXPL = dfVEG_WORK[,lVEG_FS,drop=F]

dfCONDITION = dfALL_WORK_FS[,-which(colnames(dfALL_WORK_FS) %in% colnames(dfEXPL))]}

if(sMODEL_RDA == 'FULL_ENV'){dfEXPL = dfENV_SEL_GF[,lENV_FS,drop=F]

dfCONDITION = data.frame(matrix(0,nrow(dfALL_WORK_FS),0))}

if(sMODEL_RDA == 'FULL_SPACE'){dfEXPL = dfSPACE_SEL_GF[,lMEM_FS, drop=F]

dfCONDITION = data.frame(matrix(0,nrow(dfALL_WORK_FS),0))}

if(sMODEL_RDA == 'FULL_FERTILIZATION'){dfEXPL = dfLU_SEL_GF[,lMAN_FS,drop=F]

dfCONDITION = data.frame(matrix(0,nrow(dfALL_WORK_FS),0))}

if(sMODEL_RDA == 'FULL_VEGETATION'){dfEXPL = dfVEG_WORK[,lVEG_FS,drop=F]

dfCONDITION = data.frame(matrix(0,nrow(dfALL_WORK_FS),0))}

if(ncol(dfCONDITION) >0 & ncol(dfEXPL)>0){

rdaSPEC_PART = rda(dbMIC_PCOA, dfEXPL, dfCONDITION)

varpart(dbMIC_PCOA, dfVEG_WORK[,lVEG_FS,drop=F],dfSPACE_WORK_FS[,lMEM_FS, drop=F])

fR2_SPEC_PART=RsquareAdj(rdaSPEC_PART)$adj.r.squared #extract the adjusted R2

testSPEC_PART=anova(rdaSPEC_PART,permutations=nPERM)

fFSTAT_SPEC_PART=testSPEC_PART$F[1] #extract the F statistic

fPSTAT_SPEC_PART=testSPEC_PART$Pr[1] #extract the p value

sFS_VARS = paste(colnames(dfEXPL),collapse = "; ")

}else if(substr(sMODEL_RDA,1,4)=='FULL' & ncol(dfEXPL)>0){

rdaSPEC_PART = rda(dbMIC_PCOA, dfEXPL, dfCONDITION)

fR2_SPEC_PART=RsquareAdj(rdaSPEC_PART)$adj.r.squared #extract the adjusted R2

testSPEC_PART=anova(rdaSPEC_PART,permutations=nPERM)

fFSTAT_SPEC_PART=testSPEC_PART$F[1] #extract the F statistic

fPSTAT_SPEC_PART=testSPEC_PART$Pr[1] #extract the p value

sFS_VARS = paste(colnames(dfEXPL),collapse = "; ")

}else{

fR2_SPEC_PART=0

fFSTAT_SPEC_PART=0

fPSTAT_SPEC_PART=0

sFS_VARS = ""

}

dfOUT1 = t(as.data.frame(c(sMODEL_RDA, fR2_SPEC_PART,fFSTAT_SPEC_PART,fPSTAT_SPEC_PART,sFS_VARS)))

dfOUTPUT_RDA=rbind(dfOUTPUT_RDA,dfOUT1)

}

colnames(dfOUTPUT_RDA) = c('MODEL','adR2', 'F_val', 'p_val', 'FS_Variables')

write.table(dfOUTPUT_RDA,paste("C:/Users/Sven/Documents/PhD/R/Data/RESULTS_MIC/","BIOLOG_","VARPART_WITHIN_POLDERS",".csv",sep=""), sep=',')

#---PCOA variant

#run a gradient forest to determine variable importance

dfPREDICT1 = cbind.data.frame(dfENV_WORK_FS, dfSPACE_WORK_FS, dfLU_WORK_FS, dfVEG_SEL_GF, dfSPEC_DISC)

#Do so for every model type seperately (ENV, MAN, SPACE, VEG)

lMODEL = c('ENV', 'SPACE', 'FERTILIZATION', 'VEG', 'VEG_SPECS')

dfIMP_STORE=data.frame(matrix(NA, ncol(dfPREDICT1), ncol(dbMIC_PCOA)))

colnames(dfIMP_STORE) = colnames(dbMIC_PCOA)

rownames(dfIMP_STORE) = colnames(dfPREDICT1)

#make data frames expressing explained variation of different PCOA axes

dfMIC_EXPLVAR=t(as.data.frame(rep(as.data.frame(vMIC_EXPLVAR), nrow(dfIMP_STORE))))

colnames(dfMIC_EXPLVAR) = colnames(dbMIC_PCOA)

rownames(dfMIC_EXPLVAR) = colnames(dfPREDICT1)

dfVEG_EXPLVAR=as.data.frame(rep(as.data.frame(vVEG_EXPLVAR[which(colnames(dbVEG_PCOA) %in% lVARS_GF_SEL)]), ncol(dfIMP_STORE)))

colnames(dfVEG_EXPLVAR) = colnames(dbMIC_PCOA)

rownames(dfVEG_EXPLVAR) = colnames(dfVEG_SEL_GF)

for(sAREA in lAREAS){

for(sMODEL in lMODEL){

if(sMODEL == 'ENV'){dfPREDICT = dfENV_WORK_FS[which(substr(rownames(dfENV_WORK_FS),1,1) %in% sAREA),]}

if(sMODEL == 'SPACE'){dfPREDICT = dfSPACE_WORK_FS[which(substr(rownames(dfENV_WORK_FS),1,1) %in% sAREA),]}

if(sMODEL == 'FERTILIZATION'){dfPREDICT = dfLU_WORK_FS[which(substr(rownames(dfENV_WORK_FS),1,1) %in% sAREA),]}

if(sMODEL == 'VEG'){dfPREDICT = as.data.frame(dbVEG_PCOA)[which(substr(rownames(dfENV_WORK_FS),1,1) %in% sAREA),]}

if(sMODEL == 'VEG_SPECS'){dfPREDICT = dfSPEC_DISC[which(substr(rownames(dfENV_WORK_FS),1,1) %in% sAREA),]}

dfPREDICT = dfPREDICT[,which((colSums(dfPREDICT==0)<nrow(dfPREDICT)-1))] #remove values with no variation (e.g. dummies that are all 0/1)

gf_SPEC = gradientForest(cbind(dfPREDICT, dbMIC_PCOA[which(substr(rownames(dfENV_WORK_FS),1,1) %in% sAREA),]), predictor.vars = colnames(dfPREDICT), response.vars = colnames(dbMIC_PCOA), ntree = nTREES, mtry=ceiling(sqrt(ncol(dfPREDICT))), maxLevel=floor(log2(nrow(dfPREDICT) * 0.368/2)), corr.threshold=0.5)

if(is.null(gf_SPEC)==FALSE){

(vIMP = importance(gf_SPEC, type="W"))

dfIMP_STORE_MODEL = as.data.frame(gf_SPEC$imp.rsq)

}else{

dfIMP_STORE_MODEL=data.frame(matrix(0,ncol(dfPREDICT), ncol(dbMIC_PCOA)))

colnames(dfIMP_STORE_MODEL) = colnames(dbMIC_PCOA)

rownames(dfIMP_STORE_MODEL) = colnames(dfPREDICT)

}

dfIMP_STORE[which(rownames(dfIMP_STORE) %in% rownames(dfIMP_STORE_MODEL)),which(colnames(dfIMP_STORE) %in% colnames(dfIMP_STORE_MODEL))] = dfIMP_STORE_MODEL

}

}

dfIMP_STORE[is.na(dfIMP_STORE)]=0

dfIMP_STORE=dfIMP_STORE * dfMIC_EXPLVAR

dfIMP_STORE[which(rownames(dfIMP_STORE) %in% rownames(dfVEG_EXPLVAR)),which(colnames(dfIMP_STORE) %in% colnames(dfVEG_EXPLVAR))] = dfVEG_EXPLVAR * dfIMP_STORE[which(rownames(dfIMP_STORE) %in% rownames(dfVEG_EXPLVAR)),which(colnames(dfIMP_STORE) %in% colnames(dfVEG_EXPLVAR))]

write.table(dfIMP_STORE, paste("C:/Users/Sven/Documents/PhD/R/Data/RESULTS_MIC/","BIOLOG_","GF_VIMP_WITHIN_POLDERS",".csv",sep=""), sep=',')

#______________________________

#---END WITHIN AREAS BIOLOG

#_____________
